# Pyrazole-Thiazole Hybrids: Synthesis and Biological Evaluation against *Trypanosoma cruzi* and *Mycobacterium tuberculosis*

**DOI:** 10.1021/acsomega.5c12469

**Published:** 2026-04-01

**Authors:** Cynthia Nathália Pereira, Lorraine Martins Rocha Orlando, Edinaldo Castro de Oliveira, Mirian Claudia de Souza Pereira, Christian S. Canales Carnero, Oswaldo Julio Ramirez Delgado, Cesar Augusto Roque-Borda, Fernando Rogério Pavan, Maurício Silva dos Santos

**Affiliations:** 1 Laboratório de Síntese de Sistemas Heterocíclicos (LaSSH), Institute of Physics and Chemistry, Federal University of Itajubá, 1303 BPS Avenue, Pinheirinho, Itajubá, MG 37500-903, Brazil; 2 Laboratório de Ultraestrutura Celular, Instituto Oswaldo Cruz, Fiocruz, 4365 Brasil Avenue, Rio de Janeiro, RJ 21040-900, Brazil; 3 BIOMET Laboratory, 113086 National University of Engineering, Rimac, Lima 15333, Peru; 4 School of Pharmaceutical Sciences, São Paulo State University (UNESP), Araraquara 14800903, Brazil; 5 Vicerrectorado de Investigación, Universidad Católica de Santa María, Arequipa 04000, Peru

## Abstract

Pyrazole–thiazole hybrids **1­(a–k)** were synthesized and fully characterized to investigate their biological activities against *Trypanosoma cruzi* and *Mycobacterium tuberculosis* . Physicochemical profiling confirmed favorable drug-like properties across the series. Biological assays identified compound **1g** (3-F) as the most active against *T. cruzi*, with IC_50_ values of 11.66 μM (SI > 42.8) in trypomastigotes and 26.83 μM (SI > 18.9) in amastigotes. Compound **1h** (4-Br) displayed significant antimycobacterial activity, with a MIC of 32.28 μM against the H37Rv strain. *In silico* analyses suggested preferential stabilization of halogenated derivatives through hydrophobic and π-driven interactions in structurally related enzymatic pockets, used here as an exploratory model. Taken together, these findings demonstrate that halogen substitution strongly modulates affinity and biological performance, supporting pyrazole–thiazole hybrids as promising scaffolds for further optimization against neglected-parasite pathogens. Future studies expanding halogen patterns and probing alternative binding pockets may improve potency and target selectivity across this chemical space.

## Graphical abstract

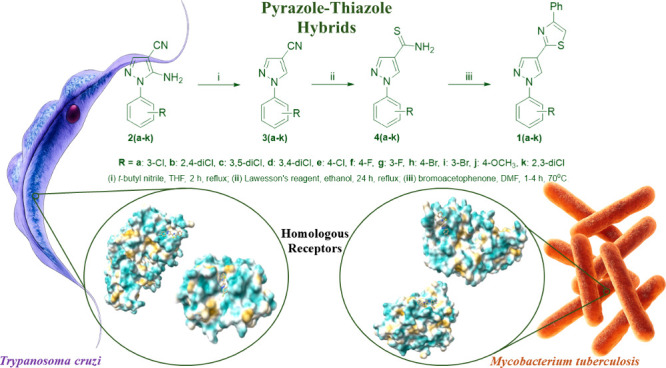

## 1 Introduction

Heterocycles are chemical structures containing at least one heteroatom, mainly nitrogen, oxygen, or sulfur, within the cyclic framework.^1^ These privileged scaffolds are widely associated with bioactivity and play a crucial role in the development of therapeutics, including antiprotozoals,^2^ anticoagulants,^3^ antibacterials,^4^ antivirals,^5^ anti-inflammatories,^6^ antifungals,^7^ and antineoplastics.^8^

Pyrazole is a five-membered aromatic heterocycle containing two adjacent nitrogen atoms at the 1,2-positions, contributing to a planar conjugated π-system with six delocalized electrons^9^. Its significance has stimulated extensive research into synthetic routes, with Knorr’s protocol being among the most widely used.^10,11^ Pyrazole derivatives display a broad spectrum of pharmacological activities, and notable drugs containing this motif include, apixaban, celecoxib, and crizotinib.^12,13^

Beyond approved agents, pyrazole derivatives have been reported to possess a wide range of biological activities, such as anticoagulant,^14^ antibacterial,^15^ antifungal,^15^ anti-inflammatory,^16^ antiprotozoal,^17^ antineoplastic,^18^ and antiviral,^19^ highlighting their versatility in drug discovery.

Thiazole, another five-membered aromatic heterocycle, contains sulfur and nitrogen atoms at the 1- and 3-positions, respectively. Among several methodologies, the Hantzsch synthesis remains a cornerstone for its preparation.^20,21^ Thiazole units are also found in natural products such as thiamine (vitamin B1) and penicillin,^22^ and they form part of the structural cores of clinically relevant agents including dasatinib, meloxicam, ritonavir, and sulfathiazole.^23,24^

Pharmaceutical interest in the thiazole nucleus derives from its capacity to impart diverse pharmacological effects, with reported activities ranging from antituberculosis,^25^ anticancer,^26^ antihypertensive,^27^ and anticonvulsant^28^ to antifungal^29^ and antiprotozoal.^30^ Such diversity has positioned thiazole among the most frequently exploited heterocyclic motifs in medicinal chemistry.

Our research group has previously developed a series of pyrazole-based hybrids, including pyrazole–imidazoline,^31–33^ pyrazole–thiazoline,^34^ pyrazole–tetrahydropyrimidine,^31,35^ pyrazole–thiadiazole,^36^ and pyrazole-benzoimidazole.^37^ These compounds have been evaluated for their efficacy against *T. cruzi*, the etiological agent of Chagas disease (CD), which remains a significant neglected disease prevalent in 21 Latin American countries.^38^

CD treatment relies on benznidazole or nifurtimox, both of which often cause severe adverse effects and exhibit limited efficacy in the chronic phase of the infection.^39,40^ This scenario underscores the urgent need for the development of novel therapeutic agents that are both effective and safe for the treatment of CD. Likewise, tuberculosis, caused by *M. tuberculosis* , persists as the leading cause of death from a single infectious agent worldwide, with rifampicin and isoniazid resistance compromising current regimens.^41–43^ Despite their distinct etiologies, *T. cruzi* and *M. tuberculosis* share several biological and pharmacological challenges, including intracellular persistence, slow growth rates, and the presence of complex, lipid-rich cell envelopes that restrict compound permeability. These shared constraints justify the exploratory evaluation of small-molecule scaffolds across both pathogens as a phenotypic screening strategy, rather than as an attempt to target conserved molecular mechanisms. In this context, CYP51 and cysteine proteases are considered structural reference points based on historical precedent, not as validated or confirmed targets in the present study.^44,45^

In this context, the pyrazole–thiazole hybrid was rationally designed as a promising scaffold against intracellular infections (*T. cruzi* and *M. tuberculosis* ). The selection was based on the molecule’s optimized physicochemical profile, which is fundamental for permeating host and pathogen membranes. Structurally, the hybrid leverages the known activity of its azole components against conserved targets: the pyrazole core has proven inhibitory activity against cruzain^31,46^ and CYP51,^47^ and thiazole derivatives also show potential to modulate cysteine protease activity, as indicated by molecular docking studies.^48^ The fusion of these units aims to improve bioactivity across multiple targets, providing a focused strategy for neglected disease drug discovery.

This work describes the design, synthesis, and characterization of 11 novel pyrazole–thiazole hybrids (**1a–k**). Their activity was assessed against *T. cruzi* and *M. tuberculosis* , complemented by molecular docking studies on CYP51 and cysteine proteases to rationalize activity. The results aim to validate halogenated pyrazole–thiazole hybrids as promising scaffolds for early stage development of agents targeting neglected diseases.

## 2 Materials and Methods

### 2.1 Chemistry

All reagents were commercially available and utilized without further purification. The reaction progress was monitored by thin-layer chromatography (TLC) with precoated 60 F254 silica gel plates. Melting points were taken in glass capillary tubes on an Allerbest apparatus and are uncorrected. Fourier Transform Infrared (FT-IR) spectra were recorded on a PerkinElmer Spectrum 100 instrument with an ATR diamond-ZnSe system, resolution 4 cm^–1^, and 16 scans. Nuclear Magnetic Resonance (NMR) analyses were performed on a Bruker Avance (400 or 500 MHz) equipment, at 298 K, in DMSO-*d6* as solvent. Chemical shifts (δ) are reported in parts per million (ppm), relative to tetramethylsilane (TMS), and coupling constants (*J*) in Hertz (Hz). Mass spectra were acquired on a Micromass/Waters ZQ-4000 Spectrometer. The intermediates 1-aryl-1*H*-pyrazole-4-carbonitriles **3**(**a–k**) and 1-aryl-1*H*-pyrazole-4-carbothioamides **4**(**a–k**) were prepared according to the previously described methods.^49,50^

### General Procedure for the Synthesis of 2-(1-Aryl-1*H*-pyrazol-4-yl)-4-phenylthiazoles **1**(**a**–**k**)

In a 10 mL round-bottom flask, adapted with an Allihn condenser, at room temperature, the intermediates **4**(**a–k**) (1 mmol) were dissolved in *N,N*-dimethylformamide (DMF) (2 mL). Subsequently, bromoacetophenone (1 mmol) was added to the flask and the reaction was heated at 70 °C for 1 to 4 h.^51^ The reactions were accomplished by thin-layer chromatography (TLC) using heptane/ethyl acetate (1:1) as eluent. The reaction mixtures were dissolved in ethanol and poured into cold water. The precipitate was filtered out and recrystallized from a mixture of water and ethanol.

#### 2-(1-(3-Chlorophenyl)-1*H*-pyrazol-4-yl)-4-phenylthiazole (**1a**)

Yield: 27%; Color: light yellow; m.p.: 133–135 °C; FT-IR (cm^–1^): 3137, 3111, 3038, 1596, 1580, 1499, 1479; ^1^H NMR (500 MHz, DMSO-*d*_*6*_) δ 9.31 (s, 1H), 8.35 (s, 1H), 8,11 (t, *J* = 1.5 Hz, 1H), 8.09 (s, 1H), 8,04 (d, *J* = 7.7 Hz, 2H), 7.98 (dd, *J* = 8.2, 1.5 Hz, 1H), 7.58 (t, *J* = 8.2 Hz, 1H), 7.48 (t, *J* = 7.7 Hz, 2H), 7.44 (dd, *J* = 8.2, 1.5 Hz, 1H), 7.38 (t, *J* = 7.7 Hz, 1H); ^13^C NMR (125 MHz, DMSO-*d*_*6*_) δ 158.7, 154.4, 140.1, 139.5, 134.0, 133.8, 131.2, 128.6, 128.0, 126.9, 126.5, 126.0, 119.3, 118.2, 117.0, 113.2; HRMS (ESI) *m*/*z* [M + Na]^+^ = 360.0336 (found), [M + Na]^+^ = 360.0338 (calculated).

#### 2-(1-(2,4-Dichlorophenyl)-1*H*-pyrazol-4-yl)-4-phenylthiazole (**1b**)

Yield: 33%; Color: yellow; m.p.: 138–140 °C; FT-IR (cm^–1^): 3120, 3062, 1596, 1571, 1502, 1480; ^1^H NMR (400 MHz, DMSO-*d*_*6*_) δ 8.83 (s, 1H), 8.34 (s, 1H), 8.08 (s, 1H), 8.03 (d, *J* = 7.3 Hz, 2H), 7.95 (d, *J* = 2.3 Hz, 1H), 7.74 (d, *J* = 8.6 Hz, 1H), 7.65 (dd, *J* = 8.6, 2.3 Hz, 1H), 7.47 (t, *J* = 7.3 Hz, 2H), 7.37 (t, *J* = 7.3 Hz, 1H); ^13^C NMR (100 MHz, DMSO-*d*_*6*_) δ 159.4, 155.0, 139.6, 136.7, 134.5, 134.3, 131.5, 130.5, 130.0, 129.9, 129.2, 128.9, 128.6, 126.5, 118.8, 113.6; HRMS (ESI) *m*/*z* [M + Na]^+^ = 393.9960 (found), [M + Na]^+^ = 393.9948 (calculated).

#### 2-(1-(3,5-Dichlorophenyl)-1*H*-pyrazol-4-yl)-4-phenylthiazole (**1c**)

Yield: 74%; Color: beige; m.p.: 212–214 °C; FT-IR (cm^–1^): 3144, 3087, 1592, 1575, 1493, 1474; ^1^H NMR (400 MHz, DMSO-*d*_*6*_) δ 9.37 (s, 1H), 8.38 (s, 1H), 8.10 (s, 1H), 8.09 (s, 2H), 8.05 (d, *J* = 7.6 Hz, 2H), 7.61 (t, *J* = 1.8 Hz, 1H), 7.48 (t, *J* = 7.6 Hz, 2H), 7.38 (t, *J* = 7.6 Hz, 1H); ^13^C NMR (100 MHz, DMSO-*d*_*6*_) δ 159.0, 155.0, 141.2, 140.4, 135.6, 134.3, 129.2, 128.6, 127.8, 126.6, 126.5, 120.2, 117.6, 113.9; HRMS (ESI) *m*/*z* [M + Na]^+^ = 393.9958 (found), [M + Na]^+^ = 393.9948 (calculated).

#### 2-(1-(3,4-Dichlorophenyl)-1*H*-pyrazol-4-yl)-4-phenylthiazole (**1d**)

Yield: 91%; Color: beige; m.p.: 154–156 °C; FT-IR (cm^–1^): 3126, 3116, 3094, 1589, 1493, 1475; ^1^H NMR (400 MHz, DMSO-*d*_*6*_) δ 9.33 (s, 1H), 8.37 (s, 1H), 8.31 (d, *J* = 2.6 Hz, 1H), 8.09 (s, 1H), 8.04 (d, *J* = 7.6 Hz, 2H), 8.00 (dd, *J* = 8.8, 2.6 Hz, 1H), 7.81 (d, *J* = 8.8 Hz, 1H), 7.48 (t, *J* = 7.6 Hz, 2H), 7.38 (t, *J* = 7.6 Hz, 1H); ^13^C NMR (100 MHz, DMSO-*d*_*6*_) δ 159.1, 155.1, 140.2, 139.2, 134.3, 132.6, 131.9, 129.4, 129.2, 128.6, 127.6, 126.6, 120.6, 120.0, 119.1, 113.8; HRMS (ESI) *m*/*z* [M + Na]^+^ = 393.9942 (found), [M + Na]^+^ = 393.9948 (calculated).

#### 2-(1-(4-Chlorophenyl)-1*H*-pyrazol-4-yl)-4-phenylthiazole (**1e**)

Yield: 65%; Color: light brown; m.p.: 186–188 °C; FT-IR (cm^–1^): 3123, 3067, 3026, 1602, 1504, 1479; ^1^H NMR (400 MHz, DMSO-*d*_*6*_) δ 9.24 (s, 1H), 8.34 (s, 1H), 8.08 (s, 1H), 8.04 (d, *J* = 7.4 Hz, 2H), 8.01 (d, *J* = 8.8 Hz, 2H), 7.62 (d, *J* = 8.8 Hz, 2H), 7.48 (t, *J* = 7.4 Hz, 2H), 7.38 (t, *J* = 7.4 Hz, 1H); ^13^C NMR (100 MHz, DMSO-*d*_*6*_) δ 158.8, 154.4, 139.3, 137.8, 133.8, 130.9, 129.4, 128.6, 128.0, 126.6, 126.0, 120.2, 119.2, 113.1; HRMS (ESI) *m*/*z* [M + Na]^+^ = 360.0341 (found), [M + Na]^+^ = 360.0338 (calculated).

#### 2-(1-(4-Fluorophenyl)-1*H*-pyrazol-4-yl)-4-phenylthiazole (**1f**)

Yield: 60%; Color: yellow; m.p.: 194–196 °C; FT-IR (cm^–1^): 3118, 3073, 3028, 1585, 1514, 1484; ^1^H NMR (400 MHz, DMSO-*d*_*6*_) δ 9.18 (s, 1H), 8.32 (s, 1H), 8.08 (s, 1H), 8.06–7.99 (m, 4H), 7.48 (t, *J* = 7.6 Hz, 2H), 7.43–7.38 (m, 3H); ^13^C NMR (100 MHz, DMSO-*d*_*6*_) δ 161.1 (d, *J* = 242.0 Hz), 159.5, 155.0, 139.6, 136.2, 134.4, 129.2, 128.6, 127.2, 126.6, 121.2 (d, *J* = 8.5 Hz), 119.5, 116.8 (d, *J* = 22.9 Hz), 113.6; HRMS (ESI) *m*/*z* [M + Na]^+^ = 344.0632 (found), [M + Na]^+^ = 344.0634 (calculated).

#### 2-(1-(3-Fluorophenyl)-1*H*-pyrazol-4-yl)-4-phenylthiazole (**1g**)

Yield: 33%; Color: light brown; m.p.: 141–143 °C; FT-IR (cm^–1^): 3122, 3064, 3029, 1612, 1586, 1504, 1477; ^1^H NMR (400 MHz, DMSO-*d*_*6*_) δ 9.28 (s, 1H), 8.35 (s, 1H), 8.09 (s, 1H), 8,05 (d, *J* = 7.6 Hz, 2H), 7.89–7.85 (m, 2H), 7.63–7.57 (m, 1H), 7.48 (t, *J* = 7.6 Hz, 2H), 7.38 (t, *J* = 7.6 Hz, 1H), 7.22 (td, *J* = 8.7, 2.2 Hz, 1H); ^13^C NMR (100 MHz, DMSO-*d*_*6*_) δ 163.0 (d, *J* = 242.2 Hz), 159.3, 155.0, 141.0 (d, *J* = 10.5 Hz), 140.0, 134.3, 131.9 (d, *J* = 9.0 Hz), 129.2, 128.6, 127.4, 126.6, 119.8, 115.0, 114.0 (d, *J* = 21.0 Hz), 113.8, 106.4 (d, *J* = 26.7 Hz); HRMS (ESI) *m*/*z* [M + Na]^+^ = 344.0622 (found), [M + Na]^+^ = 344.0643 (calculated).

#### 2-(1-(4-Bromophenyl)-1*H*-pyrazol-4-yl)-4-phenylthiazole (**1h**)

Yield: 68%; Color: light brown; m.p.: 156–158 °C; FT-IR (cm^–1^): 3121, 3066, 3027, 1595, 1499, 1479; ^1^H NMR (400 MHz, DMSO-*d*_*6*_) δ 9.24 (s, 1H), 8.34 (s, 1H), 8.08 (s, 1H), 8.04 (d, *J* = 7.3 Hz, 2H), 7.95 (d, *J* = 8.9 Hz, 2H), 7.75 (d, *J* = 8.9 Hz, 2H), 7.48 (t, *J* = 7.3 Hz, 2H), 7.38 (t, *J* = 7.3 Hz, 1H); ^13^C NMR (100 MHz, DMSO-*d*_*6*_) δ 159.3, 155.0, 139.9, 138.8, 134.3, 132.9, 129.2, 128.5, 127.2, 126.5, 121.0, 119.8, 119.7, 113.7; HRMS (ESI) *m*/*z* [M + Na]^+^ = 403.9821 (found), [M + Na]^+^ = 403.9833 (calculated).

#### 2-(1-(3-Bromophenyl)-1*H*-pyrazol-4-yl)-4-phenylthiazole (**1i**)

Yield: 28%; Color: light brown; m.p.: 139–140 °C; FT-IR (cm^–1^): 3114, 3066, 3023, 1590, 1576, 1497, 1478; ^1^H NMR (400 MHz, DMSO-*d*_*6*_) δ 9.31 (s, 1H), 8.35 (s, 1H), 8.24 (t, *J* = 1.9 Hz, 1H), 8.09 (s, 1H), 8.05 (d, *J* = 7.2 Hz, 2H), 8.02 (dd, *J* = 8.1, 1.0 Hz, 1H), 7.59–7.56 (m, 1H), 7.52–7.46 (m, 3H), 7.38 (t, *J* = 7.1 Hz, 1H); ^13^C NMR (100 MHz, DMSO-*d*_*6*_) δ 159.3, 155.0, 140.7, 140.0, 134.3, 132.0, 130.0, 129.2, 128.6, 127.4, 126.6, 122.8, 121.5, 119.8, 118.0, 113.7; HRMS (ESI) *m*/*z* [M + Na]^+^ = 403.9836 (found), [M + Na]^+^ = 403.9833 (calculated).

#### 2-(1-(4-Methoxyphenyl)-1*H*-pyrazol-4-yl)-4-phenylthiazole (**1j**)

Yield: 84%; Color: white; m.p.: 185–187 °C; FT-IR (cm^–1^): 3122, 3000, 2950, 2934, 2832, 1601, 1577, 1518, 1483; ^1^H NMR (400 MHz, DMSO-*d*_*6*_) δ 9.08 (s, 1H), 8.26 (s, 1H), 8.06 (s, 1H), 8.04 (d, *J* = 7.6 Hz, 2H), 7.87 (d, *J* = 9.0 Hz, 2H), 7.48 (t, *J* = 7.6 Hz, 2H), 7.37 (t, *J* = 7.6 Hz, 1H), 7.10 (d, *J* = 9.0 Hz, 2H), 3.82 (s, 3H); ^13^C NMR (100 MHz, DMSO-*d*_*6*_) δ 159.7, 158.5, 154.9, 139.0, 134.4, 133.2, 129.2, 128.5, 126.7, 126.6, 120.7, 119.1, 115.1, 113.4, 55.9; HRMS (ESI) *m*/*z* [M + Na]^+^ = 356.0825 (found), [M + Na]^+^ = 356.0834 (calculated).

#### 2-(1-(2,3-Dichlorophenyl)-1*H*-pyrazol-4-yl)-4-phenylthiazole (**1k**)

Yield: 38%; Color: light brown; m.p.: 147–148 °C; FT-IR (cm^–1^): 3128, 3077, 3031, 1596, 1582, 1495, 1475; ^1^H NMR (400 MHz, DMSO-*d*_*6*_) δ 8.86 (s, 1H), 8.35 (s, 1H), 8.07 (s, 1H), 8.03 (d, *J* = 8.0 Hz, 2H), 7.85 (d, *J* = 8.1 Hz, 1H), 7.70 (d, *J* = 8.0 Hz, 1H), 7.58 (t, *J* = 8.1 Hz, 1H), 7.48 (t, *J* = 7.7 Hz, 2H), 7.38 (t, *J* = 7.4 Hz, 1H); ^13^C NMR (100 MHz, DMSO-*d*_*6*_) δ 158.8, 154.4, 139.0, 138.9, 133.8, 132.7, 131.1, 130.9, 128.7, 128.6, 128.0, 127.5, 127.1, 126.0, 118.2, 113.1; HRMS (ESI) *m*/*z* [M + Na]^+^ = 393.9935 (found), [M + Na]^+^ = 393.9948 (calculated).

### 2.3 *In Silico* Prediction/Computational Analysis

The physicochemical properties of the pyrazole-thiazole derivatives were evaluated, encompassing total molecular weight (MW), lipophilicity (cLogP), solubility in water, pH 7.4 (cLogS), number of hydrogen bond donors (HBD) and acceptors (HBA), topological polar surface area (tPSA), drug-likeness metrics, the count of rotational bonds, and the number of sp3 atoms. These parameters were determined using DataWarrior software (version 5.5.0).^52^

### 2.4 Anti-Trypanosomal Studies

#### 2.4.1 Cell and Parasite Culture

Vero cells, sourced from the Rio de Janeiro Cell Bank (BCRJ code 0245), were cultured in RPMI-1640 medium enriched with 10% Fetal Bovine Serum (FBS), 1 mM l-glutamine, and antibiotics, under standard culture conditions at 37 °C in a 5% CO_2_ atmosphere. These cells were used to culture parasites and perform phenotypic screening assays. The genetically modified *T. cruzi* expressing luciferase, Dm28c-Luc, was supplied by Dr. Cristina Henriques of the Oswaldo Cruz Institute–Fiocruz.^53^ The parasites were cultured at a 10:1 ratio with Vero cells in the culture medium. After a four-day infection period, the trypomastigotes were harvested from the supernatant for subsequent trypanocidal activity assays.

The murine macrophage cell line RAW 264.7 (TIB-71) was obtained from the American Type Culture Collection (ATCC, Manassas, VA, USA). Cells were cultured in Dulbecco’s Modified Eagle Medium (DMEM, Gibco, Thermo Fisher Scientific) supplemented with 10% (v/v) heat-inactivated fetal bovine serum (FBS), 1% penicillin-streptomycin (100 U/mL and 100 μg/mL, respectively), and 2 mM l-glutamine. Cultures were maintained at 37 °C in a humidified atmosphere with 5% CO_2_, and subcultured every 2–3 days. All experiments were performed using cells between passages 3 and 7 to ensure phenotypic stability and reproducibility.

#### 2.4.2 Cytotoxicity Assays

Vero cells were seeded at 1.5 × 10^4^ cells per well in 96-well-white-bottomed plates and cultured for 24 h. Following this incubation period, the cells were exposed to pyrazole-thiazole derivatives and benznidazole (Bz) as a positive control for 72 h, employing a 1:2 serial dilution method that yielded concentrations ranging from 500 μM to 15.62 μM. Dimethyl sulfoxide (DMSO) was used as a negative control at a final concentration of less than 1%. Cell viability was assessed by quantifying ATP levels using the CellTiter Glo Kit solution (Promega, Madison, USA). Luminescence measurements were obtained using the Glomax Microplate Reader (Promega Corporation, Madison, WI, USA). The CC_50_ values, representing the concentrations of the compound that reduce cell viability by 50%, were calculated using GraphPad Prism software.^54^ All experiments were performed in triplicate, with each trial performed in duplicate.

For RAW264.7 macrophages cytotoxicity assessment, cells were seeded in 96-well flat-bottom microplates at a density of 1 × 10^4^ cells per well in 100 μL of complete medium and incubated overnight to allow adhesion. After 24 h, the medium was replaced with fresh DMEM containing the test compounds at serial concentrations ranging from 0.5 to 500 μM. Untreated cells were used as the negative control (100% viability), and 1% Triton X-100 served as the positive control for complete cell lysis. Plates were incubated for 24 h under standard conditions. Cytotoxicity was quantified using the resazurin reduction assay. Briefly, 20 μL of resazurin solution (0.01% w/v in PBS) was added to each well, followed by incubation for 4 h at 37 °C. The metabolic conversion of resazurin to resorufin was measured by fluorescence at λ ex = 530 nm and λ em = 590 nm using a microplate reader (SpectraMax M3, Molecular Devices).

#### 2.4.3 Anti-*T. cruzi* Activity

The phenotypic screening of pyrazole-thiazole derivatives was performed using the trypomastigote and intracellular amastigote forms of *T. cruzi*.^36^ Trypomastigote forms of *T. cruzi* Dm28c-Luc (1 × 10^6^ parasites/well) were added to 96-well white-bottom plates and treated with the derivatives at 37 °C with serial dilutions (1:3) at varying concentrations (100–0.41 μM). After 24 h, 300 μg/mL luciferin, the luciferase substrate, was added to determine the viability of the parasite by enzyme activity and the luminescent reading performed in Glomax Microplate Reader (Promega Corporation, Madison, WI, USA). For intracellular amastigotes, Vero cells seeded in a 96-well white-bottom plate (1.5 × 10^4^) were infected with *T. cruzi* Dm28c-Luc (10:1 ratio). After 24 h of interaction, the cultures were treated with pyrazole-thiazole derivatives (100–0.41 μM) for 72 h at 37 °C. After treatment, luciferin (300 μg/mL) was added, and parasite viability was measured in a Glomax reader. DMSO (<1%) was used as the negative control and Bz (100–0.41 μM) as the positive control. The concentration that inhibits 50% (IC_50_) or 90% (IC_90_) of parasite viability was determined using GraphPad Prism software (version 8.2.1). The Selectivity Index (SI) was calculated as the CC_50_/IC_50_ ratio. Three independent assays were performed in duplicate.

### 2.5 Anti-Mycobacterial Studies

#### 2.5.1 Mycobacterial Strains

The antimycobacterial activity against *M. tuberculosis* H37Rv (ATCC 27294) was evaluated using long-phase cultures grown in Middlebrook 7H9 broth supplemented with oleic acid, bovine serum albumin fraction V, dextrose, and catalase (OADC). Strains were thawed and cultured under agitation (200 rpm) at 37 °C for 3 weeks until log-phase growth was achieved.

#### 2.5.2 Anti-Mycobacterial Activity

Antimycobacterial activity was determined using the Resazurin Microtiter Assay (REMA). Briefly, cultures were grown in 7H9-OADC medium for 30 days, adjusted to a turbidity equivalent to a 1.0 McFarland standard (∼3 × 10^8^ CFU/mL), and subsequently diluted for use in MIC testing. Serial 2-fold dilutions of the samples and reference drug (64–0.004 μM) were prepared in 96-well microplates and inoculated with bacterial suspensions. Plates were incubated at 37 °C under 5% CO_2_ for 7 days. Following incubation, resazurin solution was added, and fluorescence was recorded after 24 h using a Synergy H1 microplate reader (BioTek, Winooski, VT, USA) at excitation/emission wavelengths of 530/590 nm. All experiments were performed in triplicate, and MIC values were defined as the lowest concentration preventing detectable metabolic activity.^55^

### 2.6 *In Silico* Studies and Molecular Analysis

#### 2.6.1 Receptors Used for the Molecular Docking for *T. cruzi*

Molecular docking analysis was carried out to the most active compounds against *T. cruzi*, using two well-characterized receptors with available crystal structures in the PDB. The first was Cruzain, a cathepsin L-like cysteine protease essential for parasite invasion and replication and widely validated as a pharmacological target. The second was sterol 14-α-demethylase (CYP51), a key enzyme in the trypanosomatids redox system that lacks a functional human homologue, thereby representing a highly selective target.

The optimized 3D structure of the ligand was generated with MolView. The structure of Cruzain (PDB ID: 1EWP) was retrieved, and residues with alternate conformations were removed to obtain a clean file suitable for HADDOCK. Docking was then performed, and the best model was selected according to the HADDOCK score. Binding affinity was subsequently estimated using PRODIGY, which applies a linear regression model based on interfacial contacts.^56^

For CYP51, the structure was obtained from the crystallographic file (PDB ID: 4COH). Because of missing atoms, the sequence was refined using AlphaFold 3 to generate a complete model. Docking with **1g** was then carried out considering the following residues as active-site components: I45, F48, I70, I72, Y103, I105, M106, F110, Y116, P210, A211, V213, F214, A287, F290, A291, G292, L356, M358, M360, M460, V461, and the conserved P450 threonine T295. Together, these residues form a predominantly hydrophobic binding pocket.^57^

#### 2.6.2 Receptors Used for the Molecular Docking for *M. tuberculosis*

Given that compound **1h** emerged as the most active analogue, a homology-based search was performed to prospectively identify receptors with functions analogous to those described for *T. cruzi* (Scheme 1). The aim was to determine whether **1h** shares a similar mode of action with its analogue **1g** previously shown to display strong binding affinity to these receptors.

**Scheme 1:**
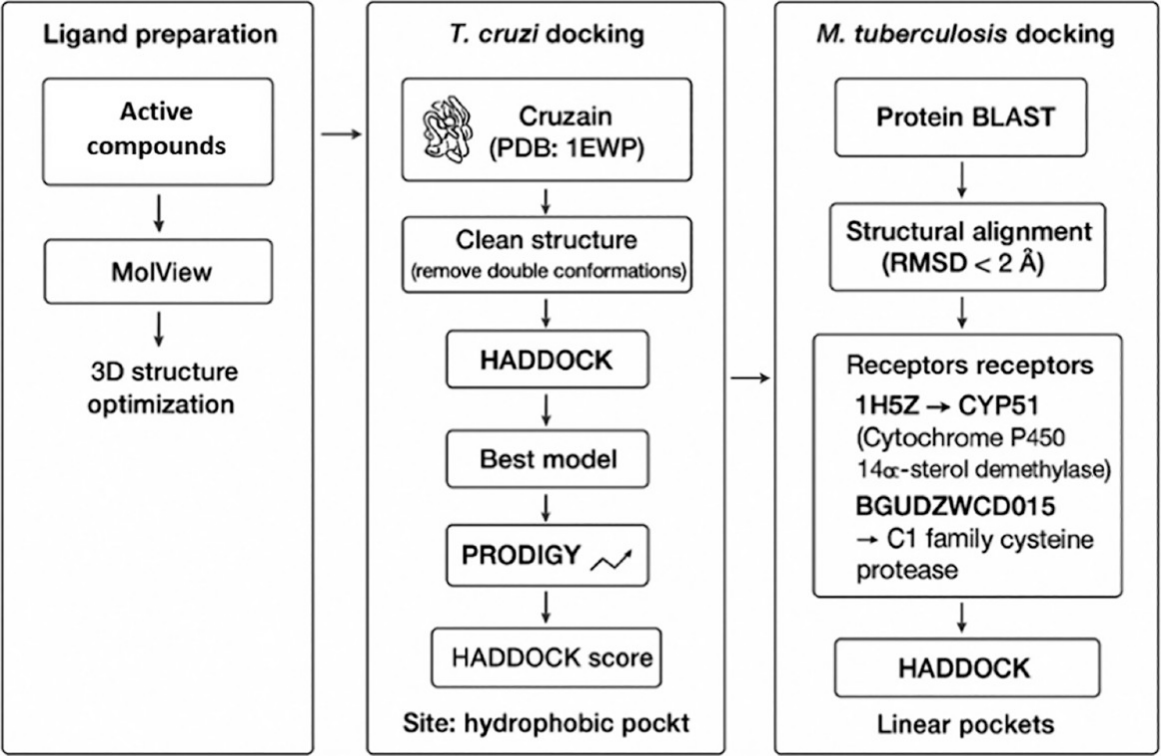
Schematic Outline of the *In Silico* Methodology Applied in This Study

A sequence alignment was carried out using PROTEIN BLAST, and sequences with the highest similarity scores were selected for structural alignment. Only candidates with a root-mean-square deviation (RMSD) < 2 Å were considered, using the BLOSUM-62 matrix and Needleman–Wunsch alignment algorithm. The receptors fulfilling these criteria were Cytochrome P450 14α-sterol demethylase (CYP51, PDB ID: 1H5Z) and a C1 family cysteine protease from *M. tuberculosis* (BGUDZWCD015). These structures were subsequently docked with HADDOCK using the **1g** ligand, maintaining the same active-site residues previously defined for 1EWP and 4COH.

## 3 Results and Discussion

### 3.1 Preparation and Analytical Characterization Results

The synthetic pathway to target the compounds 2-(1-aryl-1*H*-pyrazol-4-yl)-4-phenylthiazoles **1**(**a**–**k**) is exhibited in Scheme 2. The intermediates 1-aryl-1*H*-pyrazole-4-carbonitriles **3**(**a**–**k**) were prepared from 5-amino-1-aryl-1*H*-pyrazole-4-carbonitriles **2**(**a**–**k**), *t*-butyl nitrite in tetrahydrofuran (THF), under reflux for 2 h, through an aprotic deamination reaction. Treatment of **3**(**a**–**k**) with Lawesson′s reagent and ethanol as solvent, for 24 h under reflux, afforded the key intermediates 1-aryl-1*H*-pyrazole-4-carbothioamides **4**(**a**–**k**). In the last step, **4**(**a**–**k**) reacted with bromoacetophenone in dimethylformamide (DMF), for 1–4 h at 70 °C, to lead to the desired compounds **1**(**a**–**k**). The structures of **1**(**a**–**k**) were confirmed by analyses of FT-IR and ^1^H/^13^C NMR spectroscopies, and HRMS. FT-IR spectra showed typical absorption bands related to aromatic rings, at 3144–3000 cm^–1^ and 1612–1474 cm^–1^ attributed to C_sp2_-H and C=C/C=N stretchings, respectively. Additionally, two bands at 2950 and 2832 cm^–1^ were identified due to Cs_p3_-H stretchings of the methoxy group in **1j** derivative. NMR analyses exhibited three singlet signals at 9.37–8.83, 8.38–8.26, and 8.10–8.06 ppm, corresponding to three protons attached to pyrazole and thiazole rings. As expected, the protons attached to both benzene rings generated signals at 8.31–7.10 ppm, in the typical aromatic range. No signals were identified below 7.10 ppm, except for **1j**, which has a singlet signal at 3.82 ppm, attributed to the methoxy group. ^13^C NMR spectra showed signals in aromatic region, ranging from 163.0 to 106.4 ppm. Doublet signals were observed for **1f** and **1g** due to coupling between fluorine and carbon atoms. In addition, for **1h**, the methoxy group signal was identified at 55.9 ppm. HRMS analyses of **1**(**a–k**) showed that the found values were very close to the theoretical values with errors varying from 0.56 to 3.49 ppm.

**Scheme 2:**
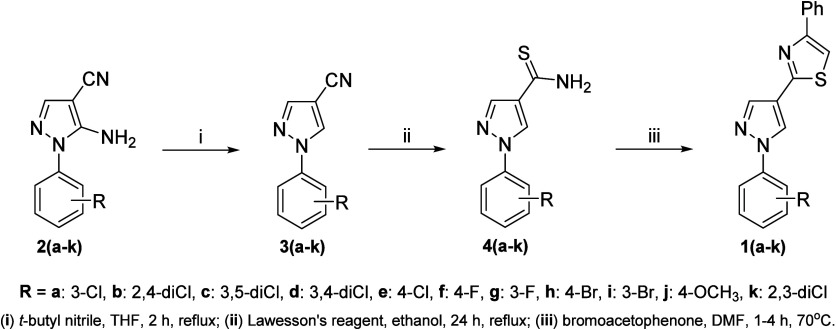
Synthetic Pathway to Furnish -2-(1-aryl-1*H*-pyrazol-4-yl)-4-phenylthiazoles **1**(**a**–**k**)

### 3.2 Evaluation of Computational Physicochemical Parameters

The physicochemical properties of the synthesized pyrazole-thiazole derivatives **1**(**a**–**k**) were assessed, as these parameters critically affect their cellular membrane permeability and, thereby, their biological activity. The 11 compounds studied are small molecules, characterized by molecular weights ranging from 321.38 to 382.28 g/mol (Figure 1). The derivatives exhibited low aqueous solubility, with log S values ranging from −4.19 to −4.92. However, compounds **1f** (−3.77), **1g** (−3.77), and **1j** (−3.47) were predicted to have moderate solubility. Most derivatives displayed moderate lipophilicity, as indicated by cLogP values ranging from 3.16 to 4.44 (Figure 1). This lipophilic profile suggests their potential for enhanced absorption and favorable bioavailability in biological systems. The compounds predominantly exhibited three hydrogen bond acceptors (HBA), except for derivative **1j**, which presented four. No hydrogen bond donors (HBD) were detected in any derivatives. The topological polar surface area (tPSA), a critical determinant of a compound’s membrane permeability, was found to be 58.95 Å^2^ for most derivatives, except for the **1j** derivative, which exhibited a tPSA of 68.18 Å^2^ (Figure 1). The compounds demonstrated predominantly rotatable bond (RB) values of 3 (Figure 1), indicating favorable conformational flexibility, that may enhance their interactions with biological targets. Pyrazole-thiazole derivatives typically adhere to the Lipinski and Veber rule criteria, indicating favorable properties for drug-likeness and oral bioavailability.

**Figure 1:**
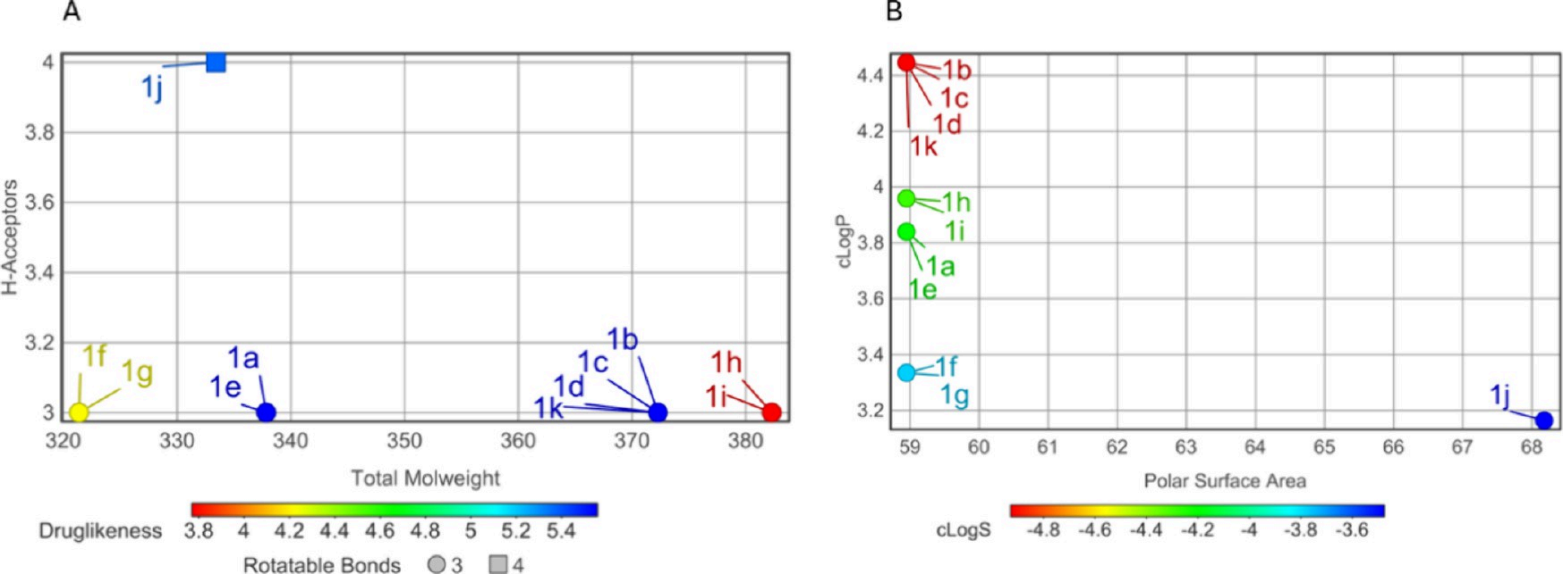
Physicochemical properties of pyrazole-thiazole calculated using DataWarrior software. (A) Molecular weight, hydrogen bond acceptors (H-acceptors), rotatable bonds, and drug-likeness. (B) Polar surface area, lipophilicity (cLogP), and water solubility (cLogS).

### 3.3 Trypanosoma cruzi

#### 3.3.1 Biological Evaluation

Pharmacological properties of substances encompass both their toxicological profile and therapeutic efficacy. In our study, we examined the cytotoxicity of pyrazole-thiazole derivatives in Vero cells and evaluated their efficacy against *T. cruzi* (Table 1). The screening assessed the trypomastigote and intracellular amastigote stages of *T. cruzi* in the mammalian host. The pyrazole-thiazole hybrids demonstrated limited activity against these developmental forms, with most IC_50_ values exceeding 100 μM. Two derivatives, **1g** (3-F) and **1k** (2,3-diCl), displayed activity against the trypomastigote stage. Among the more active compounds, derivative **1g** exhibited an IC_50_ of 11.66 ± 1.38 μM, comparable to the reference drug, Benznidazole (Bz; IC_50_ = 11.32 ± 0.15 μM).^58,59^ In contrast, derivative **1k** exhibited an IC_50_ of 24.58 ± 5.48 μM, indicating a lower efficacy. Regarding intracellular amastigotes, four derivatives, **1a** (3-Cl), **1f** (4-F), **1g** (3-F), and **1i** (3-Br), showed activity with IC_50_ values below 100 μM. The derivatives displayed distinct IC_50_ values: **1a** had an IC_50_ of 91.16 ± 0.47 μM, **1f** had an IC_50_ of 64.59 ± 4.17 μM, **1g** had an IC_50_ of 26.83 ± 0.07 μM, and **1i** had an IC_50_ of 89.12 ± 4.67 μM. The measured values indicate a reduced activity profile relative to Benznidazole (Bz), which exhibits an IC_50_ of 1.94 ± 0.06 μM. Derivative **1g** (3-F) was identified as the only compound displaying potential activity against both forms of *T. cruzi*, with a selectivity index (SI) greater than 10. Although derivative **1g** (3-F) exhibited the most promising profile in this series, its activity against intracellular amastigotes (IC_50_ = 26.83 μM) remains above the potency threshold established by the DNDi criteria, which suggests an IC_50_ < 10 μM for a compound to be considered a hit for further development.^60^ Furthermore, compared with Bz (IC_50_ = 1.94 μM), the potency of the pyrazole-thiazole hybrids remains modest. In this regard, a comprehensive structure–activity relationship (SAR) analysis is essential to identify the structural determinants of potency, thereby providing the necessary support for strategic optimization efforts to achieve clinical benchmarks.

**Table 1:** Toxic Effect and Anti-*T. cruzi* Activity of Pyrazole-Thiazole Derivatives^a^

**compounds**	**intracellular amastigotes**			**trypomastigotes**			**VERO cells**
	IC_50_ (μM)	IC_90_ (μM)	SI	IC_50_ (μM)	IC_90_ (μM)	SI	CC_50_ (μM)
**1a** (3-Cl)	91.16 ± 0.47	>100	>5.45	>100	>100	Nd	>500
**1b** (2,4-diCl)	>100	>100	Nd	>100	>100	Nd	>500
**1c** (3,5-diCl)	>100	>100	Nd	>100	>100	Nd	>500
**1d** (3,4-diCl)	>100	>100	Nd	>100	>100	Nd	>500
**1e** (4-Cl)	>100	>100	Nd	>100	>100	Nd	>500
**1f** (4-F)	64.59 ± 4.17	>100	>7.74	>100	>100	Nd	>500
**1g** (3-F)	26.83 ± 0.07	36.57 ± 3.47	>18.97	11.66 ± 1.38	92.39 ± 2.59	>42.8	>500
**1h** (4-Br)	>100	>100	Nd	>100	>100	Nd	>500
**1i** (3-Br)	89.12 ± 4.67	>100	>5.61	>100	>100	Nd	>500
**1j** (4-OCH_3_)	>100	>100	Nd	>100	>100	Nd	>500
**1k** (2,3-diCl)	>100	>100	Nd	24.58 ± 5.48	>100	>20.34	>500
Bz	1.94 ± 0.06	9.33 ± 0.15	>263.15	11.32 ± 0.15	>100	>44.16	>500

a Mean values for IC_50_ and IC_90_ from three independent experiments ± standard deviation (SD); IC_50_: concentration that inhibits parasite viability by 50%; CC_50_: concentration that reduces the viability of VERO cells by 50%; Nd = Not determined; Selectivity index (SI) = CC_50_ of VERO cells/IC_50_ of trypomastigote and intracellular amastigote forms of *T. cruzi*.

The biological evaluation against intracellular amastigotes showed that the substitution pattern on the phenyl ring is a key determinant of potency, with a strong preference for *meta*-substituted halogens. Derivative **1g** (3-F) was the most potent, suggesting that the small size and high electronegativity of fluorine at 3-position enhance parasite inhibition. This is supported by the fact that the 3-substituted series ranked *F* > Br > Cl, while *para* (4-) and *ortho* (2-) substitutions, as in derivatives **1f** (4-F) and **1e** (4-Cl), resulted in much lower or complete loss of activity. The type of substituent also affected efficacy; halogens were tolerated at specific positions, whereas methoxy (OCH_3_) (**1j**), an electron-donating group (EDG), caused inactivity. Additionally, all disubstituted analogs (**1b**, **1c**, **1d**, and **1k**) failed to inhibit amastigote growth. This suggests that, for this specific chemical series, the combination of increased molecular volume and high lipophilicity (cLogP > 4.0) may be detrimental, potentially hindering proper orientation within the biological target or reducing cellular uptake. Additionally, the improved performance of **1g** may also correlate with its higher predicted solubility (clogS = −3.77) relative to the more lipophilic chloride and bromide derivatives, underscoring that a balance between membrane permeability and solubility is crucial for reaching intracellular parasites.

Recent studies have highlighted the antiprotozoal efficacy of thiazolyl-pyrazoline hybrids, demonstrating significant activity against *Plasmodium falciparum* (EC_50_ = 11.80 μM), *Leishmania (V) panamensis* (EC_50_ = 6.46 μM), and *T. cruzi* (EC_50_ = 4.98 μM).^61^ Notably, pyrazole-thiazoline derivatives showed enhanced potency against *T. cruzi*, with derivative **2c** (2,4-diCl) exhibiting remarkable inhibitory concentrations of 0.4 ± 0.02 μM and 1.4 ± 0.4 μM against the trypomastigotes and intracellular amastigotes, respectively.^34^

#### 3.3.2 Molecular Docking and Affinity Prediction

Although compound **1g** did not display sufficient intracellular amastigote activity to justify a mechanistic interpretation, we still performed a preliminary docking analysis to extract structural information that may guide future optimization. The selection of cruzain and CYP51 was based on the historical use of pyrazole and pyrazolo-containing scaffolds against these targets.^31,47^ It is important to note, however, that CYP51 has lost clinical momentum after the failure of posaconazole in patients, and is no longer considered a leading target for CD. Therefore, the docking data below must be interpreted strictly as exploratory, and not as evidence of mechanism. Molecular docking analysis between molecule **1g** and the cathepsin L-like protease (cruzain) revealed a theoretical binding affinity of −8.73 kcal/mol, corresponding to a submicromolar dissociation constant of approximately 0.4 μM. This value is within a range considered promising for initial compounds in inhibitor design, suggesting substantial potential for the ligand to interact with the enzymatic environment, which is qualitatively consistent with the *in vitro* IC_50_ results. Furthermore, the interaction profile shows that ligand binding is dominated mainly by hydrophobic contacts, highlighting the interactions with Met68, Ala138 and, to a lesser extent, with the aliphatic side chain of Glu208. These contacts reinforce the hypothesis that the molecule is stably accommodated within the enzyme’s hydrophobic pocket, which is critical for substrate recognition in cathepsin/cruzain-type proteases.^62,63^ Of particular relevance is the discovery of a hydrogen bond between Met68 and an aromatic nitrogen atom of the ligand, with a favorable geometric orientation (angle of 166.4°).

Although this is a point interaction, this bond contributes significantly to the specificity and stability of the complex (Figure 2). Met68’s dual participation in both hydrophobic interactions and hydrogen bonding positions it as a key residue in ligand stabilization. However, no direct interactions with the catalytic dyad (Cys25–His162) are observed, suggesting that the molecule likely acts as a noncovalent inhibitor located in adjacent regions (S2/S3) of the active site. This arrangement would explain the marked dependence on apolar contacts and the limited network of polar interactions observed. Taken together, these docking results indicate that ligand **1g** exhibits a hydrophobically driven binding mode with point-like hydrogen bonding, supporting the predicted affinity in the submicromolar range.^64,65^

**Figure 2:**
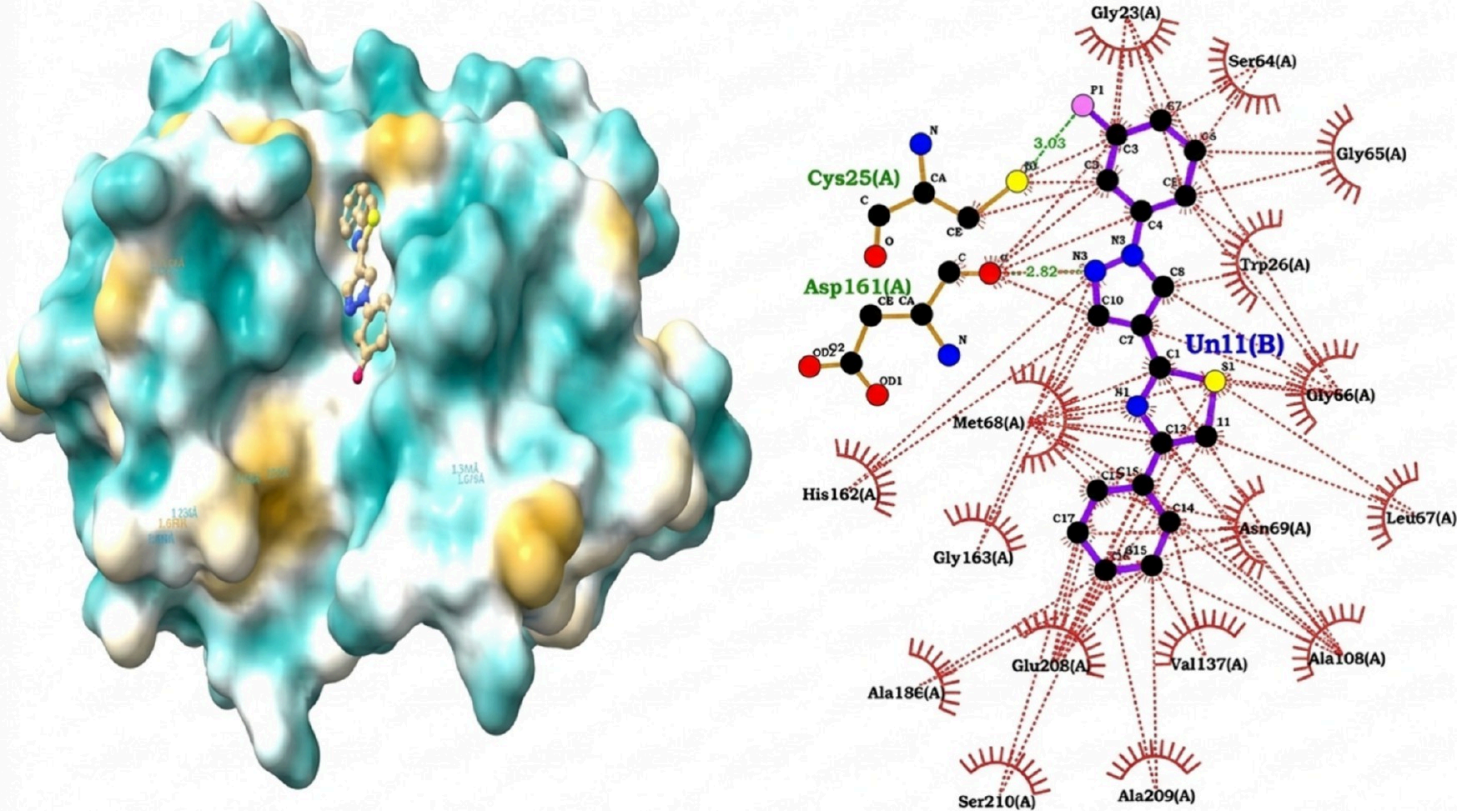
Predicted binding mode of ligand **1g** with cruzain. Left: surface representation of the enzyme showing ligand accommodation in the hydrophobic pocket. Right: 2D interaction diagram highlighting hydrophobic contacts (red arcs) and hydrogen bonds (green dashed lines). Catalytic residues (Cys25 and His162) are shown, although no direct interactions were detected, suggesting a noncovalent binding mode in adjacent subsites (S2/S3).

The **1g**–CYP51 complex showed a predicted binding free energy of −8.76 kcal/mol, in the same submicromolar range observed for cruzain, indicating a comparable recognition potential (Figure 3). The contact profile was dominated by hydrophobic interactions, consistent with the lipophilic access channel of CYP51.^57,66^ Quantitatively, carbon–carbon contacts (CC, 55.8%) prevailed, followed by carbon–nitrogen (CN, 19.4%) and carbon–oxygen (CO, 10.7%) interactions.

**Figure 3:**
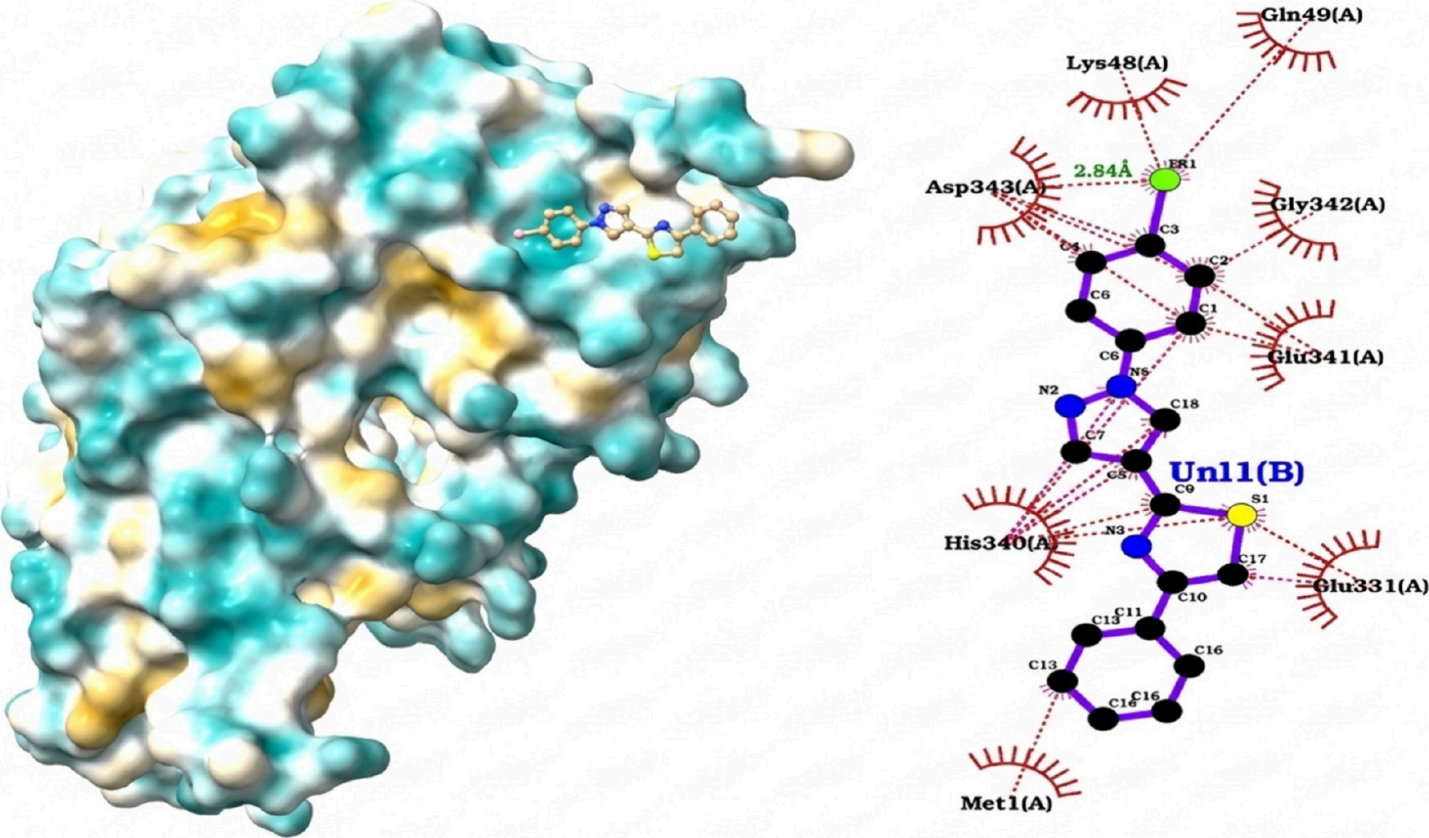
Predicted binding mode of ligand **1g** with CYP51. Left: surface representation of the enzyme showing ligand accommodation in the hydrophobic channel. Right: 2D interaction diagram highlighting dominant apolar contacts (red arcs), a key apolar interaction with Asp343, and π–cation interactions with His340 that stabilize the complex in the absence of hydrogen bonds.

At the residue level, Asp343 contributed through apolar contacts involving its methylene segments (3.93 Å), while no electrostatic interactions were detected. Of particular importance were two π–cation interactions with His340 (distances 4.29 and 4.37 Å; offsets 1.85–2.00 Å), in which an aromatic ring of the ligand stabilized the complex against protonated imidazole. Together, these interactions compensate for the absence of hydrogen bonds and support the overall affinity.

This distinct mode of recognition suggests that the aromatic core and hydrophobic surface of ligand **1g** are central for CYP51 stabilization, with π–cation interactions at His340 reinforcing binding. Collectively, these docking data suggest that **1g** may behave as a submicromolar binder to both cruzain and CYP51, albeit through distinct binding strategies. Thus, **1g** should be considered as a structural starting point (scaffold) for future design and testing of inhibitors targeting homologous receptors in *M. tuberculosis*. These observations should be interpreted strictly as computational hypotheses, requiring experimental validation through enzymatic or biophysical assays.

### 3.4 Mycobacterium tuberculosis

#### 3.4.1 Antimycobacterial Activity

Among the series of pyrazole–thiazole hybrids synthesized, only derivative **1h**, (4-Br), demonstrated significant antimycobacterial activity against *M. tuberculosis* H37Rv (Table 2). The compound exhibited a MIC value of 32.28 ± 0.05 μM, while all other analogues tested (**1b**, **1c**, **1d**, **1e**, **1f**, **1g**, **1j**, and **1k**) showed MIC values above 64 μM, indicating negligible activity. Compound **1h** also displayed favorable *in silico* interaction profiles, supporting its prioritization for further investigation.

**Table 2:** Antimycobacterial Activity (MIC) against *M. tuberculosis* and Cytotoxicity (CC_50_) in RAW Cells of the Synthesized Compounds

sample	H37Rv (μM)	RAW264.7 (μM)
1b	>64	>500
1c	>64	>500
1d	>64	>500
1e	>64	>500
1f	>64	>500
1g	>64	>500
1h	32.28 ± 0.05	>500
1j	>64	>500
1k	>64	>500
Rifampicin	0.005	

The introduction of a 4-bromophenyl substituent at the pyrazole moiety (compound **1h**) markedly improved antimycobacterial activity compared to the other structural variants. This result highlights the role of halogen substitution, particularly bromine, in enhancing biological potency, likely through increased lipophilicity and stronger hydrophobic interactions with the bacterial target site. The favorable *in silico* interaction profile of **1h** may help rationalize with its growth inhibitory effect, suggesting that the compound may engage relevant intracellular pathways in *M. tuberculosis* . The lack of activity in the remaining derivatives underscores the structural sensitivity of this chemotype, where subtle changes in substitution pattern can abolish biological efficacy. The distinct behavior of **1h** suggests that the synergistic contribution of the bromophenyl group with the thiazole–pyrazole core creates an optimal pharmacophoric arrangement for antimycobacterial action.

The absence of cytotoxic effects in RAW 264.7 macrophages across the compound series suggests that the pyrazole–thiazole framework is not intrinsically associated with host cell damage under the conditions tested. Evaluation of host cell compatibility alongside antimycobacterial activity is a standard early stage criterion in tuberculosis drug discovery, particularly when macrophage-based systems are involved.^67^ This is particularly relevant considering that macrophages constitute the primary niche for *M. tuberculosis* infection. Therefore, any compound progressing in the antitubercular pipeline must demonstrate compatibility with macrophage viability.

For compound **1h**, the separation between its inhibitory concentration against *M. tuberculosis* and the upper range of macrophage tolerance indicates that its antimycobacterial activity is unlikely to arise from general membrane disruption or nonspecific toxicity. Instead, the data support a more selective bacterial growth inhibition profile. Although the potency remains moderate compared to rifampicin, the preserved macrophage viability provides a rational basis for further optimization, particularly aimed at improving target engagement without compromising cellular compatibility. In contrast, the inactive derivatives, despite being nontoxic, failed to inhibit bacterial growth, reinforcing the notion that minor structural variations within this chemotype critically influence antibacterial efficacy while not necessarily affecting host cell tolerance. This divergence between bacterial activity and macrophage safety highlights the structural specificity required to balance potency and selectivity in this series.

Overall, the data positions compound **1h** as a promising lead molecule for further optimization. Its moderate MIC, favorable binding profile, and straightforward synthesis support continued exploration of brominated pyrazole–thiazole derivatives, with potential for derivatization aimed at potency enhancement, pharmacokinetic improvement, and *in vivo* validation against resistant *M. tuberculosis* strains.

#### 3.4.2 Molecular Docking against *M. tuberculosis* Homologous Targets

As indicated by the predicted binding affinity values obtained for *T. cruzi*, ligand **1g** showed favorable *in silico* recognition profiles for both cruzain and CYP51. Therefore, for the docking experiments with *M. tuberculosis* , receptors with high sequence and structural homology were selected: a C1 family cysteine protease (homologous to cruzain) and cytochrome P450 14α-sterol demethylase (CYP51). The corresponding accession numbers are provided in the methodology. Structural alignment yielded RMSD values of 1.0 and 1.1 Å, respectively, confirming a high degree of similarity between the *T. cruzi* and *M. tuberculosis* proteins (Figure 4). This similarity may partially rationalize the observed phenotypic activity trends for ligand **1g**. The predicted binding affinities and interaction profiles for each receptor are detailed below.

**Figure 4:**
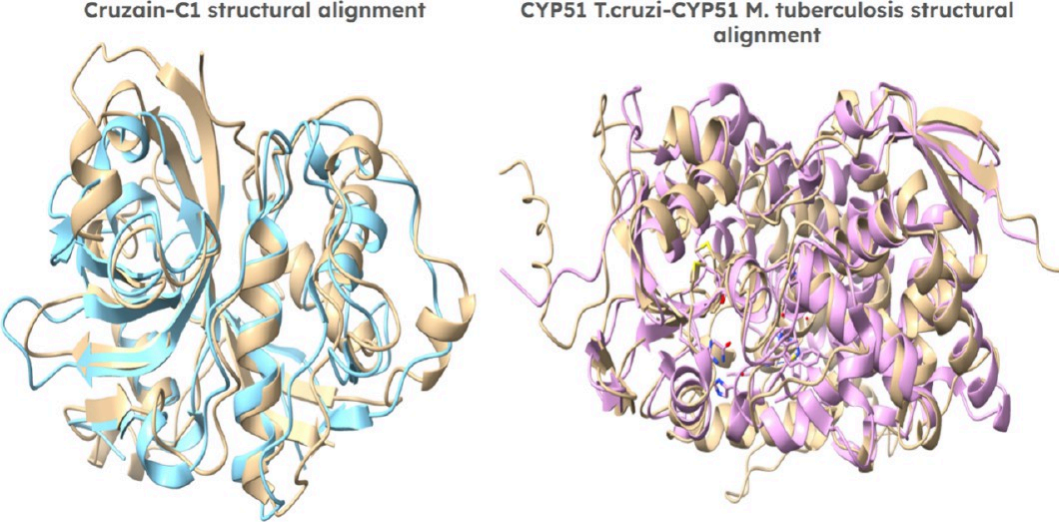
Structural alignment of *T. cruzi* and *M. tuberculosis* receptors. Left: overlay of cruzain with the homologous C1 family cysteine protease, showing an RMSD of 1.0 Å. Right: overlay of CYP51 from *T. cruzi* and *M. tuberculosis* , with an RMSD of 1.1 Å. The high structural similarity supports the selection of these proteins as docking targets and may explain the strong *in vitro* activity observed for ligand **1g**.

#### 3.4.3 C1 Family Cysteine Protease from *M. tuberculosis*

The **1h**–C1 protease complex (Figure 5) exhibited a predicted binding affinity of −7.11 kcal/mol, consistent with moderate affinity values typically reported for cysteine protease inhibitors. Contact analysis revealed a predominance of hydrophobic interactions, with carbon–carbon (CC, apolar) contacts representing 52.6%, followed by carbon–nitrogen (CN, 21.4%) and carbon–oxygen (CO, 15.9%) contacts. Other contributions were minor, and no oxygen–oxygen interactions were detected (OO ≈ 0%), underscoring the absence of an extensive hydrogen-bonding network.

**Figure 5:**
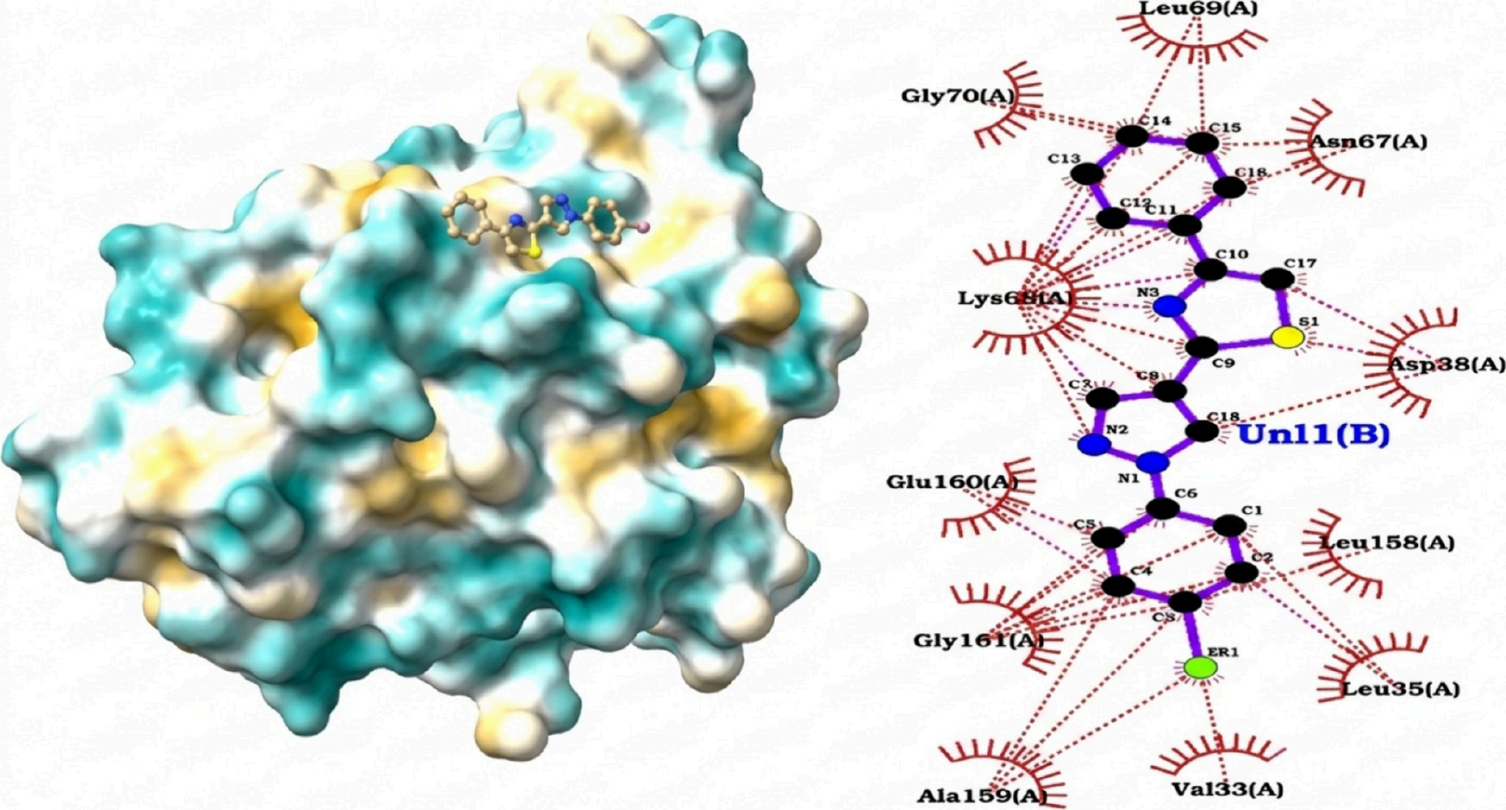
Predicted binding mode of ligand **1h** with the *M. tuberculosis* C1 family cysteine protease. Left: surface representation of the protease showing ligand accommodation in the binding cleft. Right: 2D interaction diagram highlighting dominant hydrophobic contacts (red arcs), a hydrogen bond and cation−π interaction with Lys68, and additional apolar contributions from Leu35, Asp67, and Glu158, supporting a binding mode primarily driven by hydrophobic interactions with limited polar stabilization.

At the residue level, apolar contacts were identified with Leu35 (3.44 Å) and Lys68 (3.97 Å), where the methylene chain of Lys68 contributed to the hydrophobic environment. A hydrogen bond was detected between the protonated amino group of Lys68 (N3^+^ donor) and an aromatic atom of the ligand (distance = 3.48 Å; angle = 124.6°), though its geometry suggests only modest energetic relevance. Additionally, a cation−π interaction between Lys68 and an aromatic ring of the ligand (3.68 Å; offset ≈ 1.24 Å) contributed to complex stabilization.

Overall, this binding mode-dominated by hydrophobic contacts with a single polar contribution-resembles the profiles described by Petushkova et al.,^68^ supporting the notion that substrate specificity in cysteine proteases is primarily dictated by the structural features of the binding cleft (pocket size, electrostatics, and shape). In comparison with cruzain and CYP51, the **1h** ligand appears less oriented toward the catalytic dyad and more dependent on apolar packing, which likely explains its lower binding affinity.

#### 3.4.4 Cytochrome P450 14α-Sterol Demethylase (CYP51)

The **1g**–CYP51 complex in *M. tuberculosis* (Figure 6) exhibited a predicted binding free energy of −8.34 kcal/mol, corresponding to predicted submicromolar affinity and consistent with the known propensity of this P450 enzyme to recognize lipophilic ligands. The contact profile was dominated by hydrophobic interactions (CC = 48.2%), with additional contributions from CN (25.2%) and CO (15.7%) contacts, while purely polar interactions (NO, NN) were rare (<3%).

**Figure 6:**
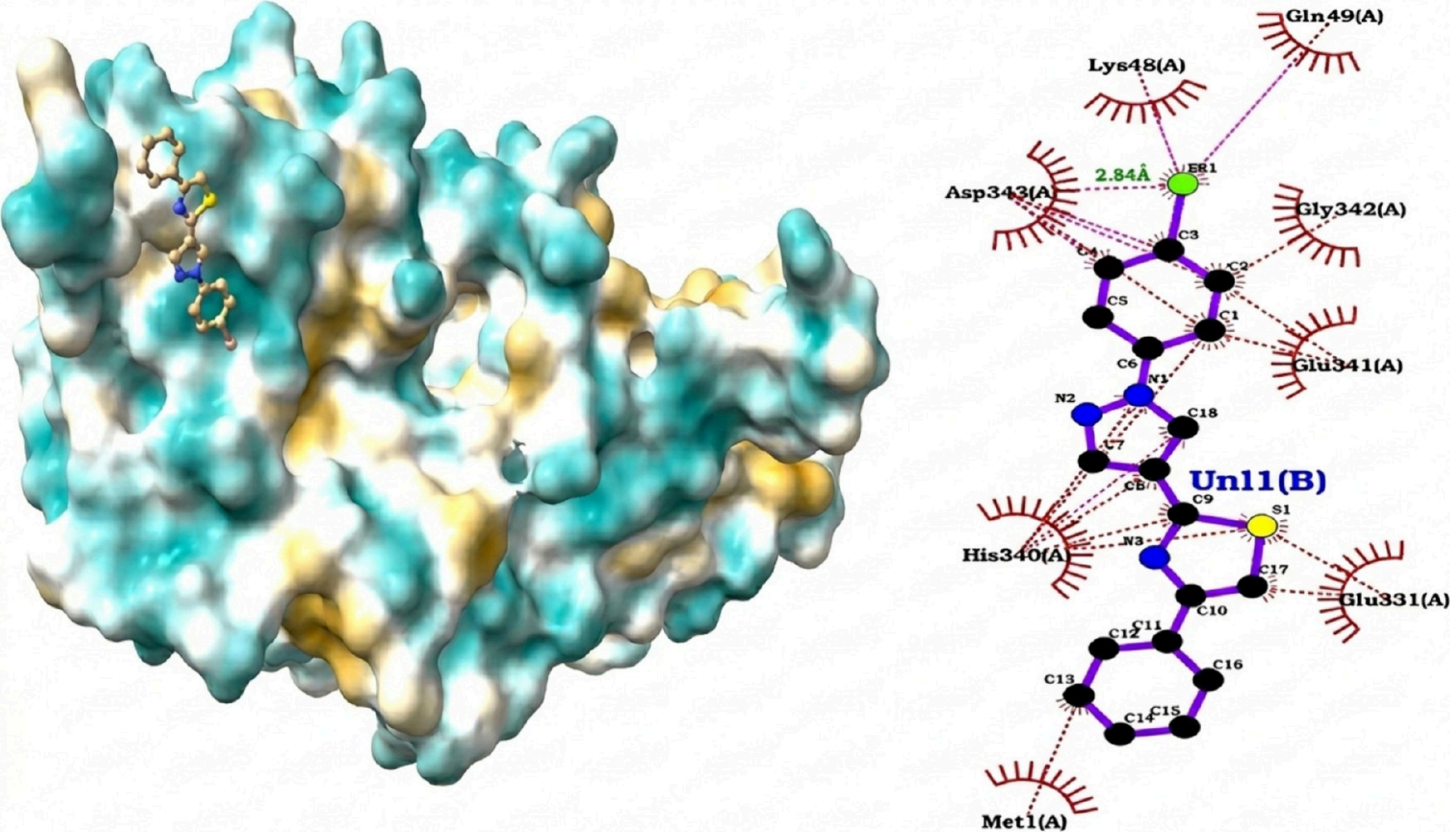
Predicted binding mode of ligand **1g** with *M. tuberculosis* CYP51. Left: surface representation of CYP51 showing the ligand accommodated in the hydrophobic channel. Right: 2D interaction diagram highlighting dominant apolar contacts (red arcs), apolar contact with Asp343, and two π–cation interactions with His340 that compensate for the absence of hydrogen bonds, consistent with a stabilization mechanism driven by hydrophobic and aromatic interactions.

At the residue level, the ligand engaged in apolar contacts with Asp343 and established two key π–cation interactions with His340 (distances 4.29 and 4.37 Å; offset ≈ 1.85–2.00 Å), in which the protonated imidazole ring stabilized the aromatic moiety of the ligand. These aromatic interactions compensate for the absence of an extensive hydrogen-bonding network, indicating that ligand stabilization occurs primarily through hydrophobic packing and π–cation contacts within the heme access channel.

This observation aligns with previous findings by Shi et al.,^66^ who demonstrated, through molecular dynamics simulations, that the CYP51 access channel is largely stabilized by hydrophobic residues such as F58, Y64, Y118, L121, Y132, L376, S378, S506, S507, and M508. Collectively, these results suggest that CYP51 recognition of **1g** relies on a predominantly apolar pocket reinforced by π–cation interactions, with minimal contribution from directed polar contacts. Nevertheless, given the limited validation of CYP51 as a therapeutic target in *M. tuberculosis* , these results should be interpreted strictly as structural and exploratory insights, rather than evidence of a druggable mechanism.

Across the series, biological activity is strongly influenced by both the nature and position of halogen substituents on the aryl–pyrazole moiety, with distinct and nonoverlapping trends observed for *T. cruzi* and *M. tuberculosis* . Fluorinated derivatives, particularly the *meta*-fluoro analogue **1g**, displayed the most consistent activity against *T. cruzi*, and this behavior correlates with a combination of moderate lipophilicity (cLogP ≈ 3.2–3.5), controlled polarity (tPSA ≈ 59 Å^2^), and comparatively higher predicted solubility within the series. In contrast, within the antimycobacterial data set, measurable activity was restricted to the *para*-brominated derivative **1h**, indicating that alternative substitution patterns and physicochemical profiles are required to achieve activity in this model. Importantly, no direct correlation between activity and any single physicochemical descriptor was observed across both pathogens. Dichlorinated derivatives **1b**, **1c**, **1d**, and **1k** showed limited or inconsistent activity in both assays, consistent with deviation from the narrow physicochemical window associated with measurable activity in this series. Overall, the data indicate that activity within this scaffold is governed by a pathogen-dependent interplay between halogen identity, substitution pattern, and global physicochemical properties, rather than by the presence of a single substituent or a uniform trend across biological models.

## 4 Conclusions

In this work, 11 new pyrazole-thiazole hybrids **1**(**a–k**) were synthesized in 27–91% yields. This study delineates pyrazole–thiazole hybrids as a chemically accessible scaffold displaying differentiated phenotypic activity against *T. cruzi* and **M. tuberculosis*,* with bioactivity strongly modulated by halogen substitution patterns. Subtle structural variations, particularly fluorine and bromine incorporation, were shown to influence activity profiles across the two pathogens, highlighting the sensitivity of this chemotype to minor chemical modifications. While the observed MIC values remain modest when compared with standard reference drugs, the combined biological evaluation and computational analyses provide coherent structure–activity trends. In particular, hydrophobic and aromatic interactions emerge as dominant contributors to scaffold performance, with molecular docking supporting the use of CYP51 as a structural reference model to rationalize these interactions, rather than as evidence of target engagement. Overall, these findings establish a structure–activity framework that may inform future optimization efforts within this chemical class. Further chemical refinement and expanded biological characterization will be required to assess whether pyrazole–thiazole hybrids can be advanced toward improved antimycobacterial or antiparasitic profiles.

## Supplementary Material



## Notes

(Figures S1–S11) FT-IR spectra of compounds **1**(**a**–**k**), (Figures S12–S33) NMR spectra of compounds **1**(**a**–**k**), and (Figures S34–S44) HRMS spectra of compounds **1**(**a**–**k**) (PDF)
