# Supplementary material for: Pyrazole-Thiazole Hybrids: Synthesis and Biological Evaluation against Trypanosoma cruzi and Mycobacterium tuberculosis
Source: ACS Omega. 2026 Apr 1;11(14):21890–902. doi: 10.1021/acsomega.5c12469 (PMC13084514; doi:10.1021/acsomega.5c12469)

Pyrazole-thiazole hybrids: Synthesis and biological evaluation against *Trypanosoma cruzi* and *Mycobacterium tuberculosis*

Cynthia Nathália Pereira[1], Lorraine Martins Rocha Orlando[2], Edinaldo Castro de Oliveira[2], Mirian Claudia de Souza Pereira[2], Christian S. Canales Carnero[3], Oswaldo Julio Ramirez Delgado[4], Cesar Augusto Roque-Borda[4,5*], Fernando Rogério Pavan[4], Maurício Silva dos Santos[1,*]

[1] Laboratório de Síntese de Sistemas Heterocíclicos (LaSSH), Institute of Physics and Chemistry, Federal University of Itajubá, 1303 BPS Avenue, Pinheirinho, Itajubá-MG, 37500-903, Brazil

[2] Laboratório de Ultraestrutura Celular, Instituto Oswaldo Cruz, Fiocruz, 4365 Brasil Avenue, Rio de Janeiro-RJ, 21040-900, Brazil

[3] BIOMET Laboratory, National University of Engineering, Rimac, Lima, Peru.

[4] School of Pharmaceutical Sciences, São Paulo State University (UNESP), 14800903, Araraquara, Brazil

[5]Vicerrectorado de Investigación, Universidad Católica de Santa María, Arequipa, 04000, Peru.

**Corresponding author: Tel. (+55) 35 3629-1628; e-mail mauriciosantos@unifei.edu.br and cesar.roque@ucsm.edu.pe*

Supporting Information

List of abbreviations: FT-IR- Fourier transform Infrared spectroscopy

NMR-Nuclear Magnetic Resonance

HRMS- High Resolution mass spectrometry

Figures S1-S11: FT-IR spectrum of compounds **1(a-k)**

Figures S12-S33: NMR of compounds **1(a-k)**

Figures S34-S44: HRMS of compounds **1(a-k)**

Figure S1: FT-IR spectrum of compound **1a**

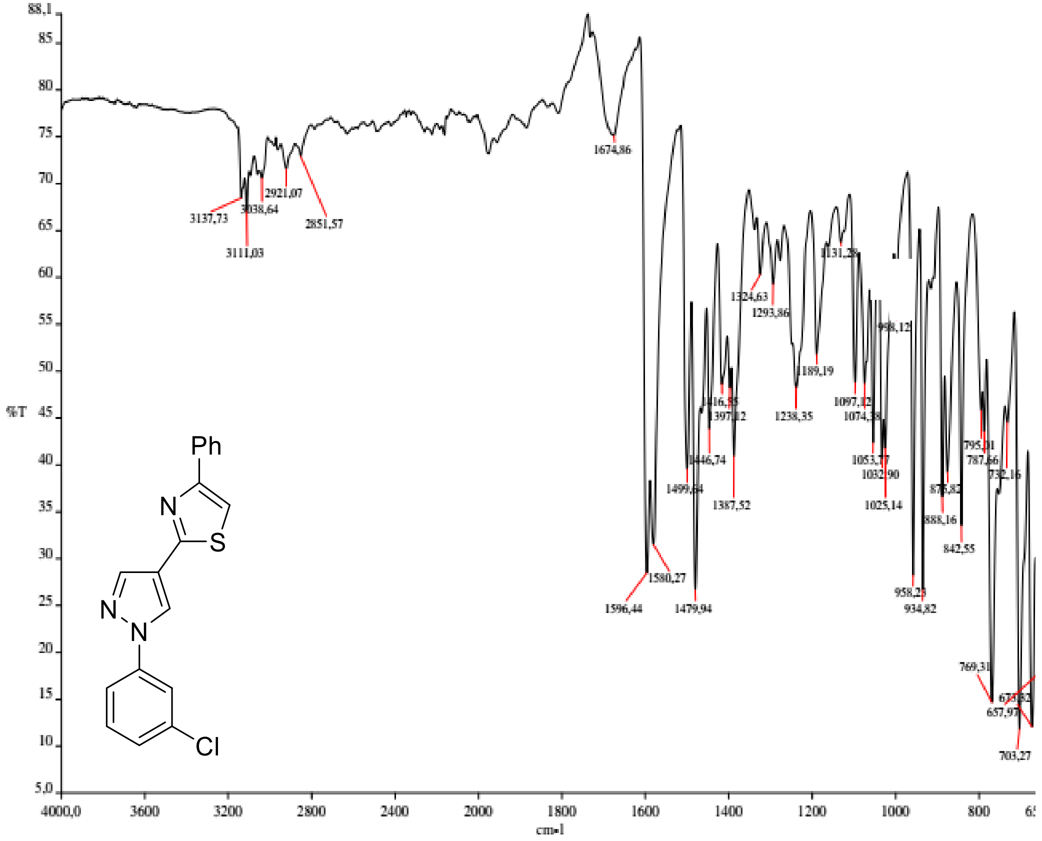

Figure S2: FT-IR spectrum of compound **1b**

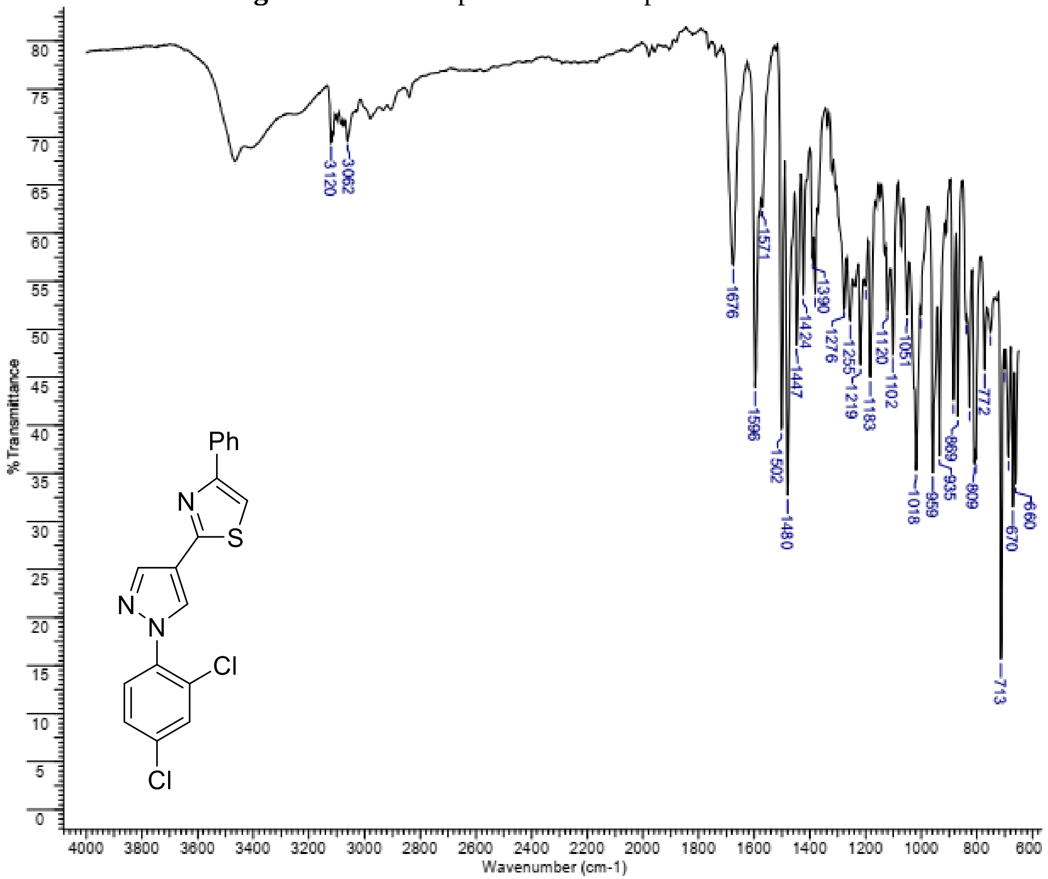

Figure S3: FT-IR spectrum of compound **1c**

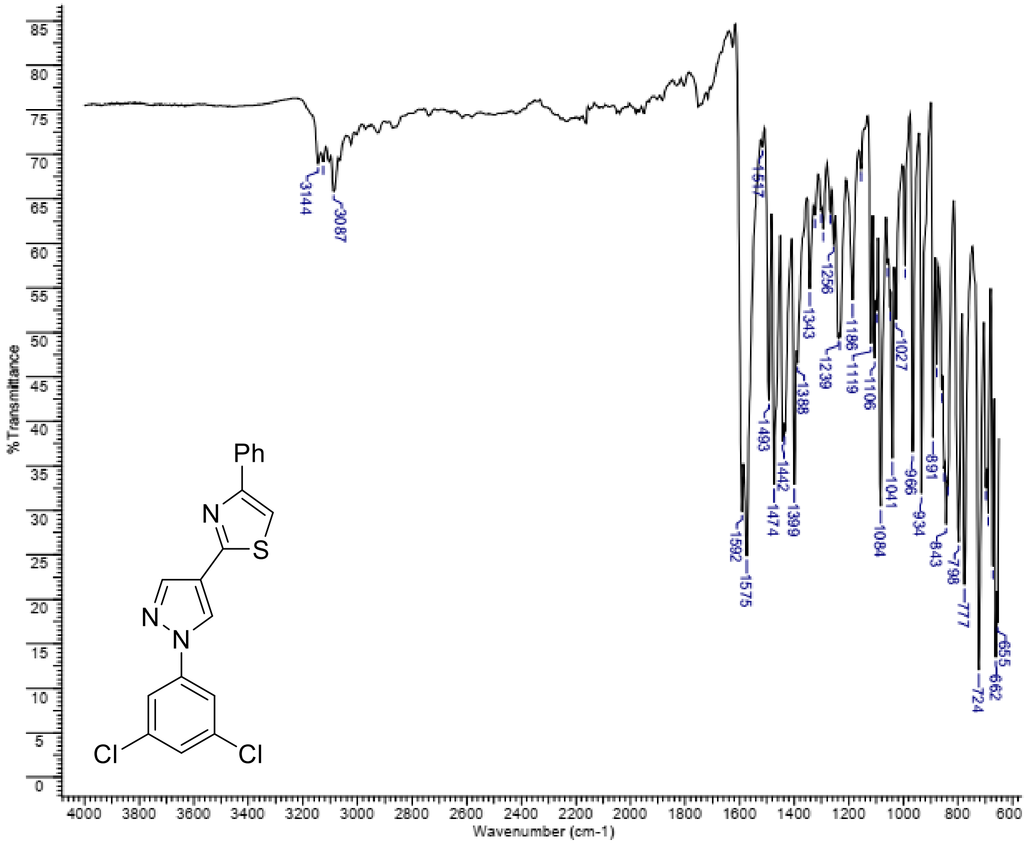

Figure S4: FT-IR spectrum of compound **1d**

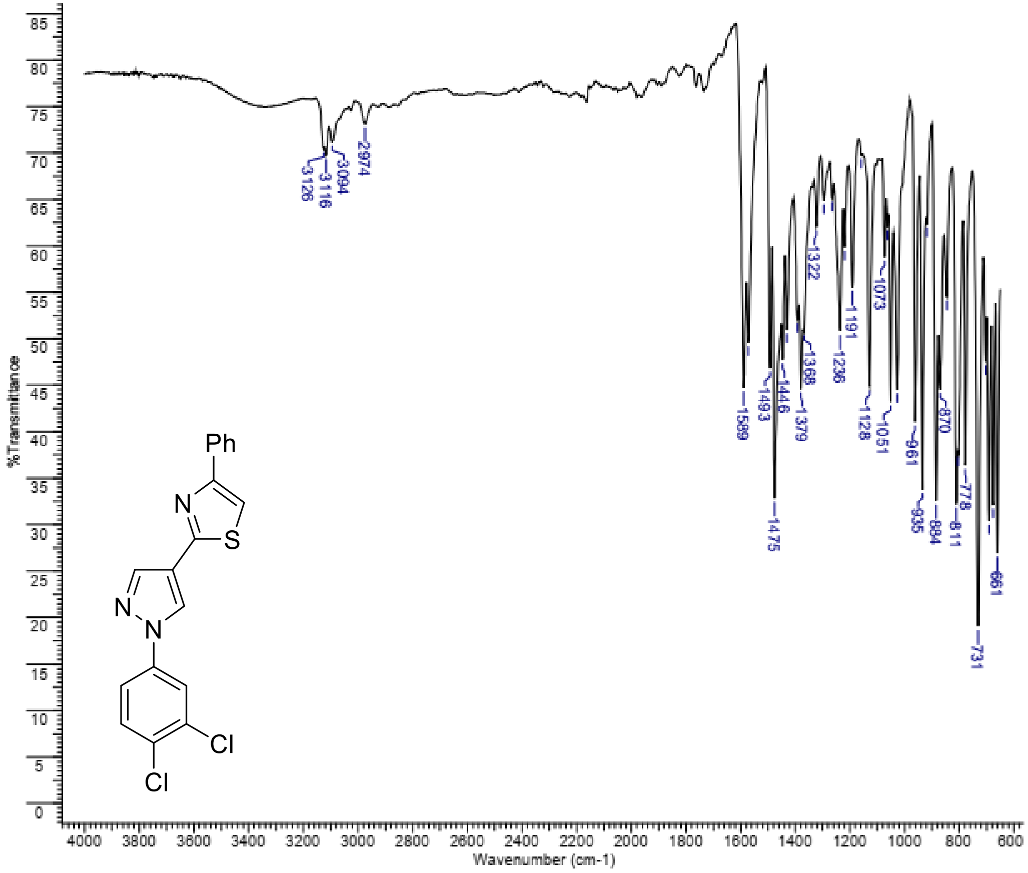

Figure S5: FT-IR spectrum of compound **1e**

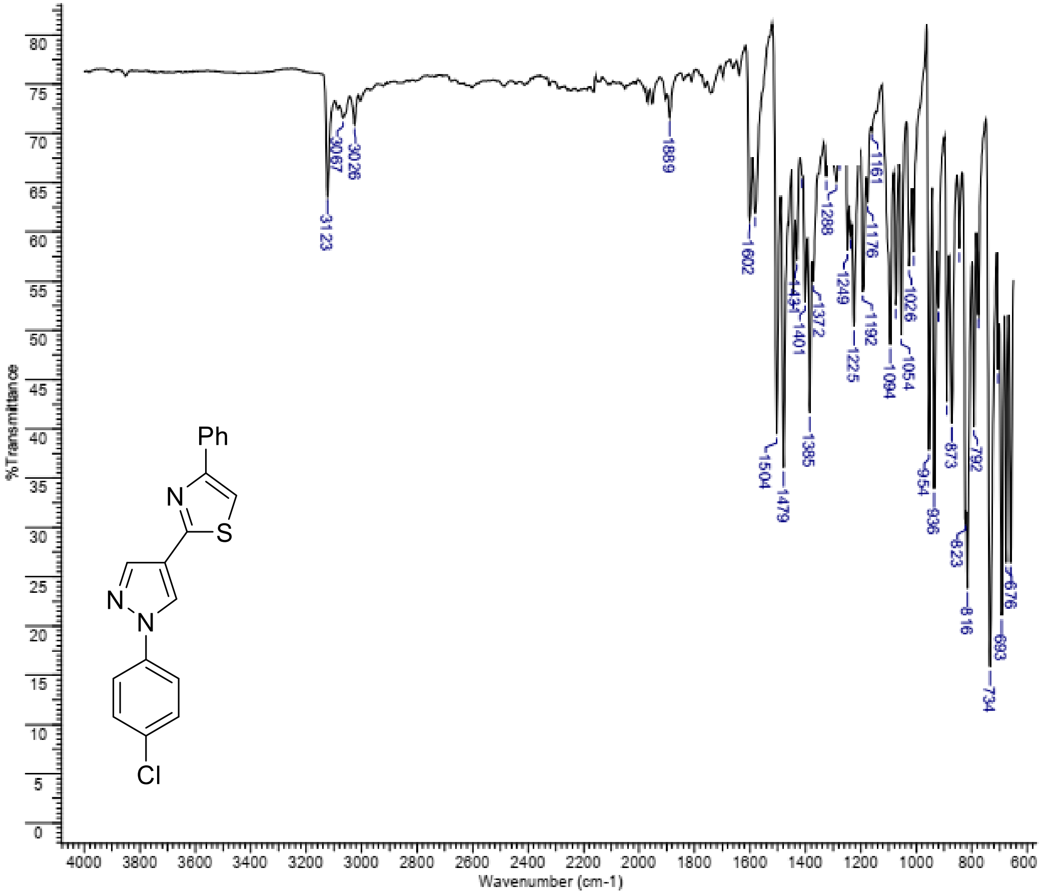

Figure S6: FT-IR spectrum of compound **1f**

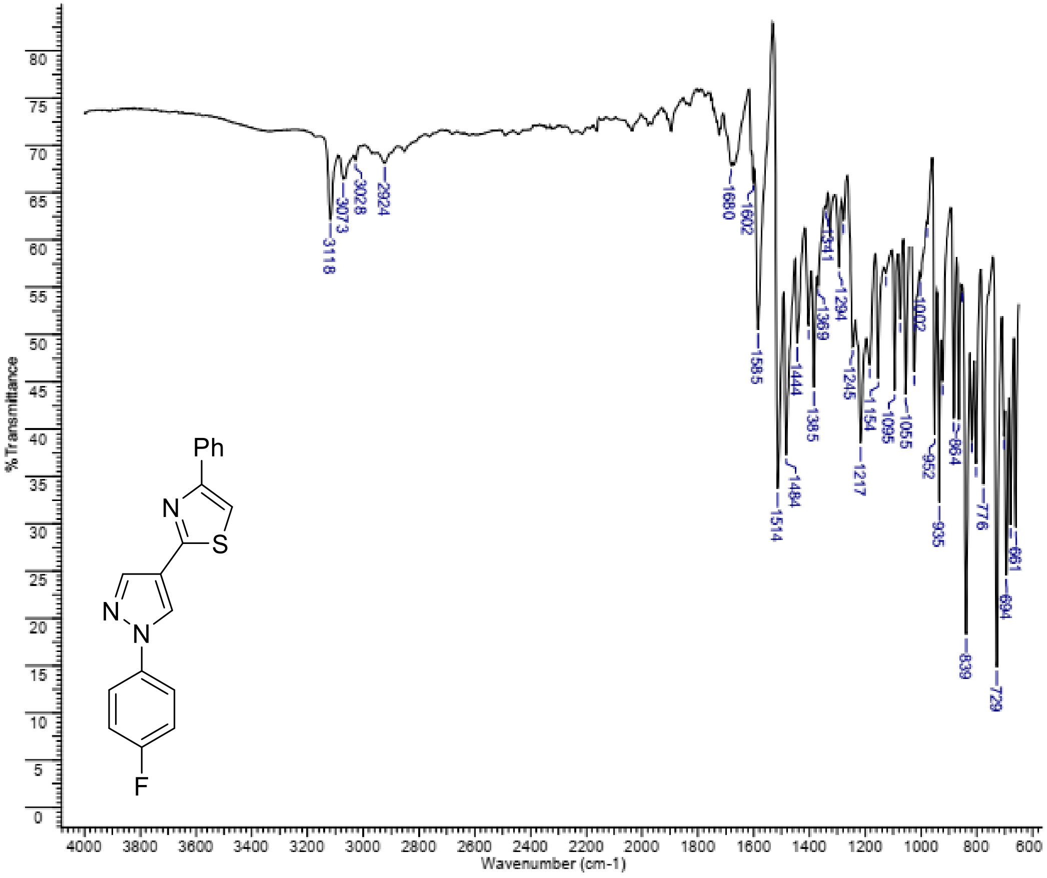

Figure S7: FT-IR spectrum of compound **1g**

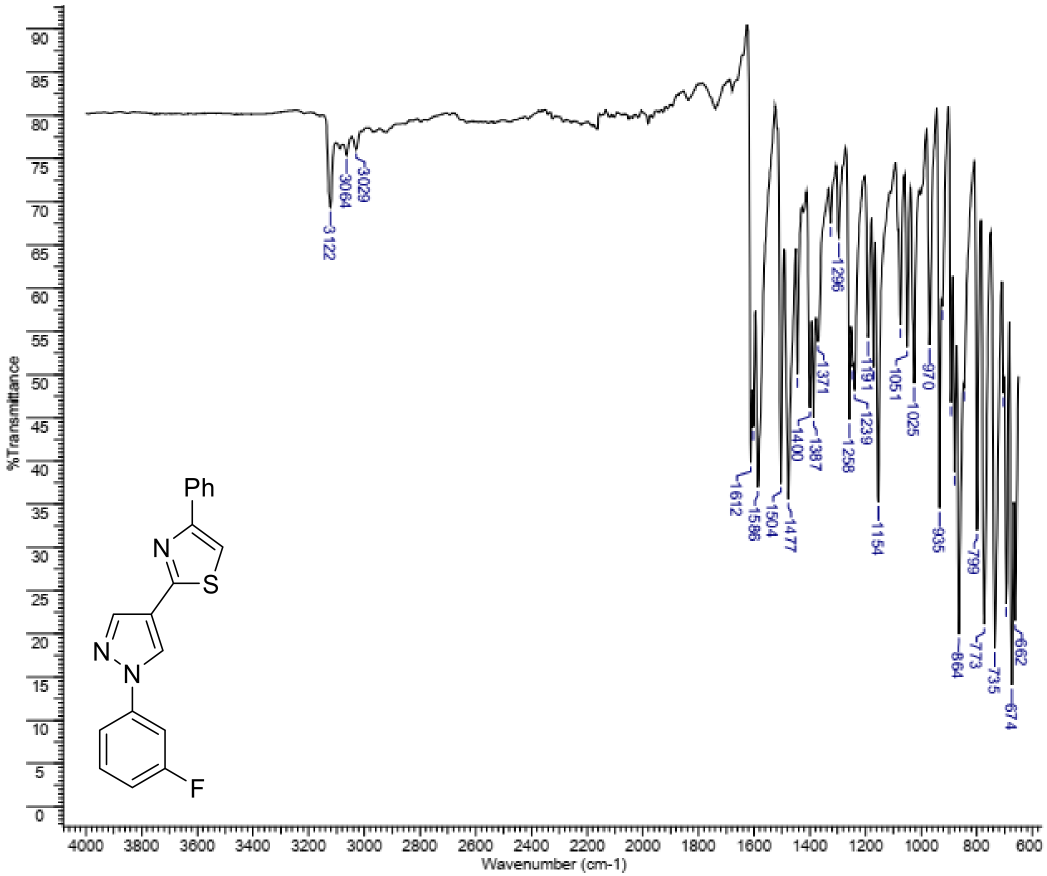

Figure S8: FT-IR spectrum of compound **1h**

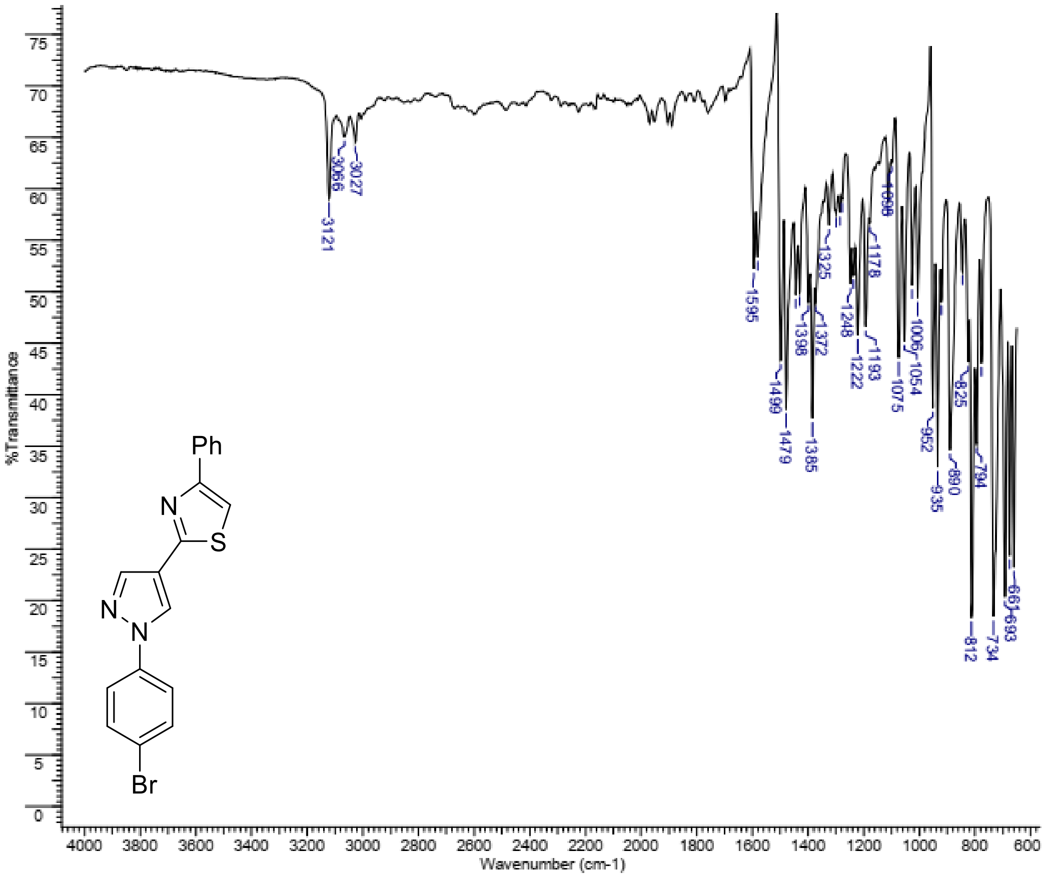

Figure S9: FT-IR spectrum of compound **1i**

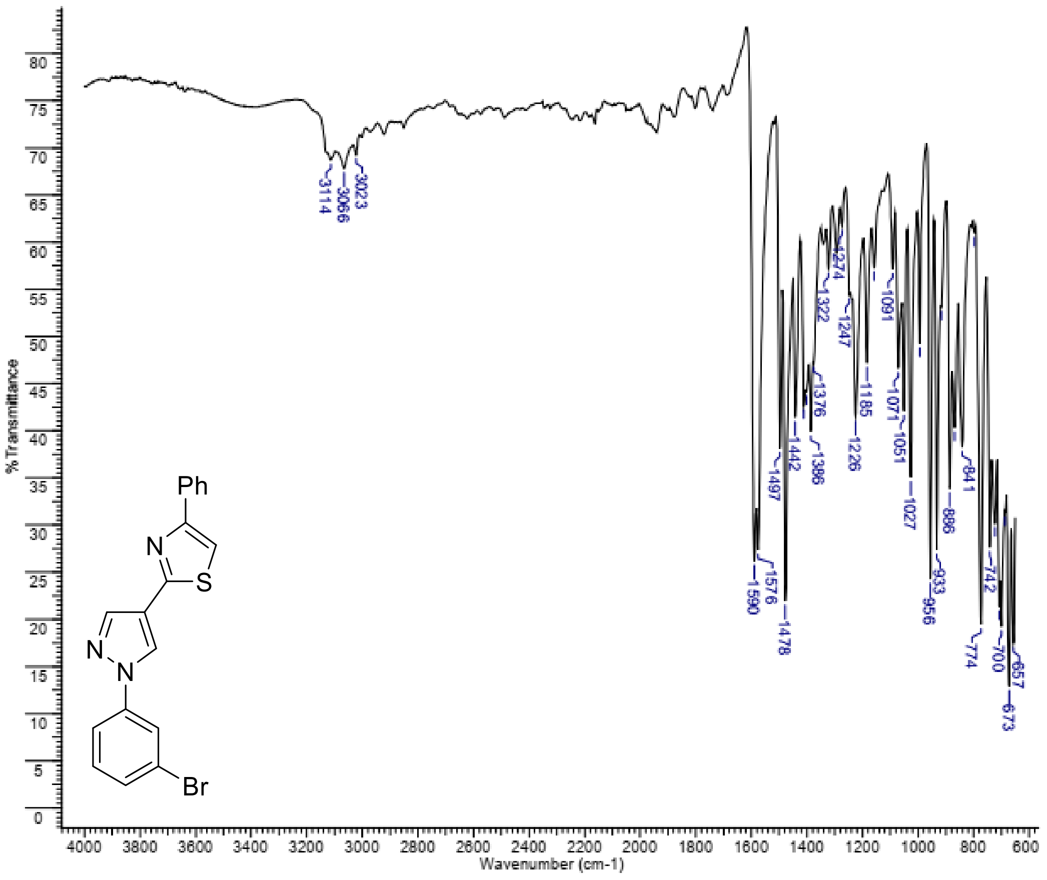

Figure S10: FT-IR spectrum of compound **1j**

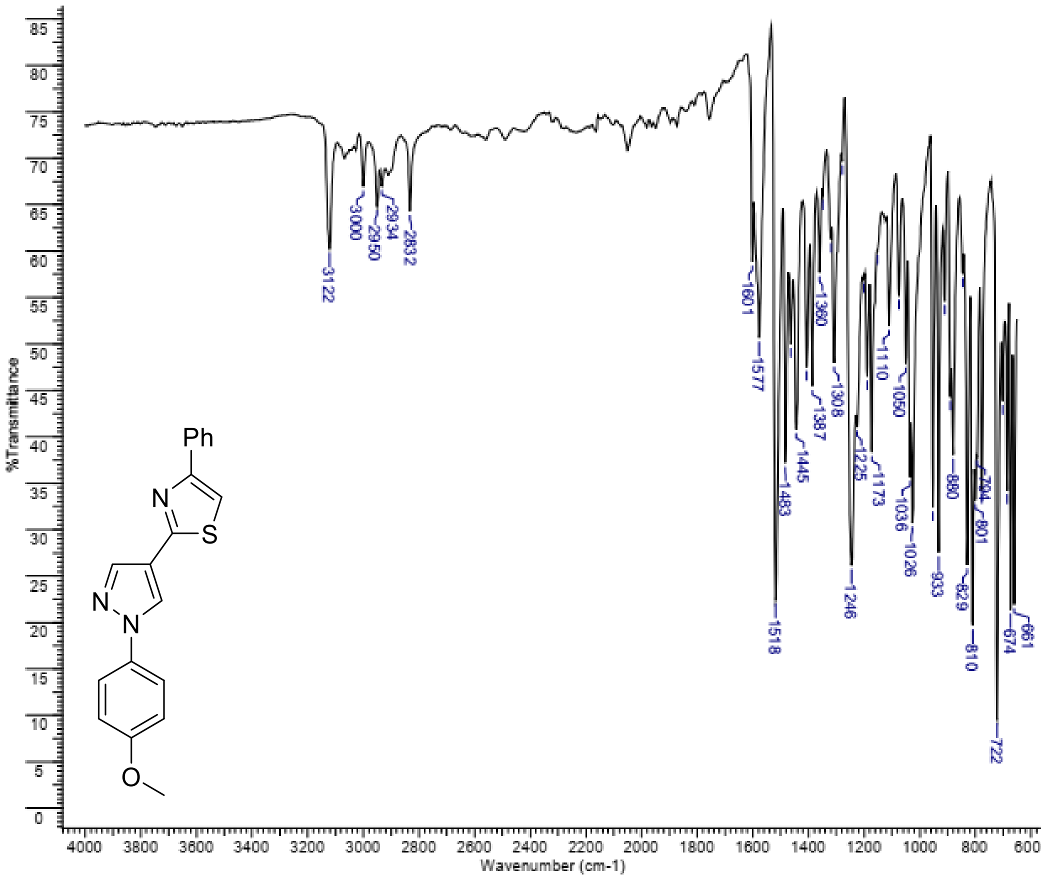

Figure S11: FT-IR spectrum of compound **1k**

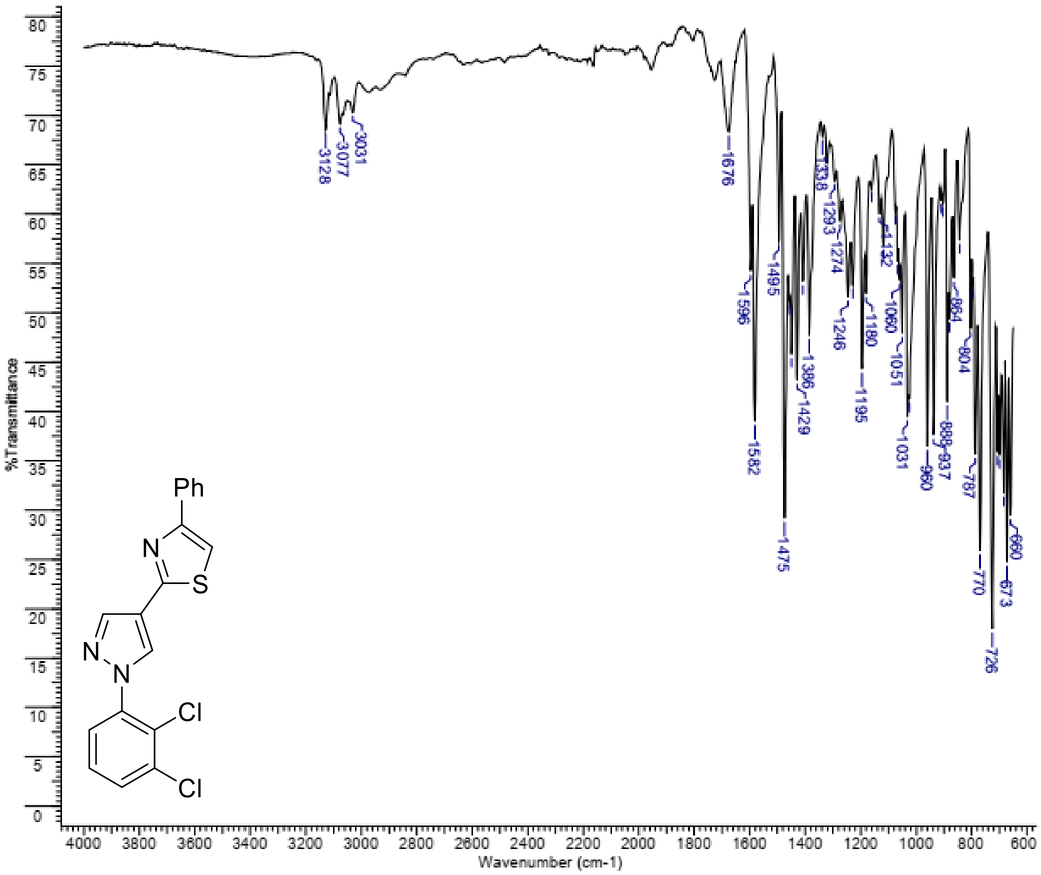

Figure S12: ^{1}H NMR of compound **1a**

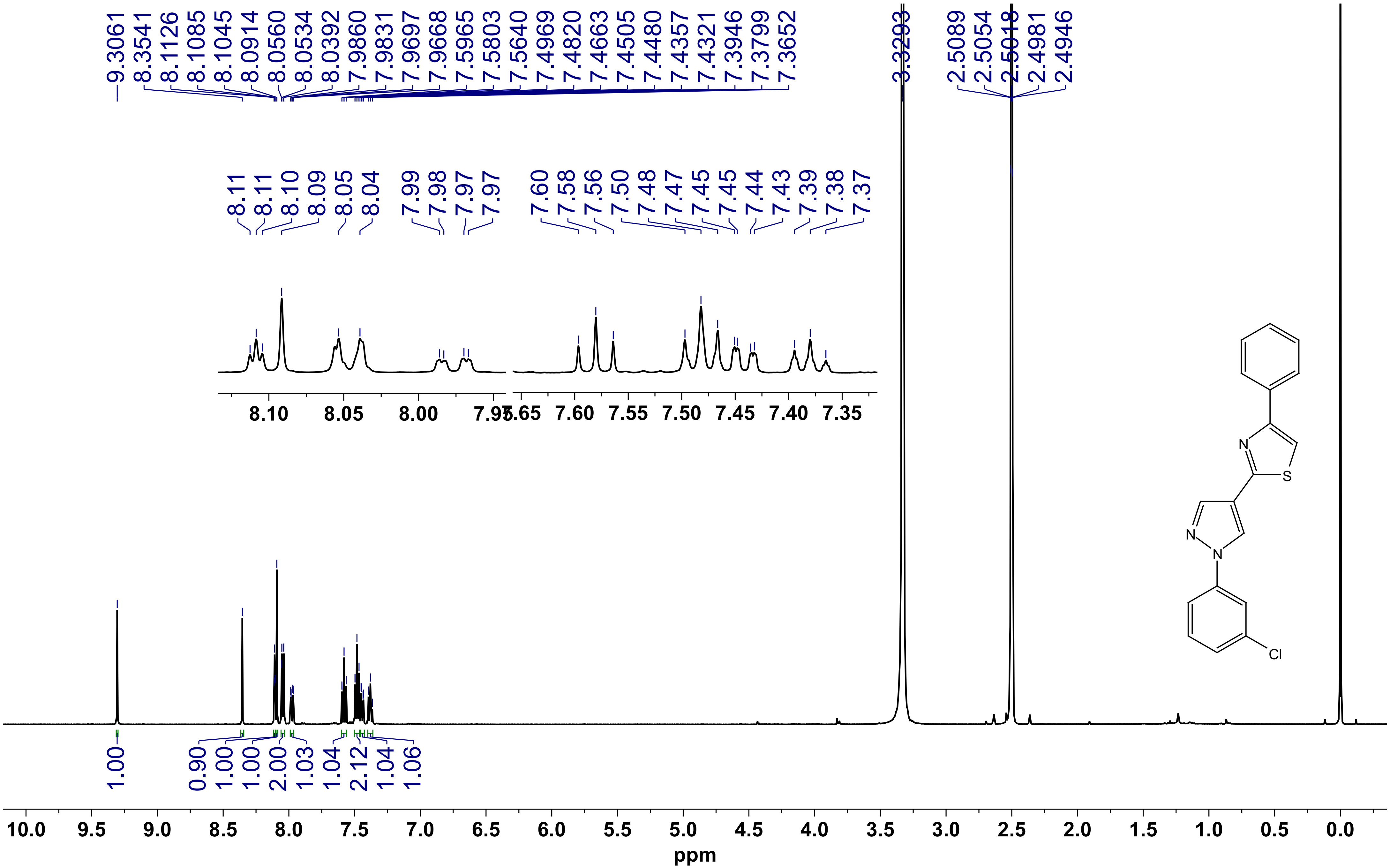

Figure S13: ^{1}H NMR of compound **1b**

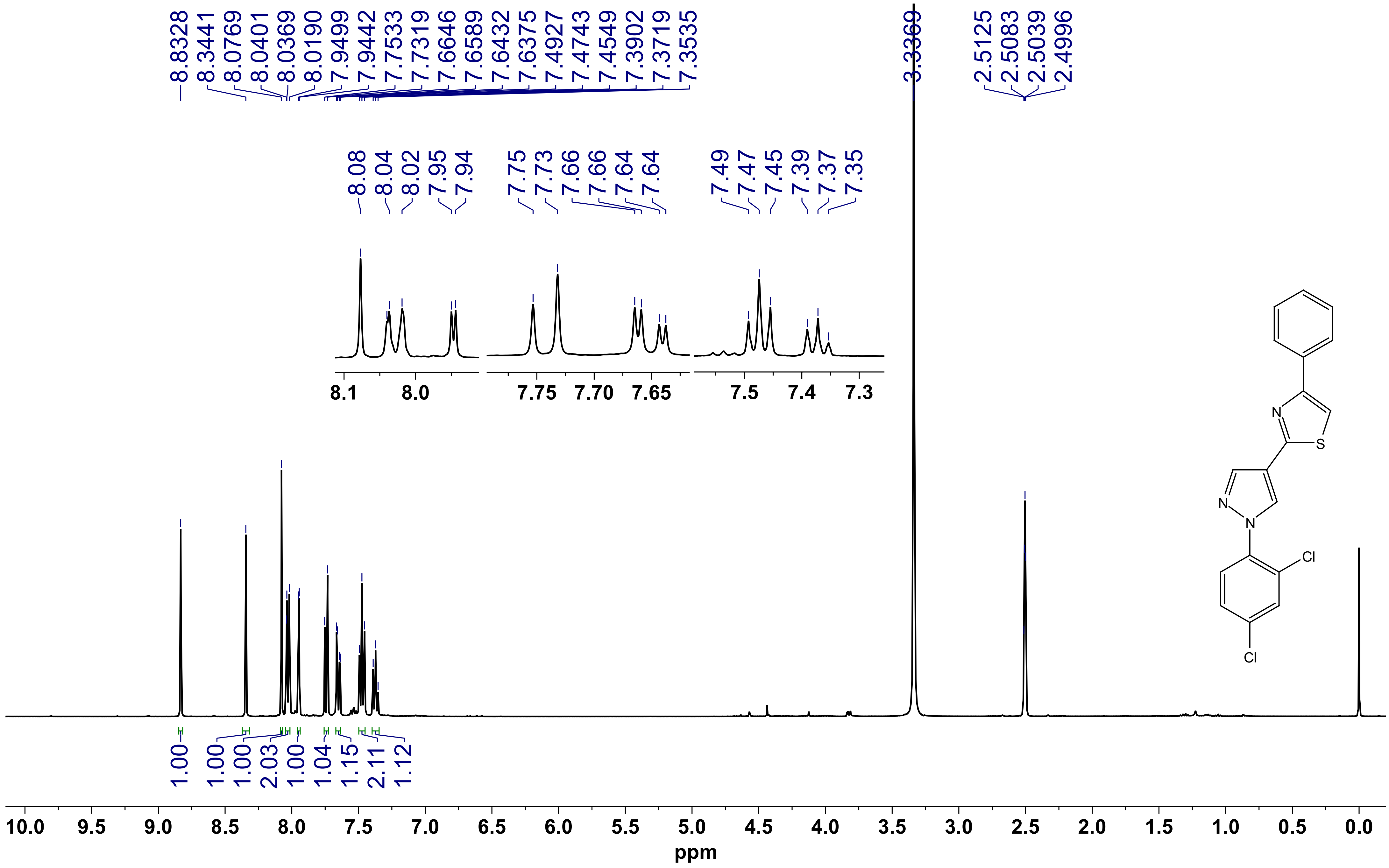

Figure S14: ^{1}H NMR of compound **1c**

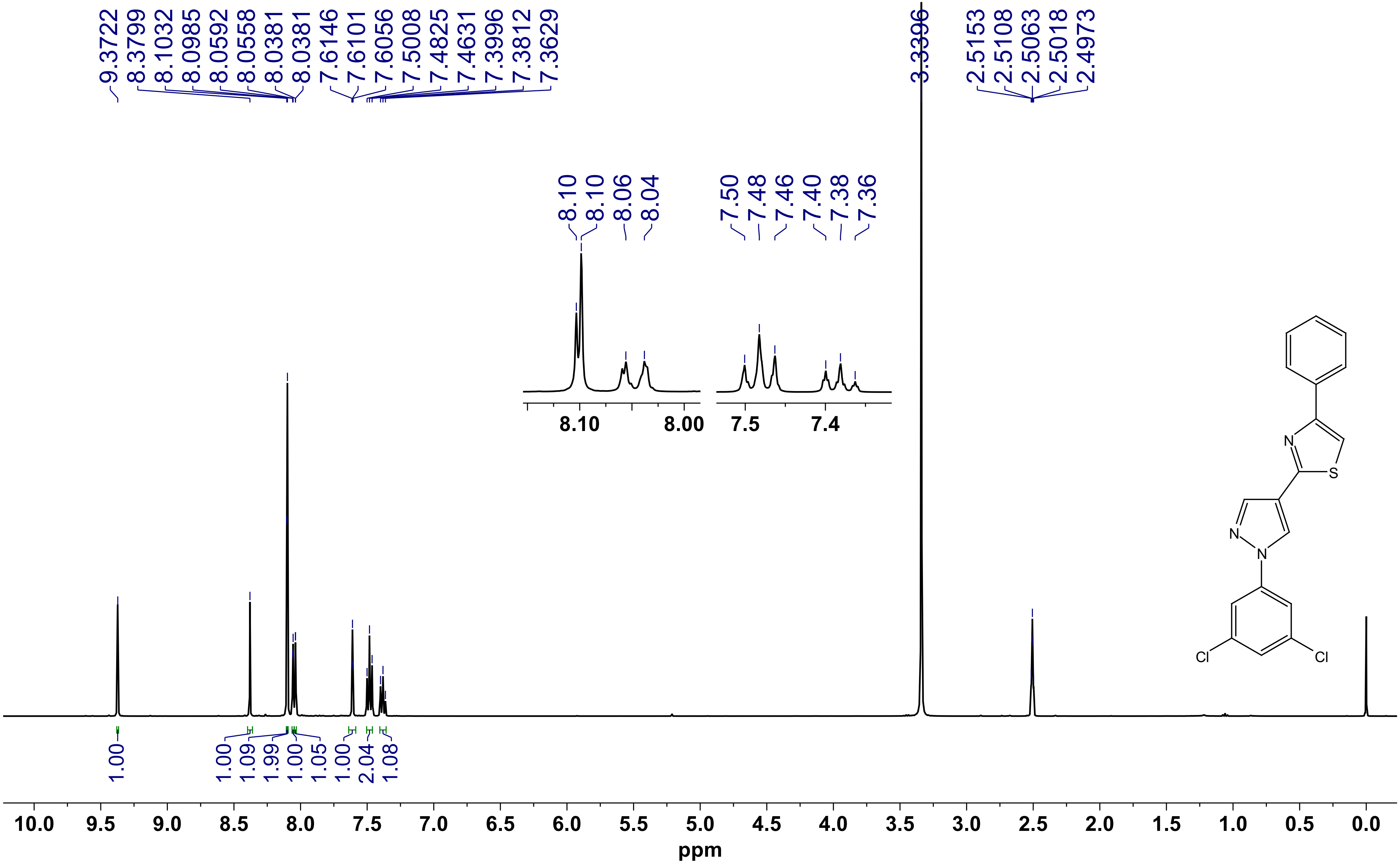

Figure S15: ^{1}H NMR of compound **1d**

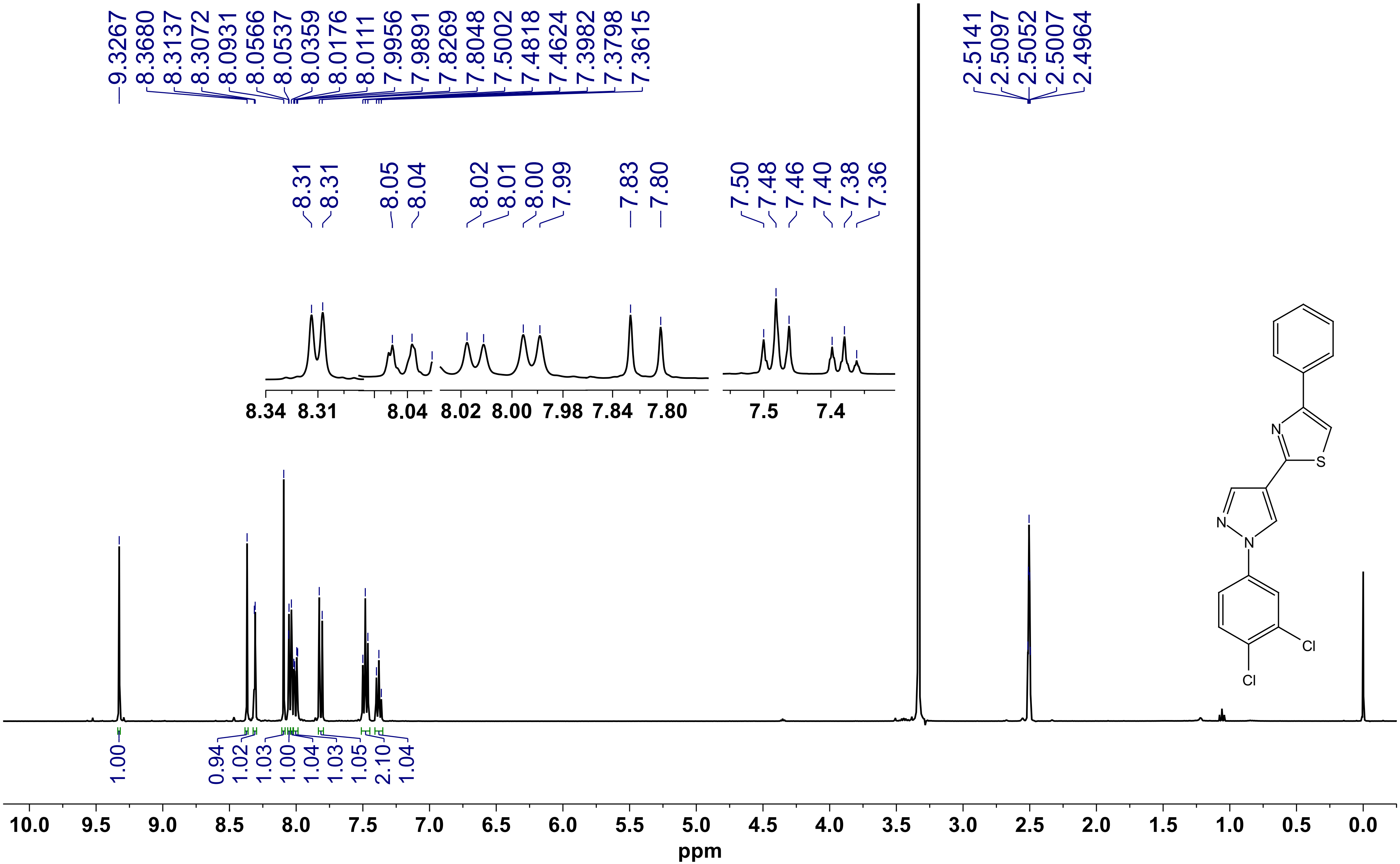

Figure S16: ^{1}H NMR of compound **1e**

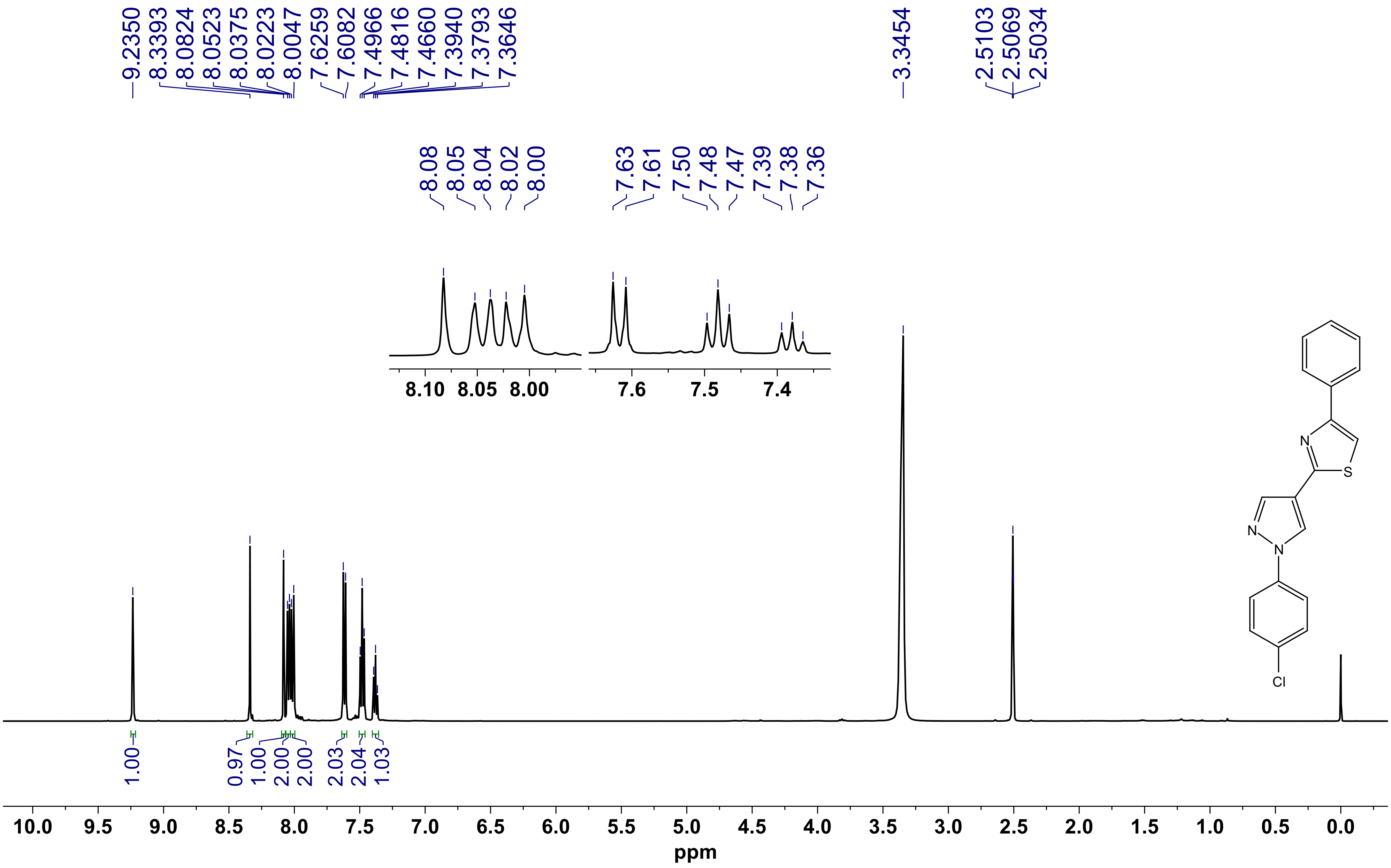

Figure S17: ^{1}H NMR of compound **1f**

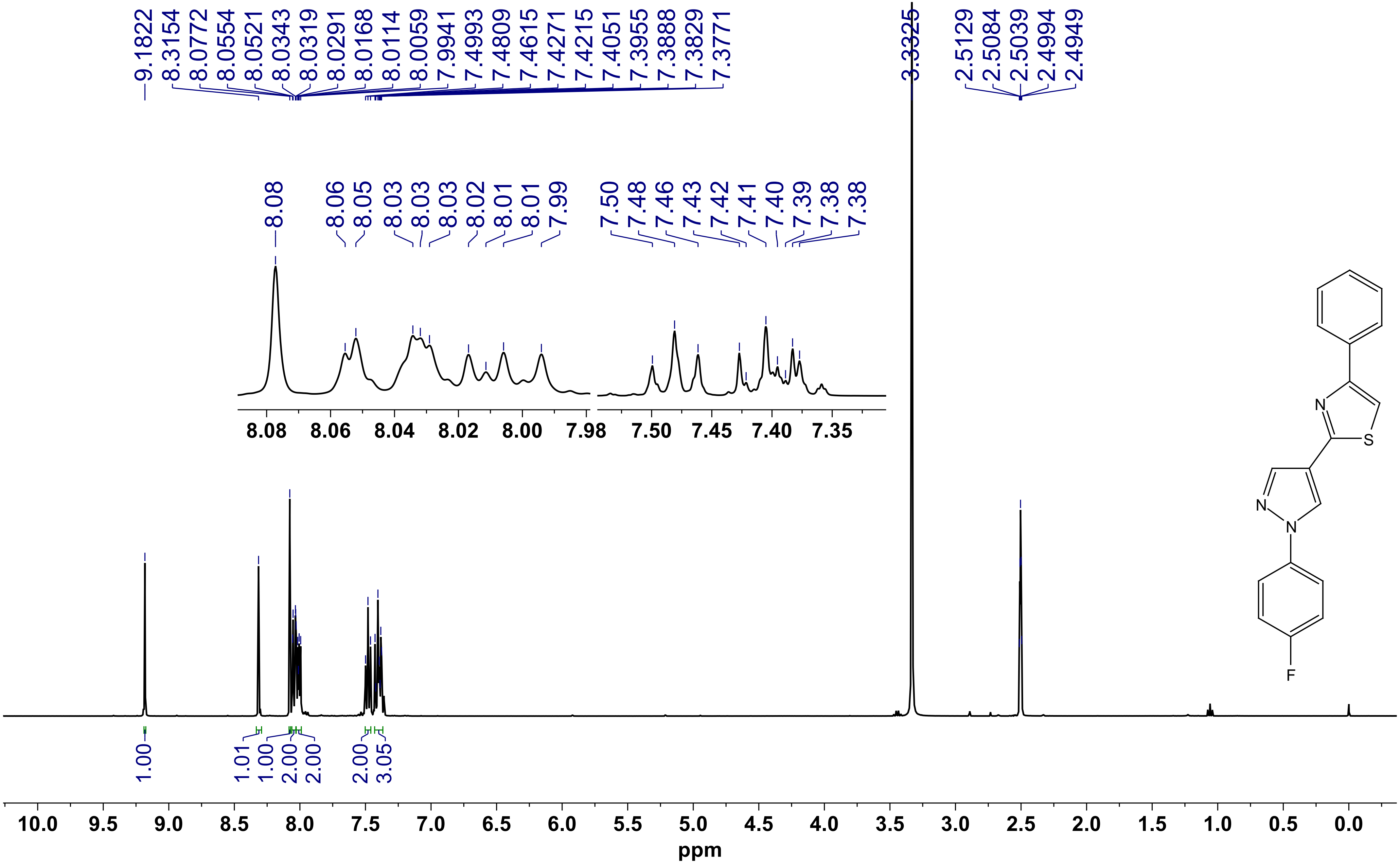

Figure S18: ^{1}H NMR of compound **1g**

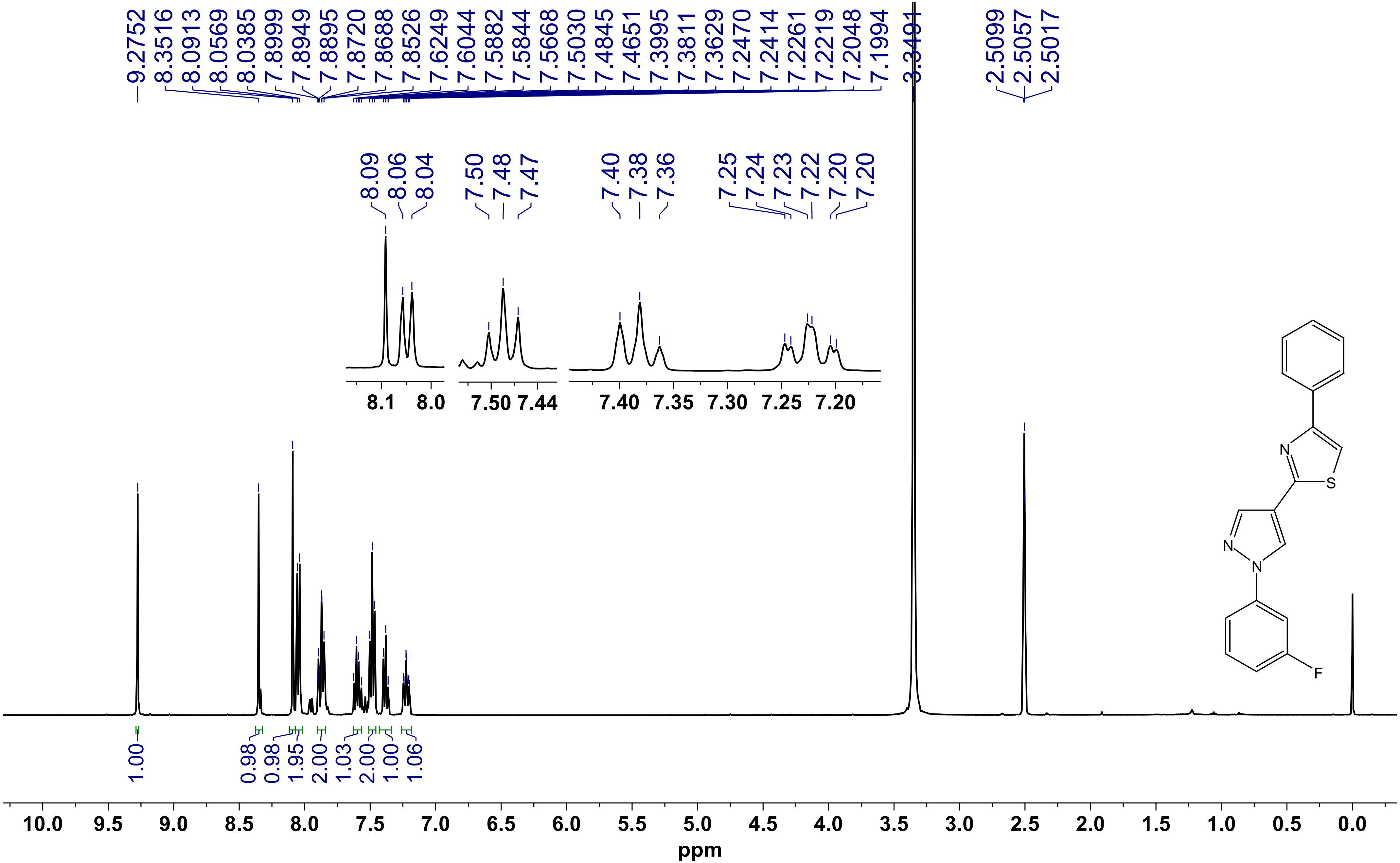

Figure S19: ^{1}H NMR of compound **1h**

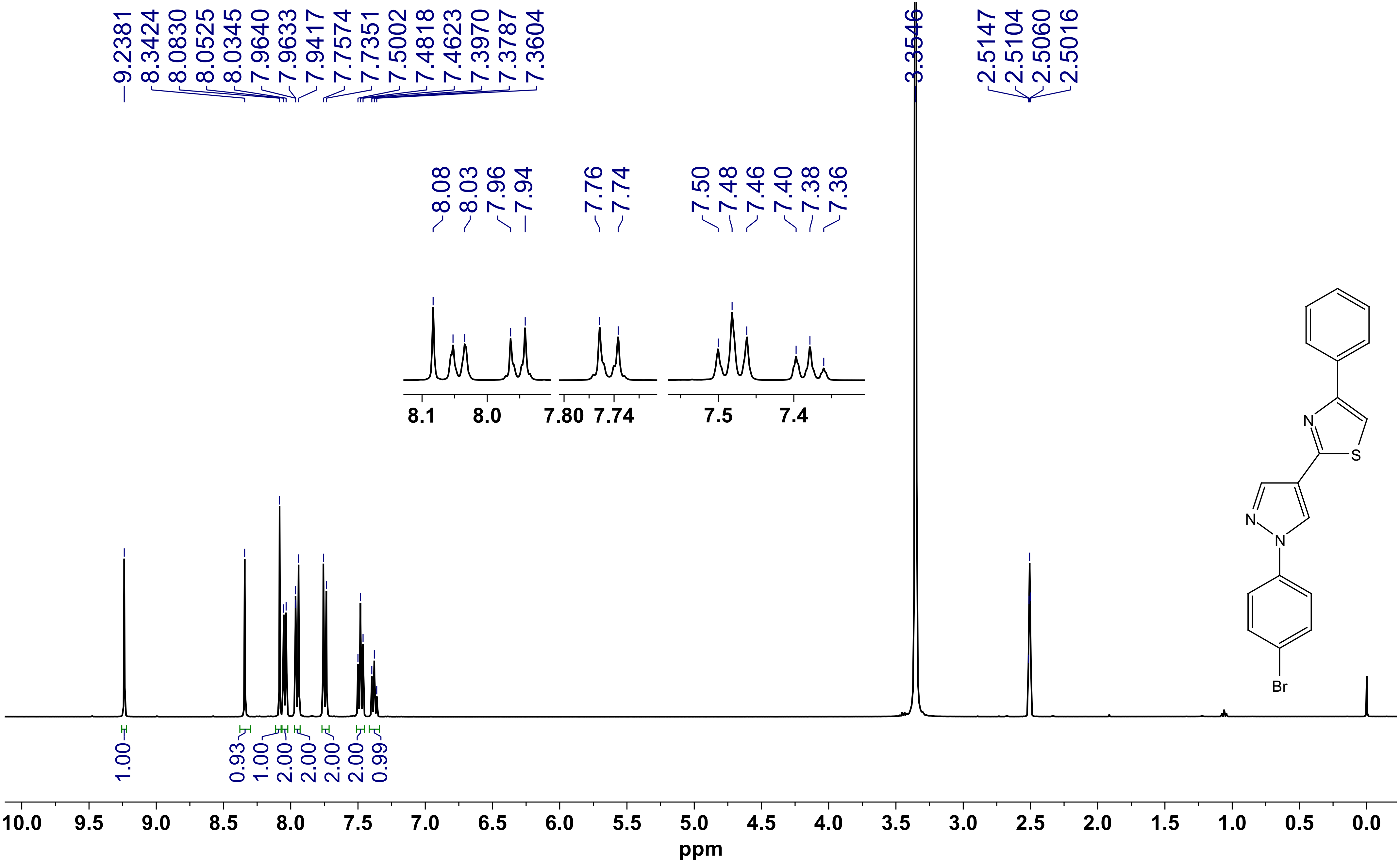

Figure S20: ^{1}H NMR of compound **1i**

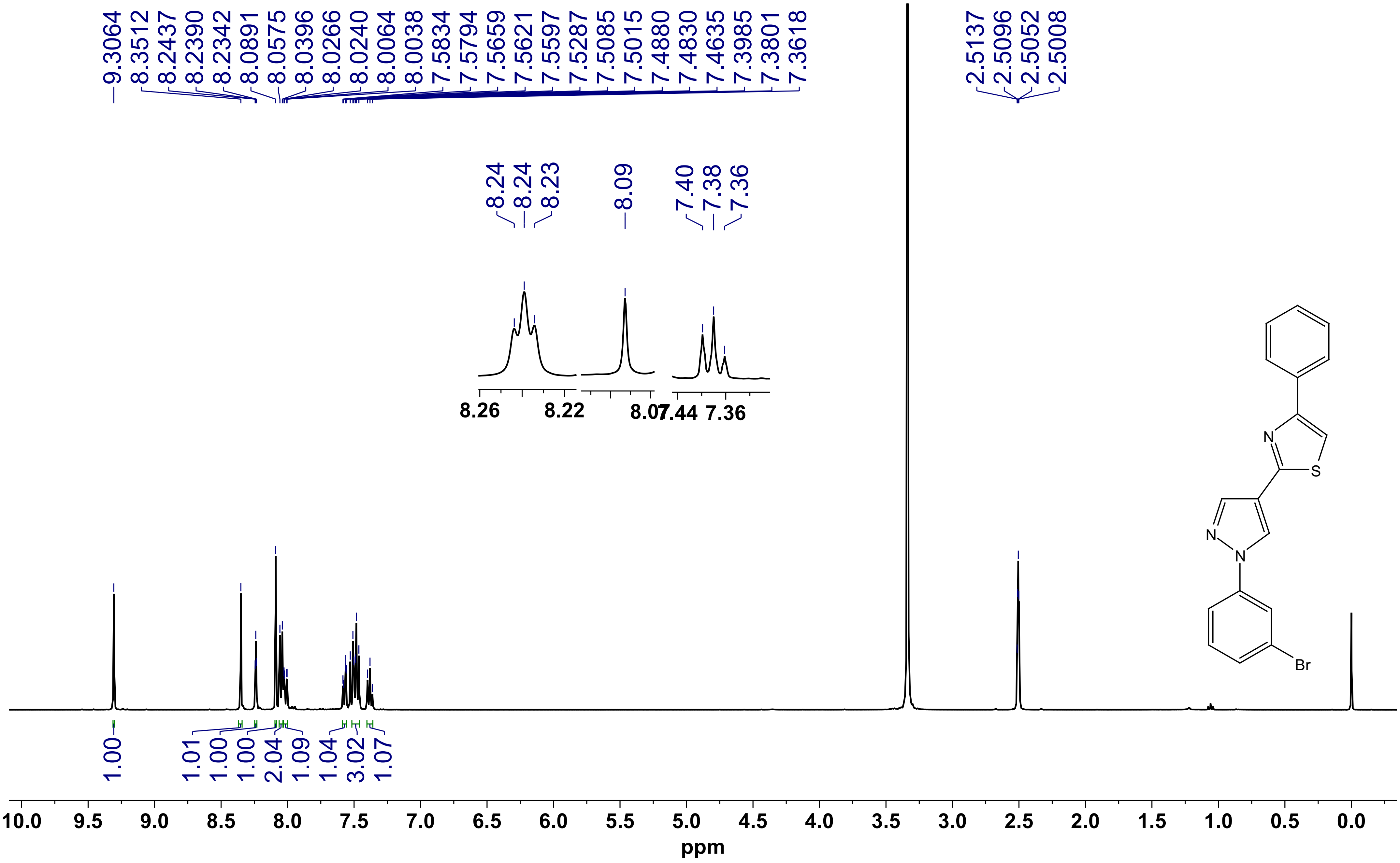

Figure S21: ^{1}H NMR of compound **1j**

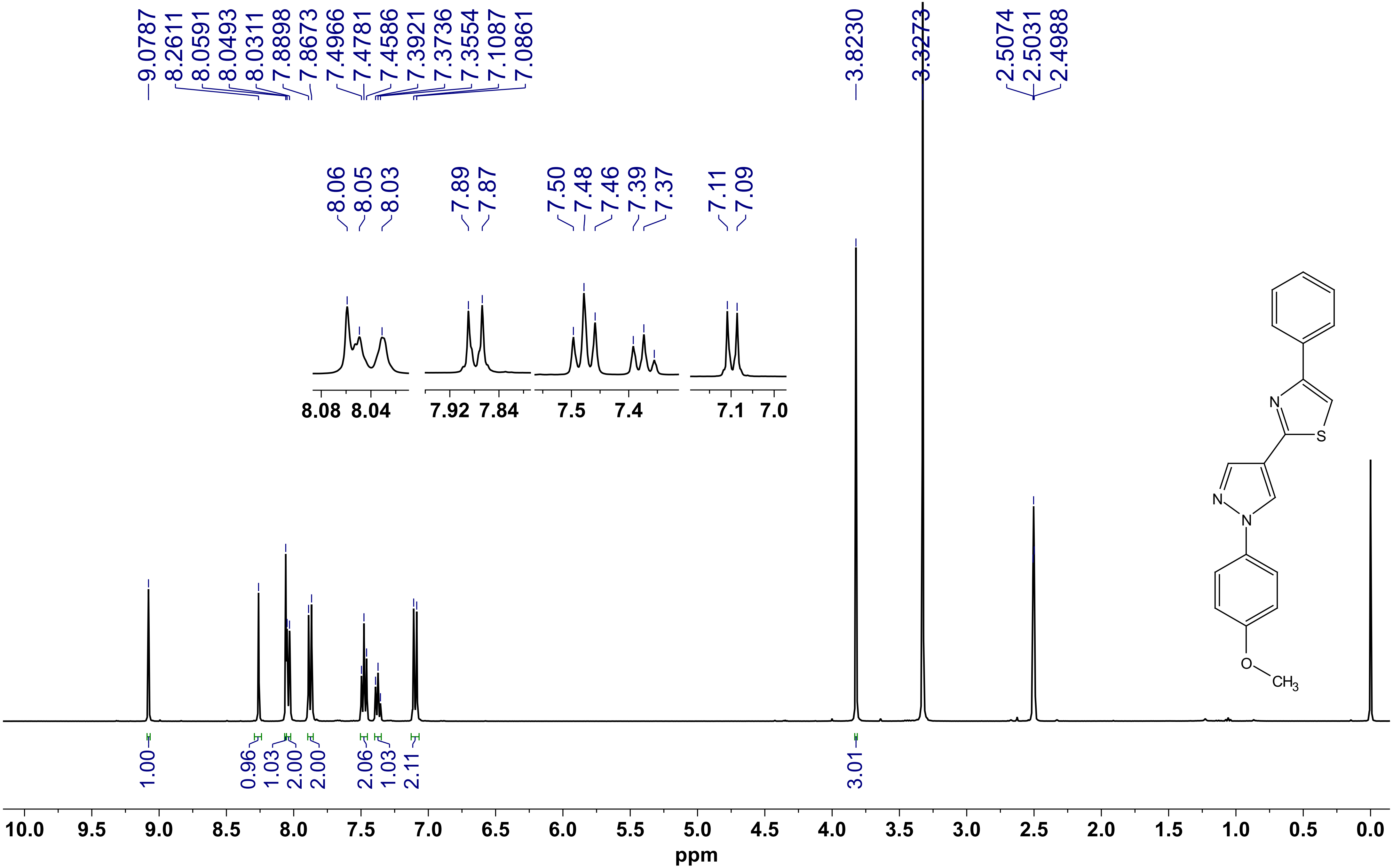

Figure S22: ^{1}H NMR of compound **1k**

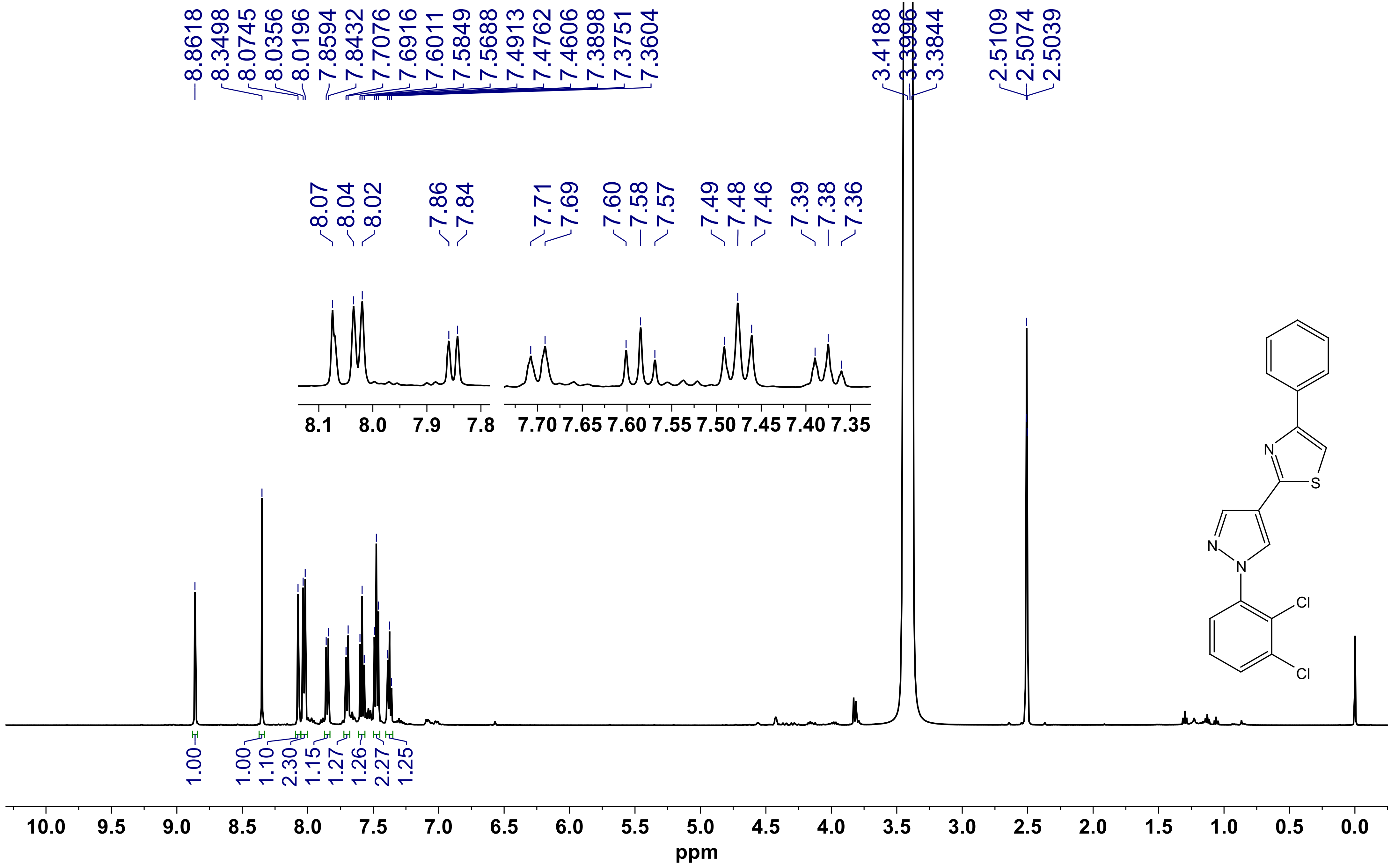

Figure S23: ^{13}C NMR of compound **1a**

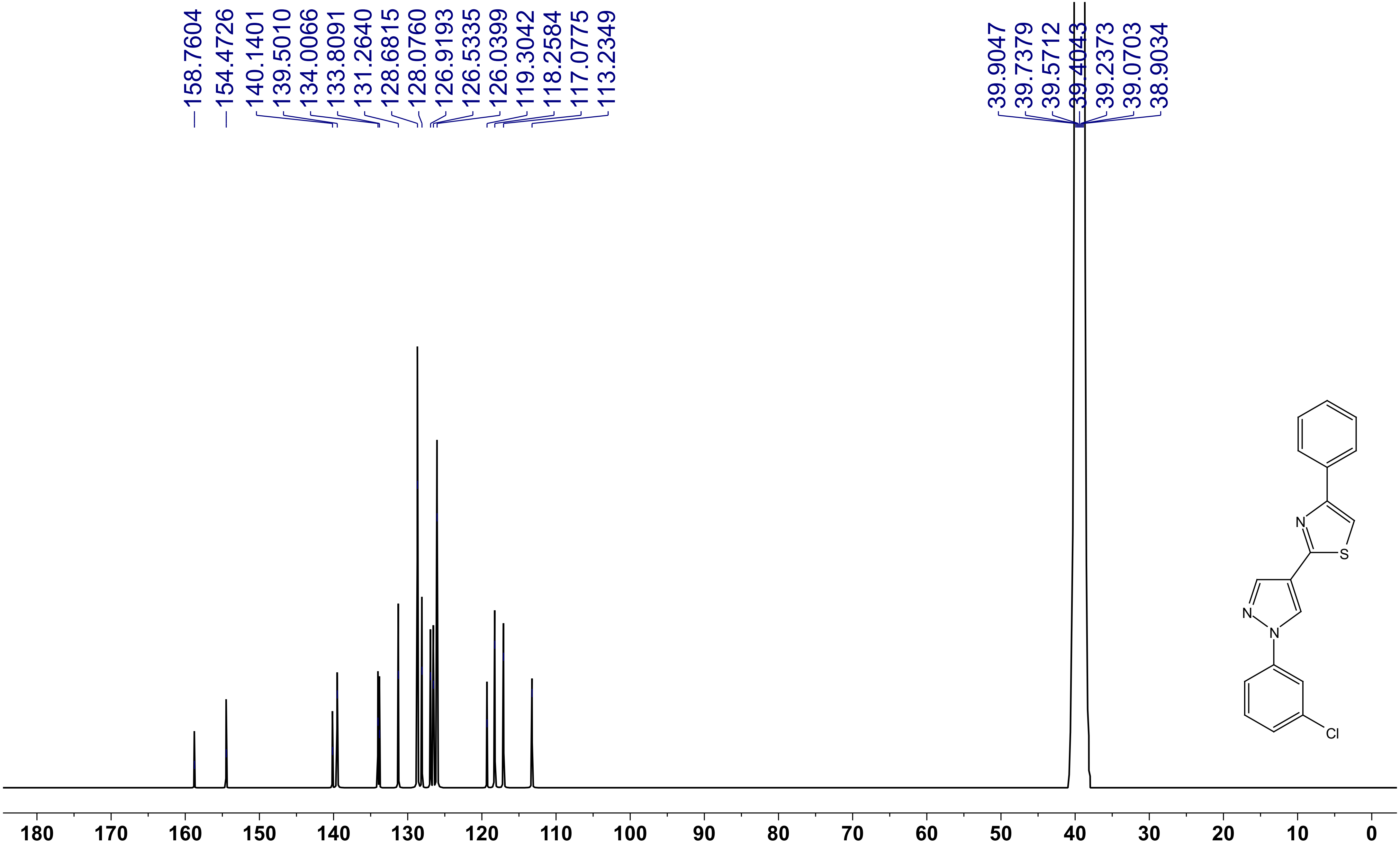

Figure S24: ^{13}C NMR of compound **1b**

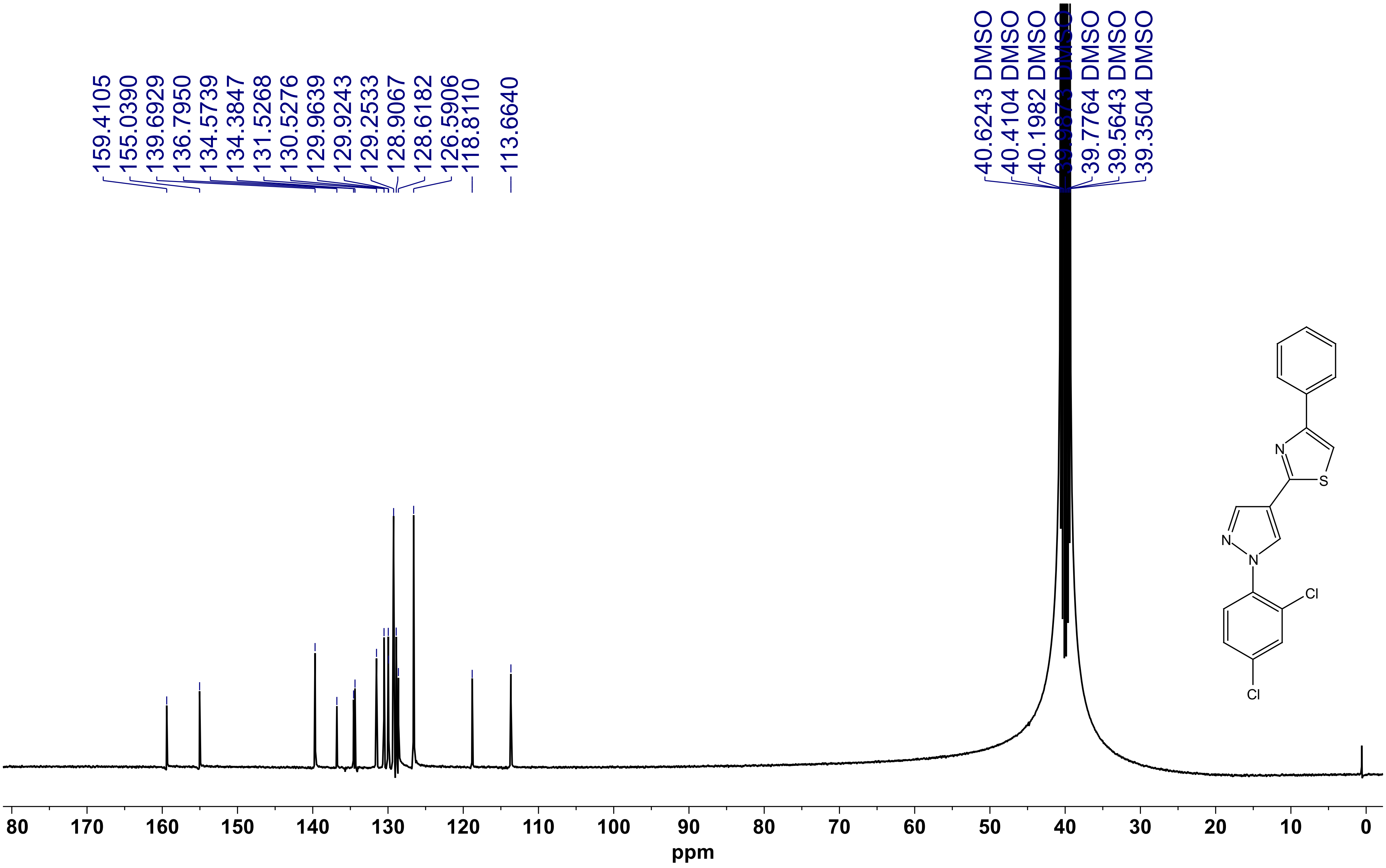

Figure S25: ^{13}C NMR of compound **1c**

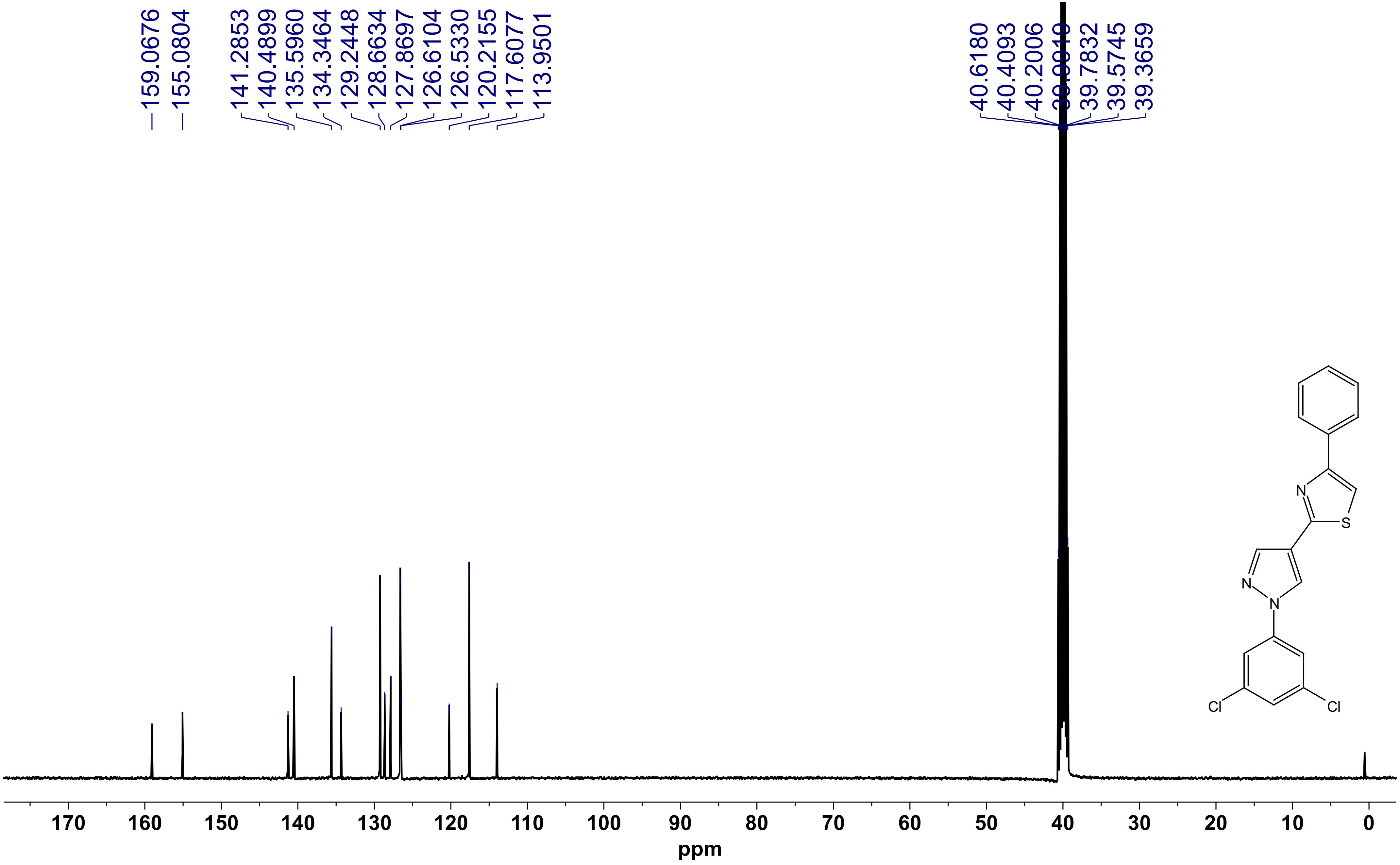

Figure S26: ^{13}C NMR of compound **1d**

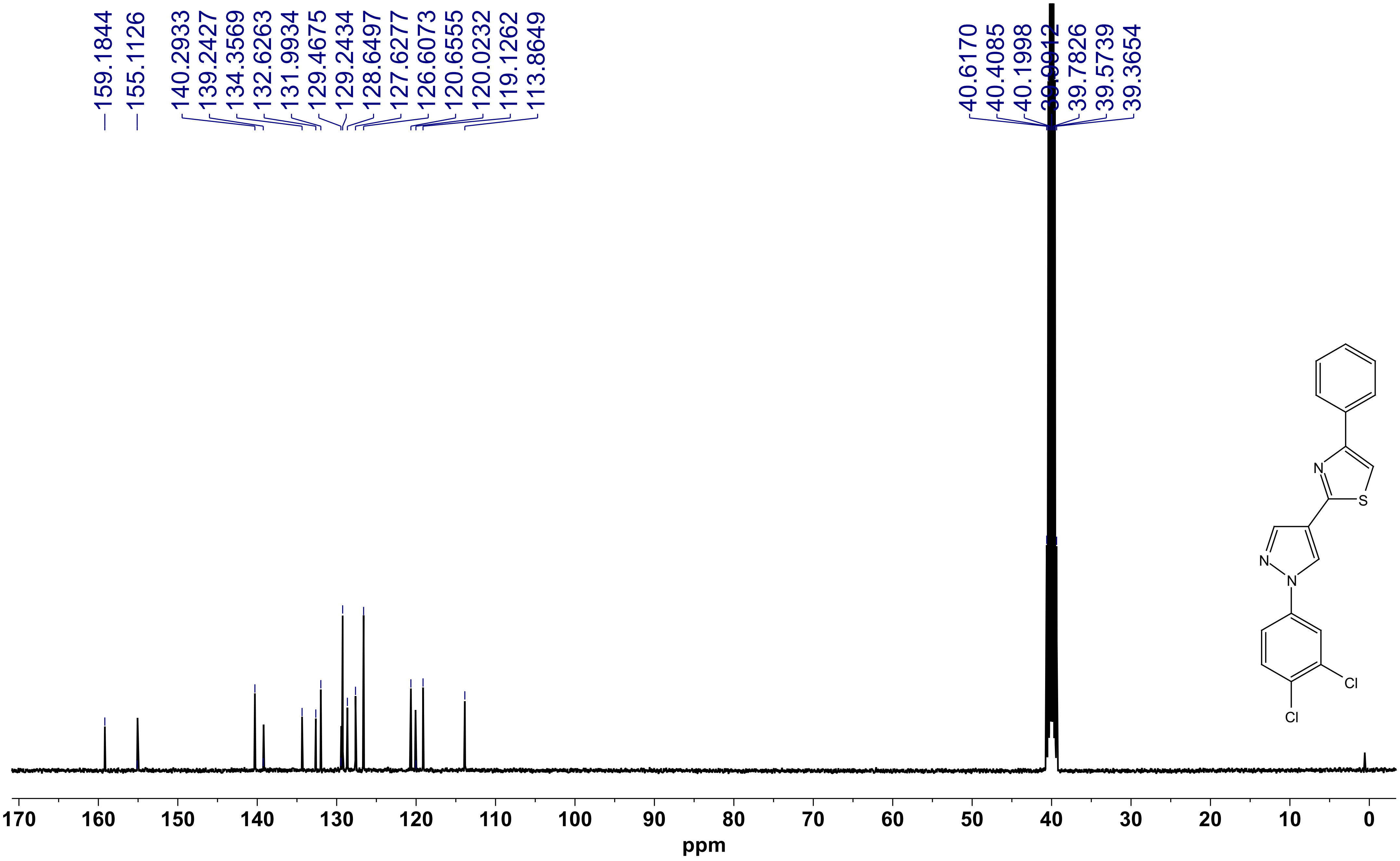

Figure S27: ^{13}C NMR of compound **1e**

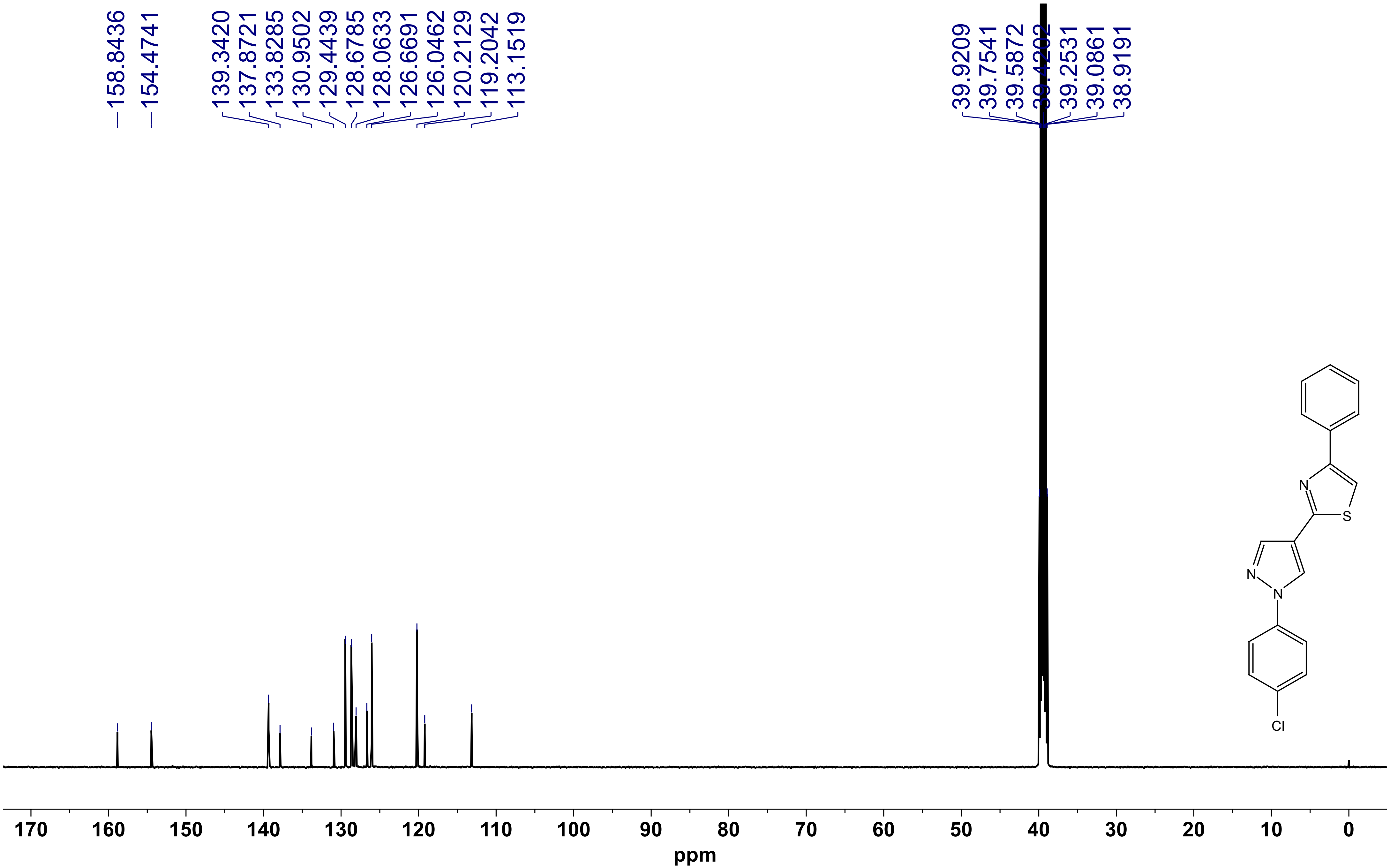

Figure S28: ^{13}C NMR of compound **1f**

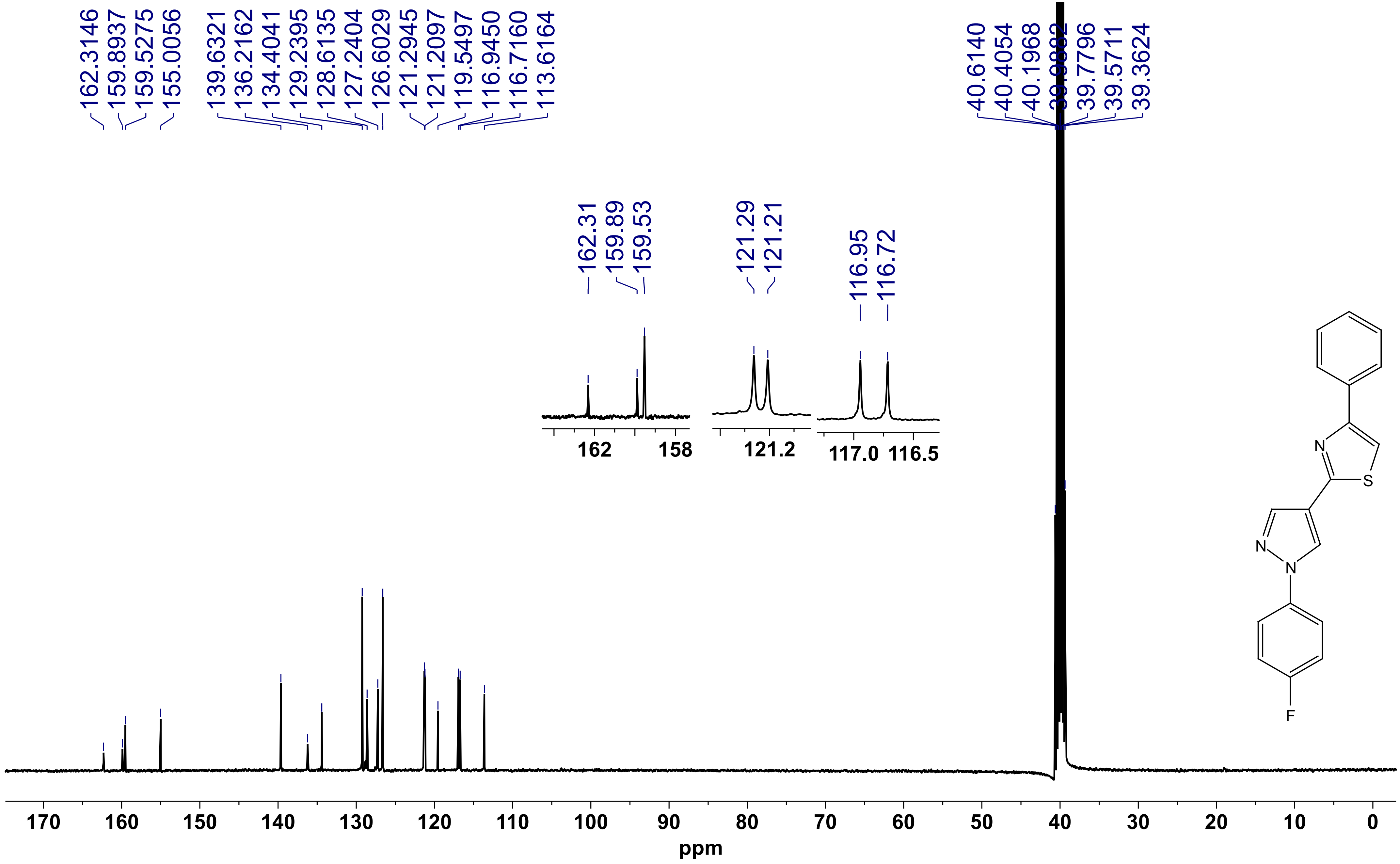

Figure S29: ^{13}C NMR of compound **1g**

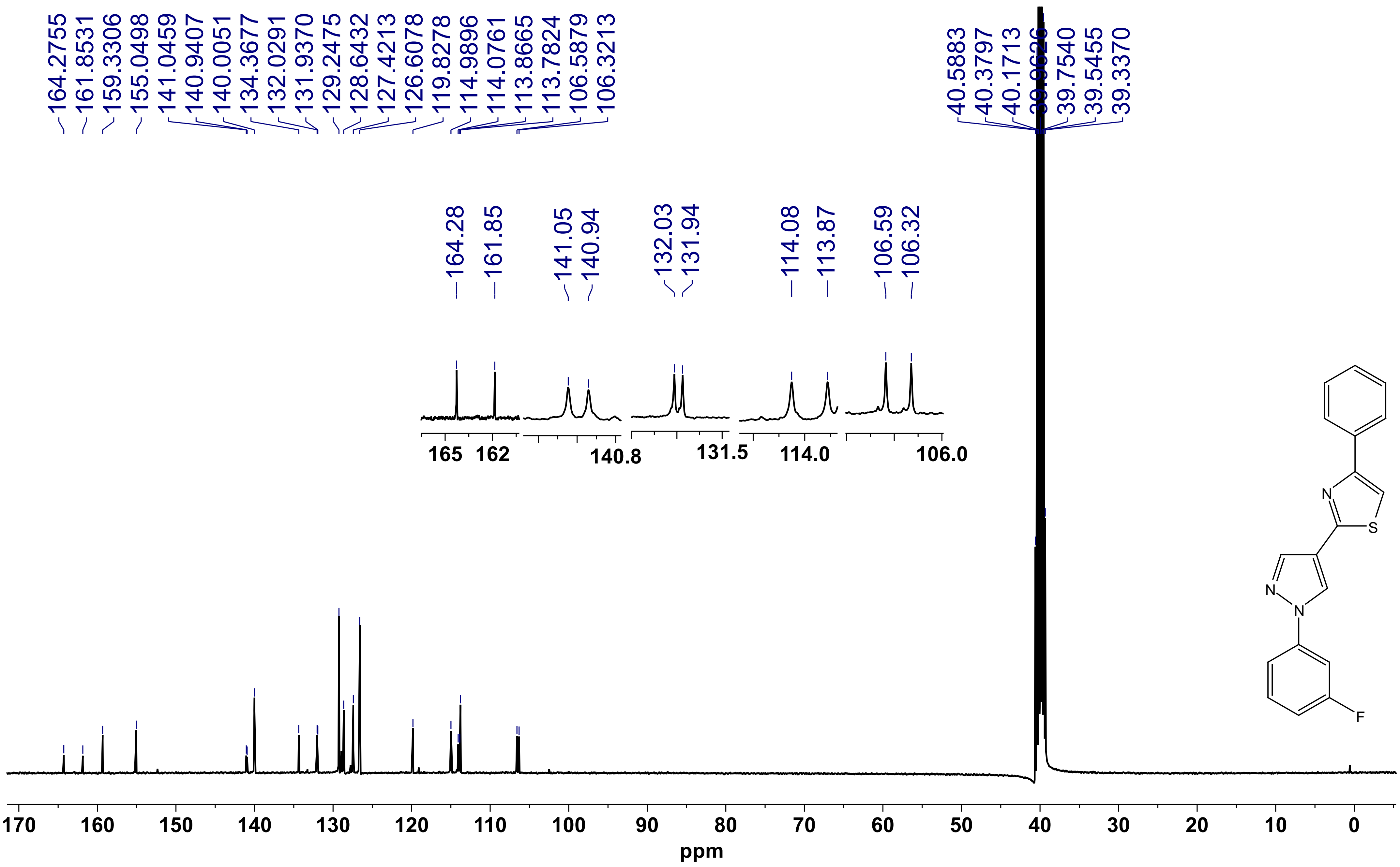

Figure S30: ^{13}C NMR of compound **1h**

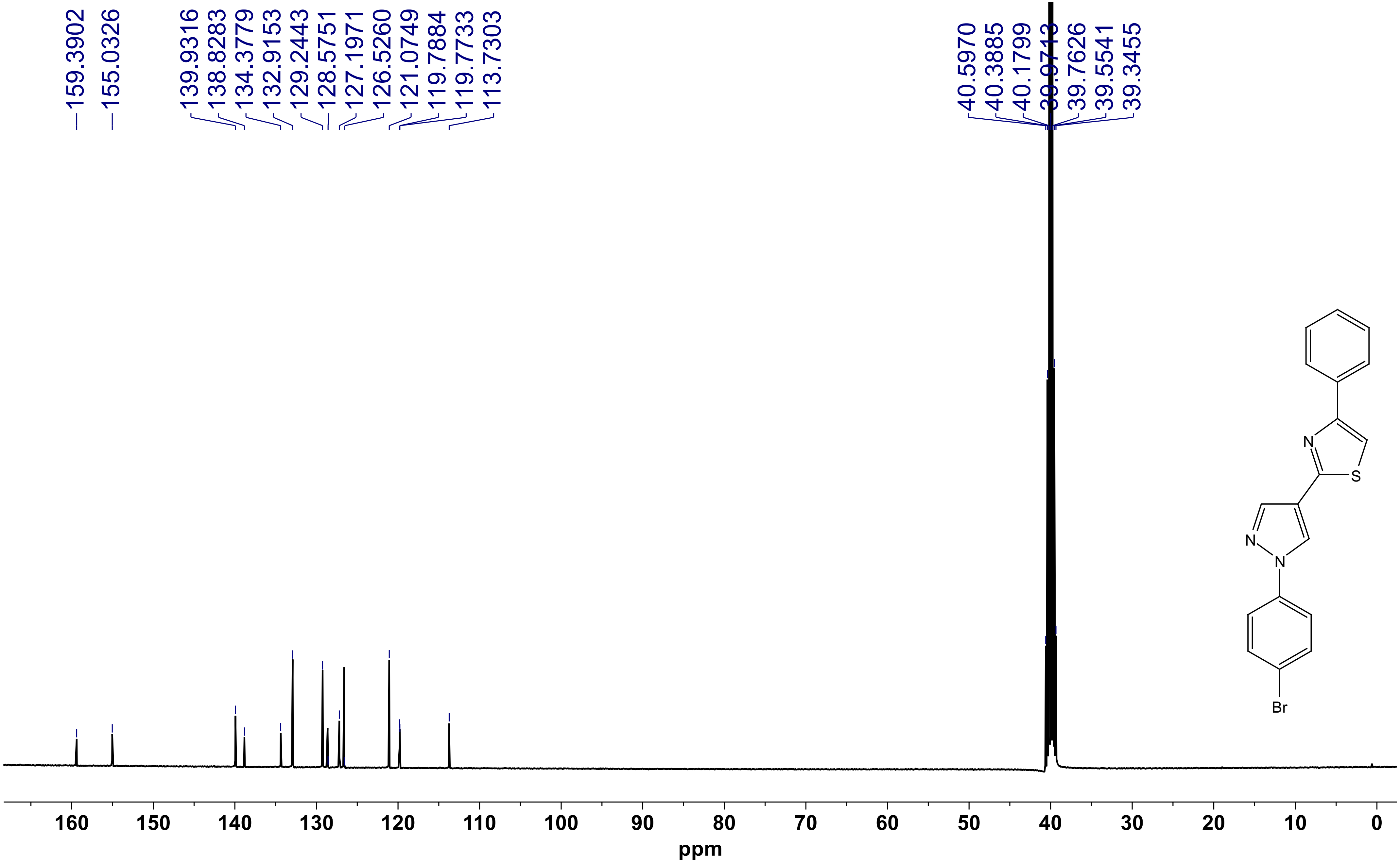

Figure S31: ^{13}C NMR of compound **1i**

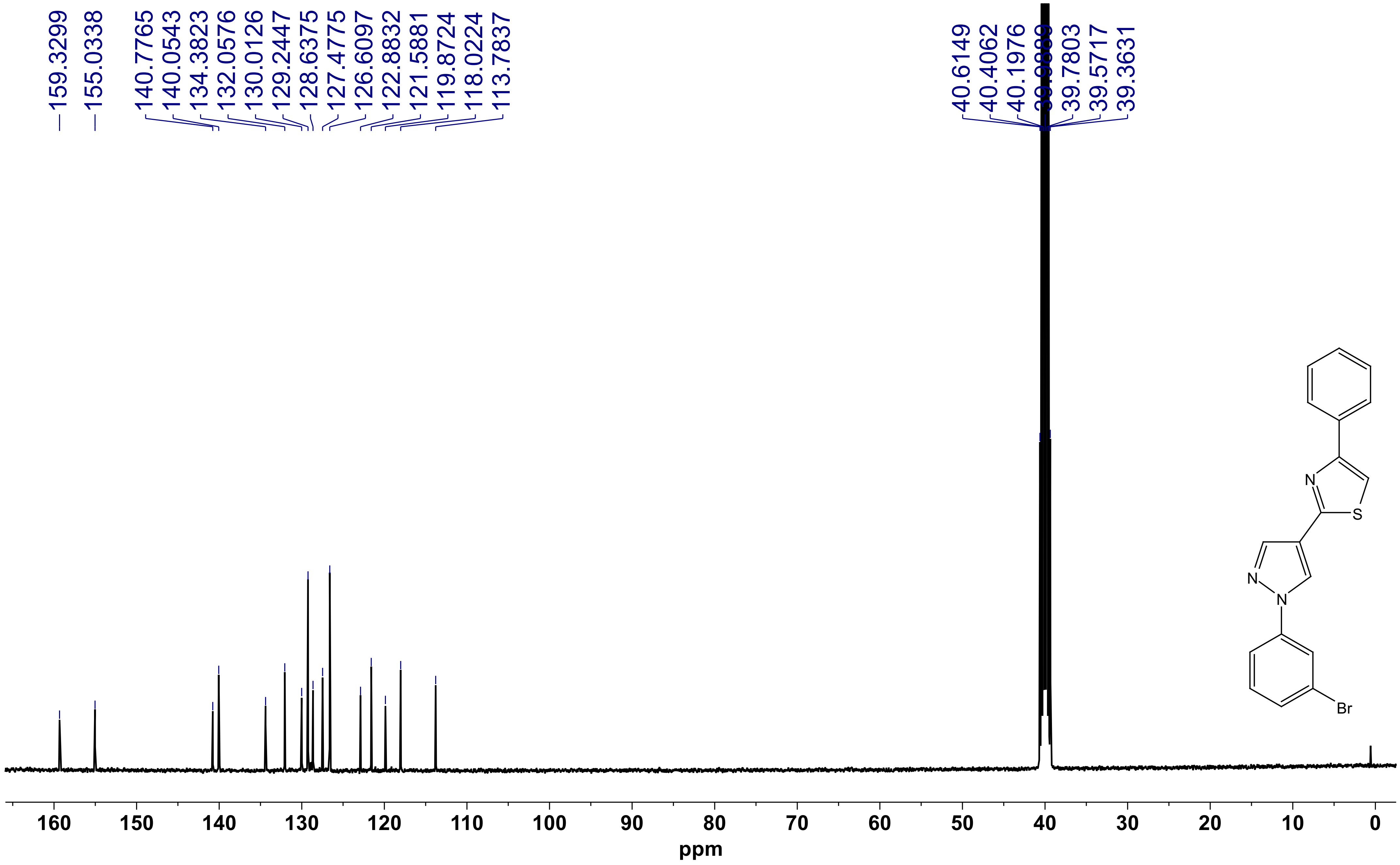

Figure S32: ^{13}C NMR of compound **1j**

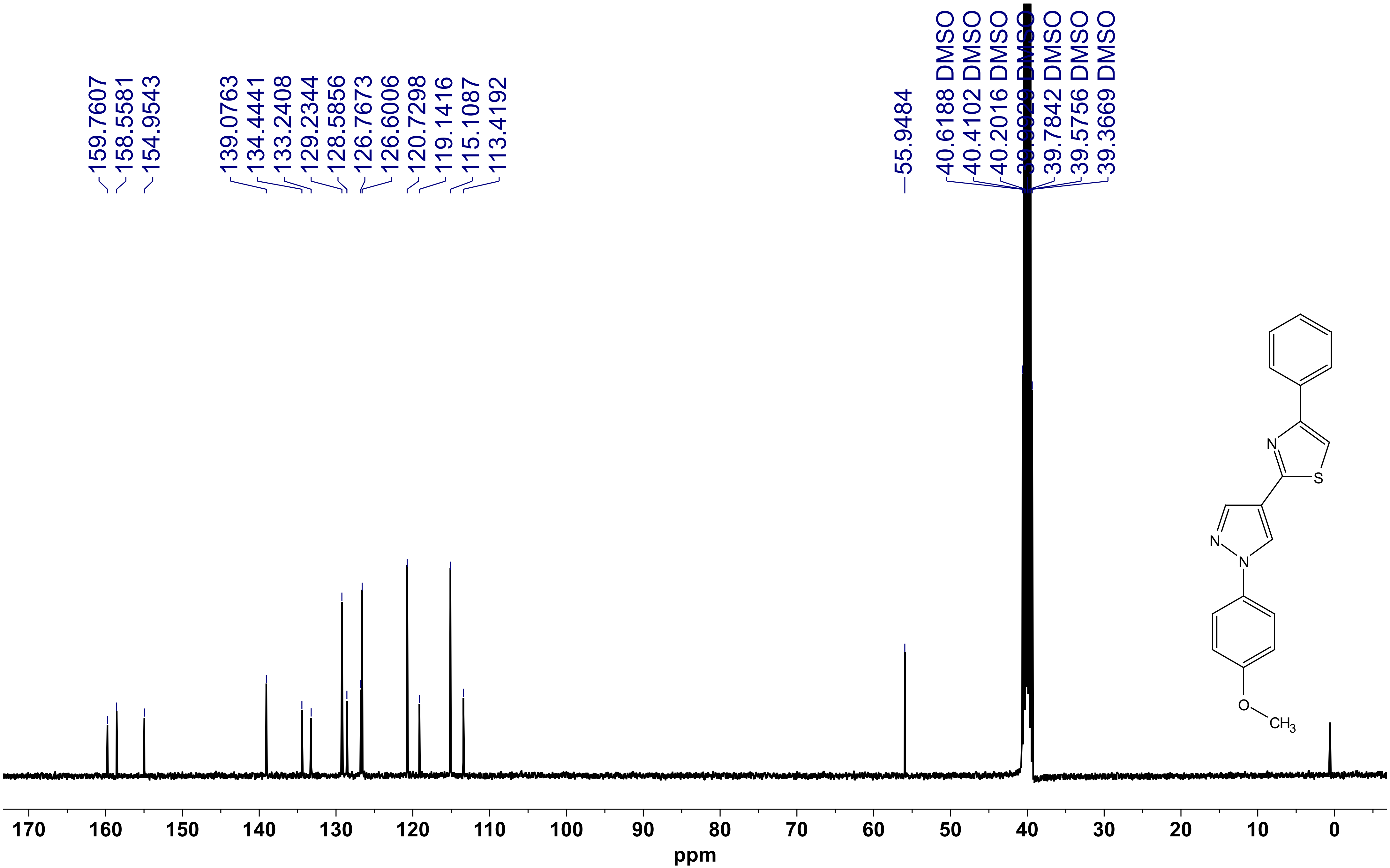

Figure S33: ^{13}C NMR of compound **1k**

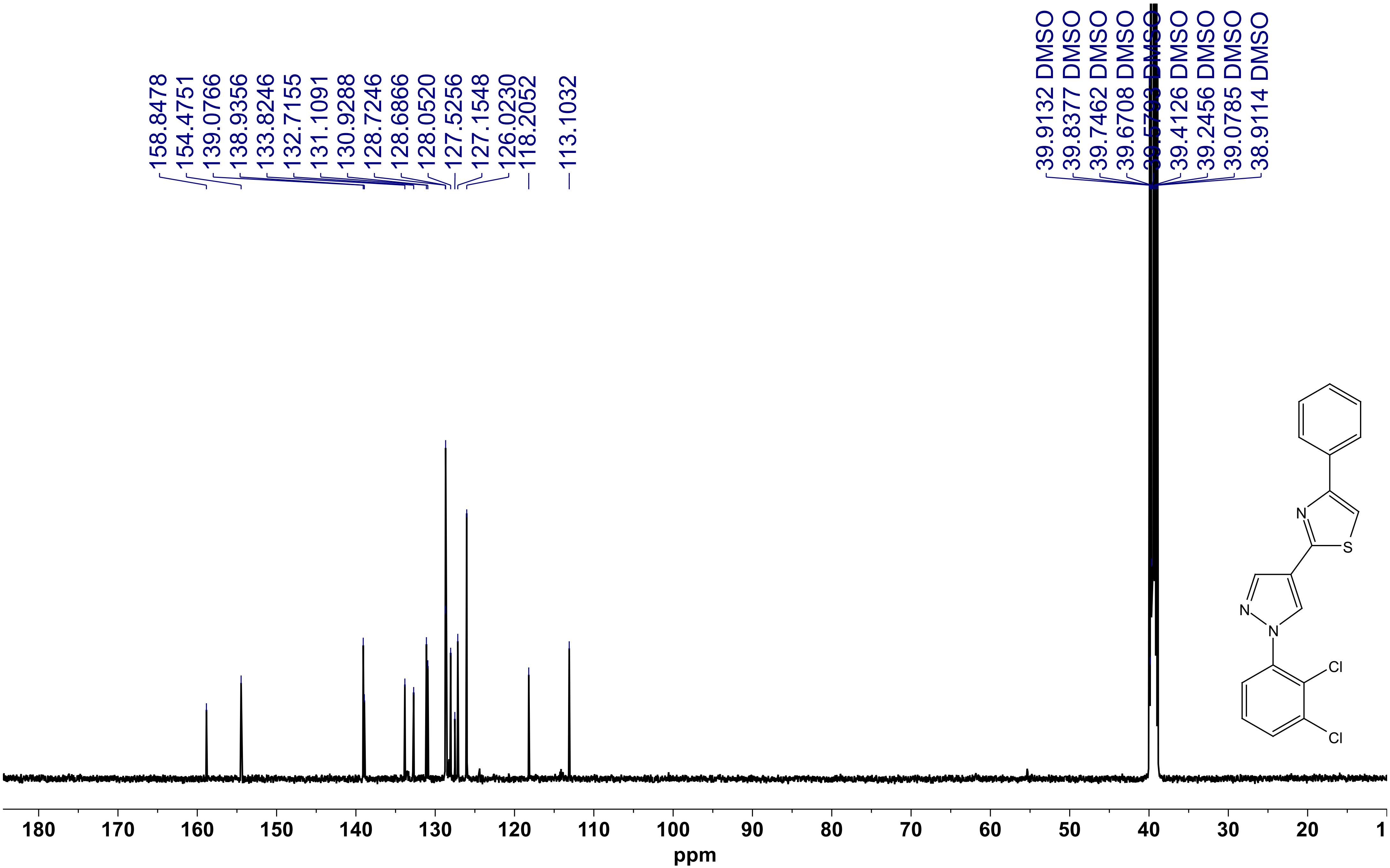

Figure S34: HRMS spectrum of compound **1a**

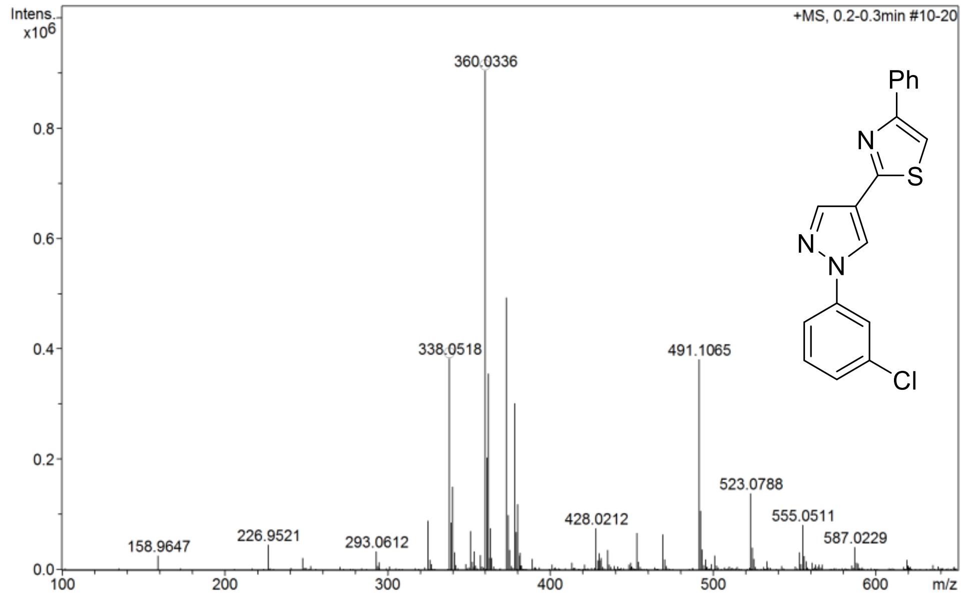

Figure S35: HRMS spectrum of compoud **1b**

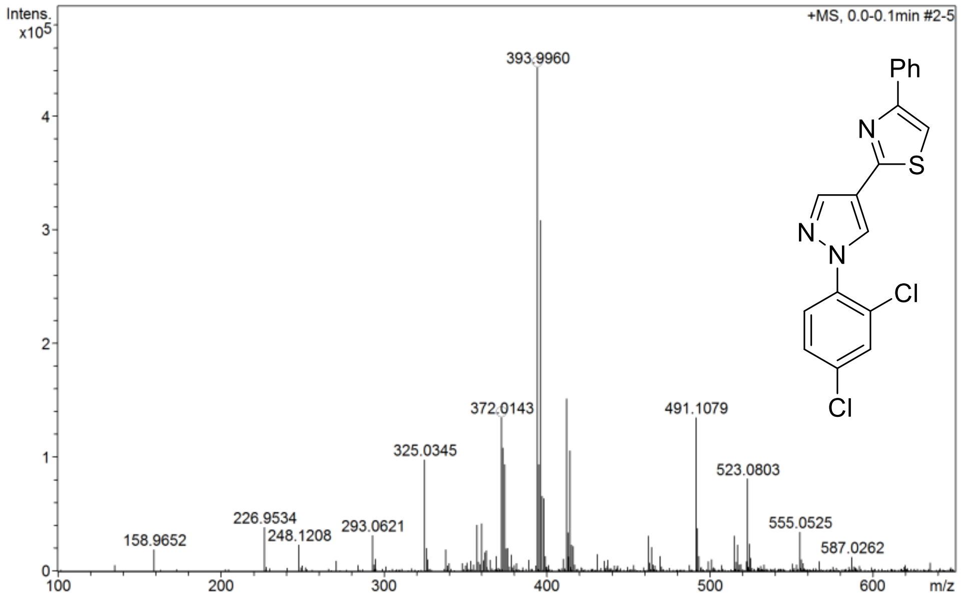

Figure S36: HRMS spectrum of compoud **1c**

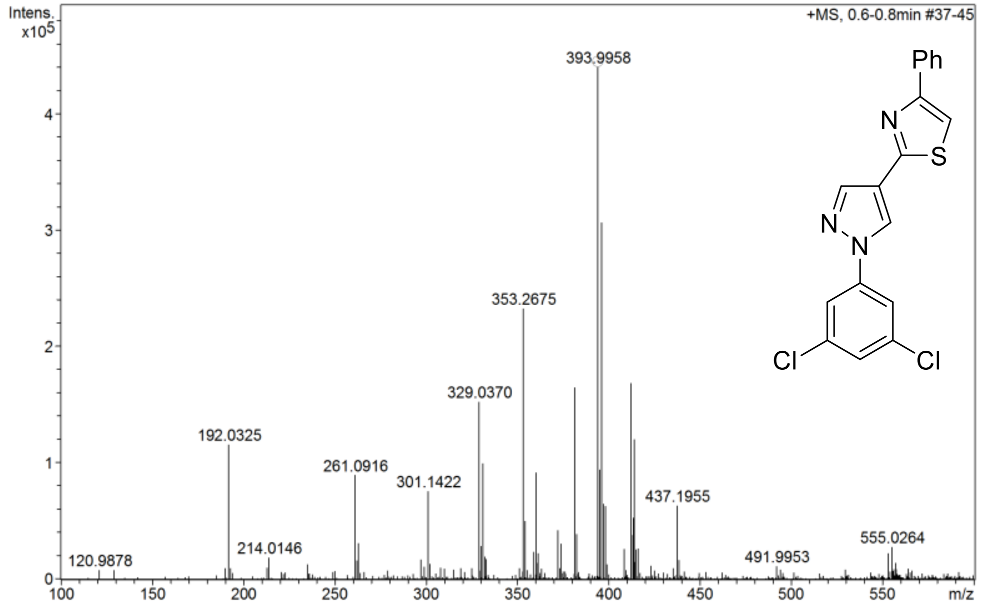

Figure S37: HRMS spectrum of compoud **1d**

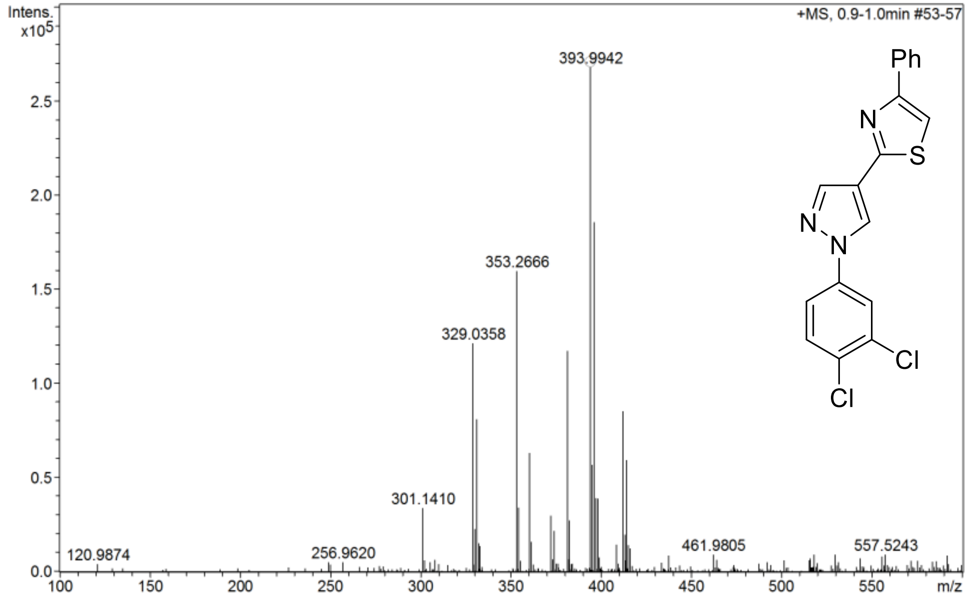

Figure S38: HRMS spectrum of compoud **1e**

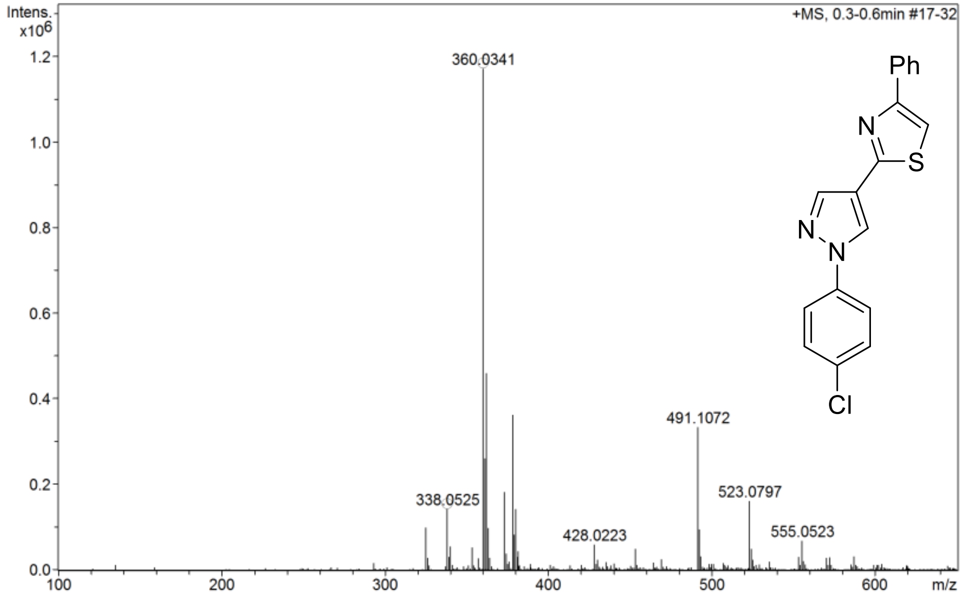

Figure S39: HRMS spectrum of compoud **1f**

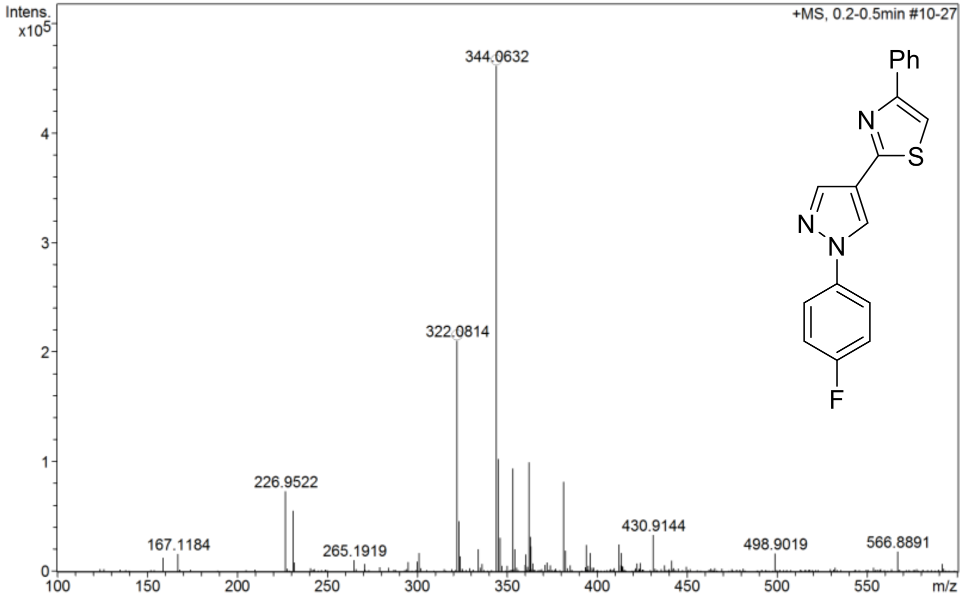

Figure S40: HRMS spectrum of compoud **1g**

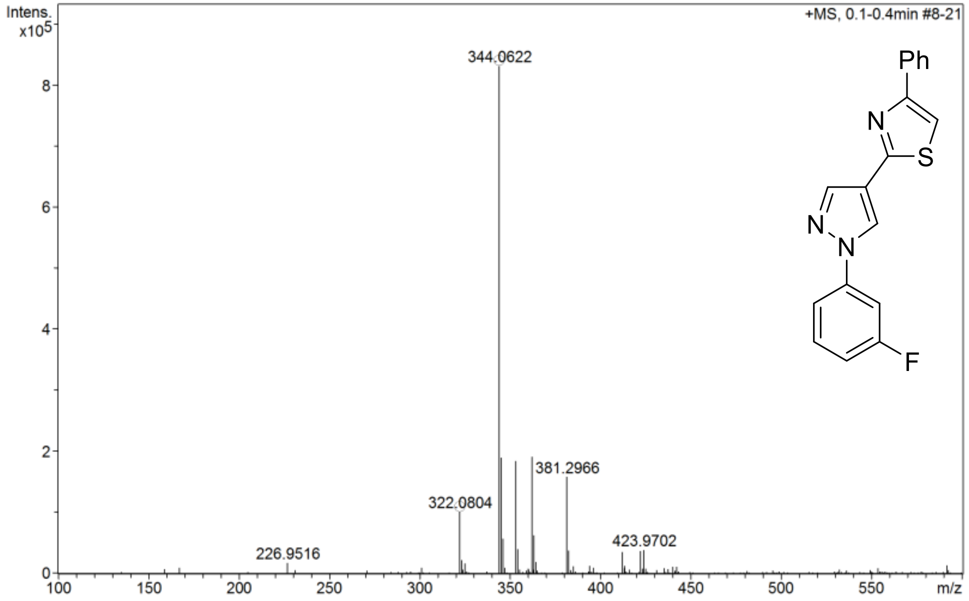

Figure S41: HRMS spectrum of compoud **1h**

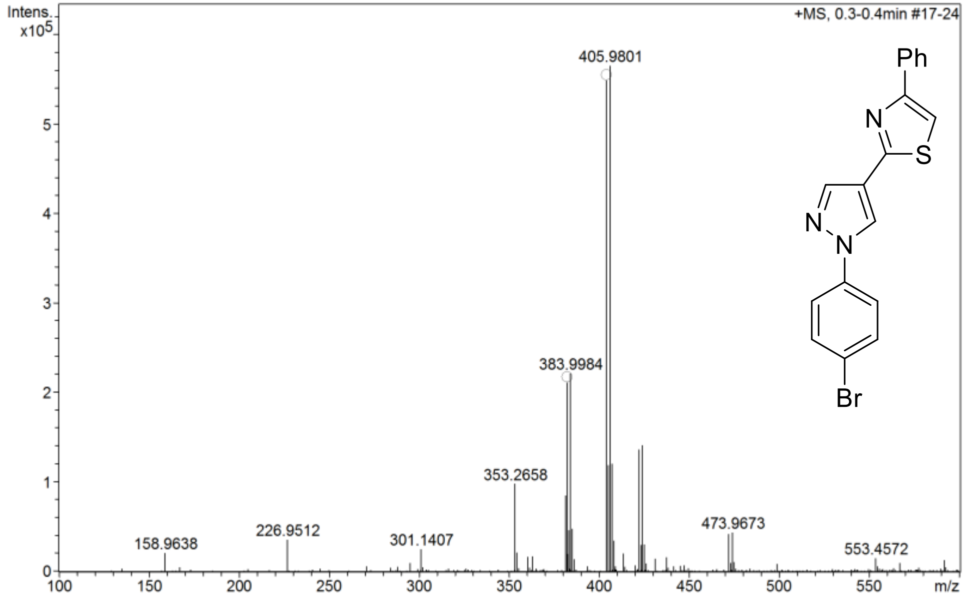

Figure S42: HRMS spectrum of compoud **1i**

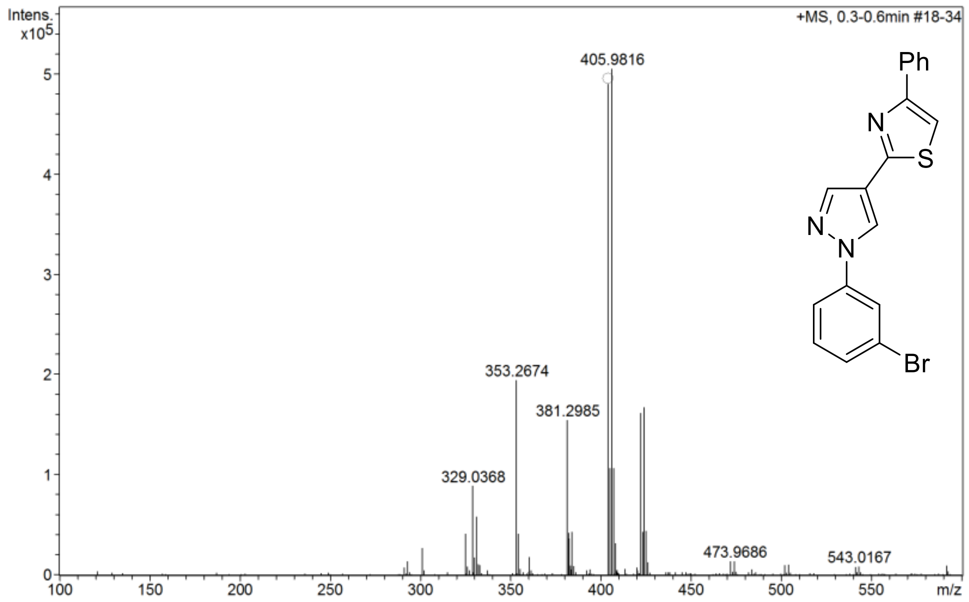

Figure S43: HRMS spectrum of compoud **1j**

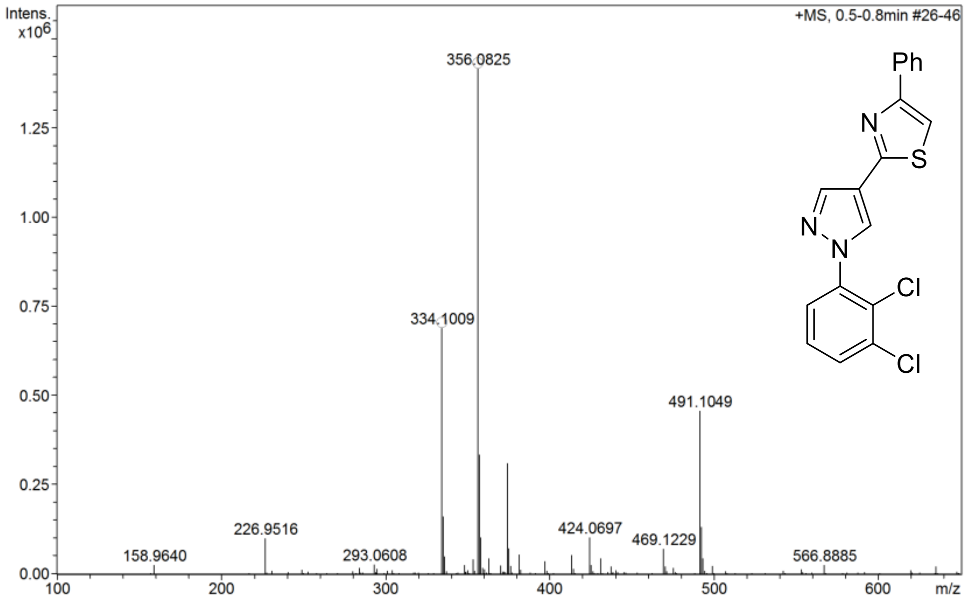

Figure S44: HRMS spectrum of compoud **1k**

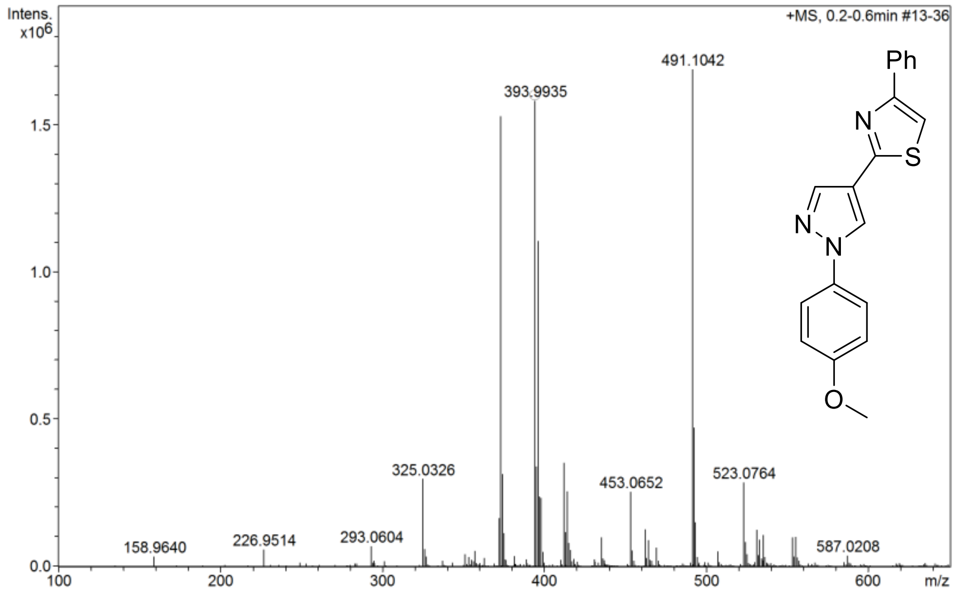

Supplement: Supplementary file 1 [file ao5c12469_si_001.pdf]
